# Supplementary material for: Producing aryl halides from lignin
Source: Nat Commun. 2025 Apr 17;16:3673. doi: 10.1038/s41467-025-59054-0 (PMC12006440; doi:10.1038/s41467-025-59054-0)
Supplement: Supplementary file 1 — Supplementary Information [file 41467_2025_59054_MOESM1_ESM.pdf]

Supplementary Information

Producing aryl halides from lignin

Yongqian Liu[1,2], Yi Li[1,2], Zhiyang He[1,2], Simeng Wu[1,2], Chunhui Ma[1,2], Wei Li[1,2], Shujun Li[1,2], Zhijun Chen[1,2]*, Shouxin Liu[1,2]*, Bing Tian[1,2]*

[1]State Key Laboratory of Utilization of Woody Oil Resource, College of Materials Science and Engineering, Northeast Forestry University, Harbin 150040, China
[2]Key Laboratory of Bio-based Material Science and Technology of Ministry of Education, Northeast Forestry University, Harbin 150040, China

E-mail: chenzhijun@nefu.edu.cn; liushouxin@126.com; tianbing@nefu.edu.cn

Table of Contents

Supplementary Tables 2

Supplementary Figures 10

Supplementary Methods 32

General Information 32

Preparation of lignin model compounds 32

General procedure for synthesis of lignin models 33

General procedure for depolymerization and halogenation of lignin models 35

Large-scale reaction 38

Native lignin extraction 39

General procedure for depolymerization and halogenation of native lignin 39

Gram scale experiment of depolymerization and halogenation of larch lignin 40

Identification and quantification of monomeric products from lignin halogenation depolymerization 41

Synthetic application of aryl halides 42

Supplementary Discussion 51

Investigation for activation of [X^+] reagent 51

Reactivity of lignin linkages for research of bond cleavage 52

Polysubstituted product formation 52

New compounds characterization 54

NMR Spectra 67

Supplementary References 109

Supplementary Tables

Supplementary Table 1. Solvent screening for bromination reaction[a]

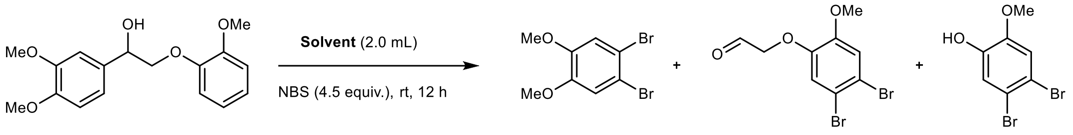

Entry	Solvent	Conversion (%)	Yield of **2a** (%)[b]	Yield of **3a** (%)[c]	Yield of **4a** (%)[c]
1	Acetone	0	0	0	0
2	1,4-Dioxane	0	0	0	0
3	Acetone/TFE (9:1)	70	51	32	10
4	Acetone/TFE (5:1)	75	63	36	11
5	Acetone/TFE (1:1)	85	73	47	14
6	TFE	99	99	61	15
7	H_2O	0	0	0	0
8	EtOH	< 5	< 5	0	< 2
9	*i*-PrOH	< 5	< 3	0	< 2
10	EtOAc	0	0	0	0
11	MeCN	0	0	0	0
12	DMSO	0	0	0	0
13	DCM	0	0	0	0
14	DMF	0	5	0	0
15	Methyl acetate	0	9	0	0
16	HFIP	73	58	35	12

[a]The reaction of **1a** was conducted on 0.2 mmol scale. [b]Yields determined by ^{1}H NMR spectroscopy of the crude mixture with CH_2Br_2 as an internal standard. [c]Yields determined by GC-MS with *n*-octadecane as an internal standard.

Supplementary Table 2. Bromination reagent screening[a]

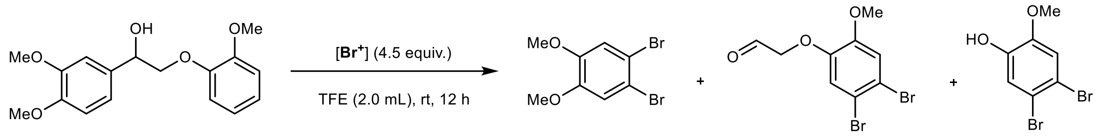

1a **2a** **3a** **4a**

Entry	[Br+]	Conversion (%)	Yield of **2a** (%)[b]	Yield of **3a** (%)[c]	Yield of **4a** (%)[c]
1	NBS	99	99	61	15
2	NBP	94	83	40	16
3[d]	DBDMH	86	77	33	14
4[e]	NDBI	21	5	trace	7

[a]The reaction of **1a** was conducted on 0.2 mmol scale. [b]Yields determined by ^{1}H NMR spectroscopy of the crude mixture with CH_2Br_2 as an internal standard. [c]Yields determined by GC-MS with *n*-octadecane as an internal standard. [d]DBDMH (2.0 equiv.). [e]NDBI (2.0 equiv.).

Supplementary Table 3. Chlorination reagent screening[a,b]

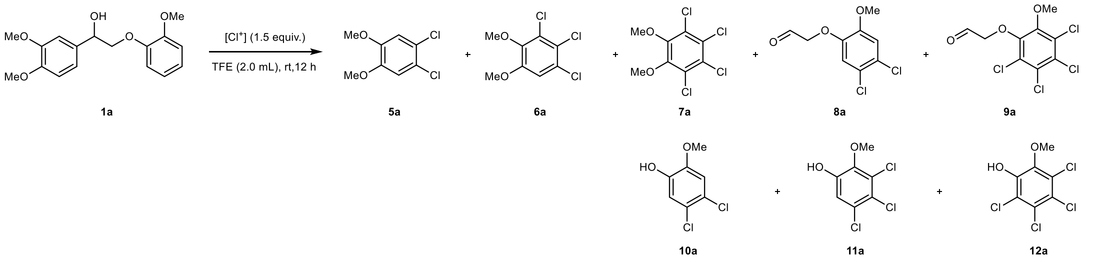

Entry	[Cl+]	Conversion (%)	Yield of **5a** (%)[b]	Yield of **6a** (%)[b]	Yield of **7a** (%)[b]	Yield of **8a** (%)[b]	Yield of **9a** (%)[b]	Yield of **10a** (%)[c]	Yield of **11a** (%)[c]	Yield of **12a** (%)[c]
1	NCS	12	3	0	0	0	0	0	0	0
2	DCDMH	14	2	0	0	0	0	0	0	0
3	Na-DCC	17	11	0	0	0	0	2	0	0
4	TCCA	82	48	22	0	32	0	18	0	0

[a]The reaction of **1a** was conducted on 0.2 mmol scale. [b]Yields determined by ^{1}H NMR spectroscopy of the crude mixture with CH_2Br_2 as an internal standard. [c]Yields determined by GC-MS with dodecane as an internal standard.

Supplementary Table 4. Optimization of chlorination reaction[a]

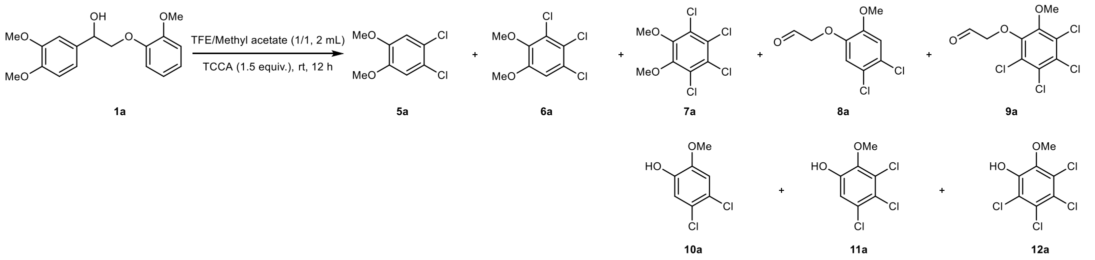

Entry	Condition changes	Conversion (%)	Yield of **5a** (%)[b]	Yield of **6a** (%)[b]	Yield of **7a** (%)[b]	Yield of **8a** (%)[b]	Yield of **9a** (%)[b]	Yield of **10a** (%)[c]	Yield of **11a** (%)[c]	Yield of **12a** (%)[c]
1	No change	91	58	30	0	22	0	9	6	0
2	TCCA (2.5 equiv.)	85	28	50	0	28	0	16	9	0
3	TCCA (2.5 equiv.)<br>TFE instead of TFE/Methyl acetate	86	0	0	78	10	14	3	5	10
4	TCCA (4.0 equiv.)	83	0	40	38	< 5	10	2	4	15
5	TCCA (4.0 equiv.)<br>TFE instead of TFE/Methyl acetate	99	0	0	92	0	34	0	0	20

[a]The reaction of **1a** was conducted on 0.2 mmol scale. [b]Yields determined by ^{1}H NMR spectroscopy of the crude mixture with CH_2Br_2 as an internal standard. [c]Yields determined by GC-MS with dodecane as an internal standard.

Supplementary Table 5. Lignin extraction from different woods.

wood	Mass of biomass	Mass of extracted lignin	Extracted lignin (wt%)
Larch	100 g	5.7 g	5.7
Scots pine	100 g	6.2 g	6.2
Korean pine	100 g	6.7 g	6.7
Chinese fir	100 g	5.4 g	5.4
Poplar	100 g	6.9 g	6.9
Birch	100 g	5.3 g	5.3
Beechwood	100 g	4.9 g	4.9

Supplementary Table 6. The monomer species in the extracted natural lignin and the halogenated products that may be obtained[a]

Lignin source	Type of wood	Aromatic residues	Brominated products[b]	Chlorinated products[c]
Larch	Soft	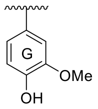 **Guaiacyl (G-)**	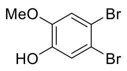 **4a**	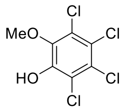 **12a**
Scots pine	Soft			
Korean pine	Soft			
Chinese fir	Soft			
Poplar	Hard	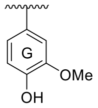 **Guaiacyl (G-)** 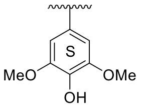 **Syringyl (S-)**	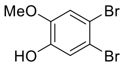 **4a** 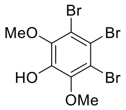 **20**	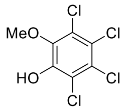 **12a** 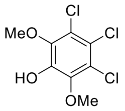 **21**
Birch	Hard			
Beechwood	Hard			

[a]Products were analyzed by GC-MS. [b]Yields of **4a** and **20** were determined by GC-MS with *n*-octadecane as the internal standard. [c]Yields of **12a** and **21** were determined by GC-MS with dodecane as the internal standard.

Supplementary Table 7. Retention times of monomeric products and internal standard

Compound	Retention time (min)
**4a**	14.578-14.708
**20**	21.317-21.869
**12a**	15.753-15.894
**21**	15.884-16.054
n-Octadecane (Internal standard)	15.743-16.576
Dodecane (Internal standard)	8.321-8.733

Supplementary Table 8. Monomer yields of lignin from various woods[a,b]

Lignin	Products of bromination and depolymerization		Products of chlorinatio and depolymerization	
	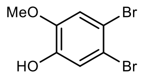 **4a**, wt%	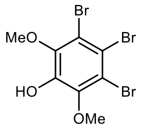 **20**, wt%	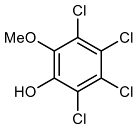 **12a**, wt%	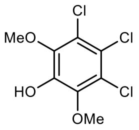 **21**, wt%
Larch	0.280	/	2.497	/
Scots pine	0.237	/	2.225	/
Korean pine	0.329	/	0.914	/
Chinese fir	0.150	/	1.995	/
Poplar	0.254	0.198	1.183	0.638
Birch	0.118	0.201	0.656	0.384
Beechwood	0.222	0.117	0.630	0.768

[a]The reaction of lignin was conducted on 100 mg scale. [b]The yield of the halogenated polymerization product is determined by GC-MS.

Supplementary Figures

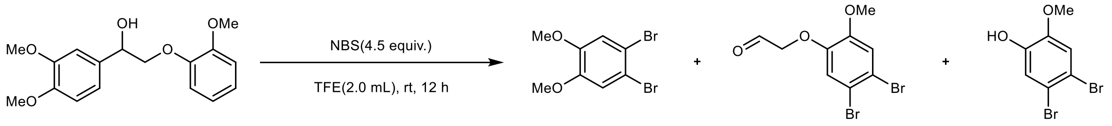

1a, 0.2 mmol **2a**, 99% **3a**, 61% **4a**, 15%

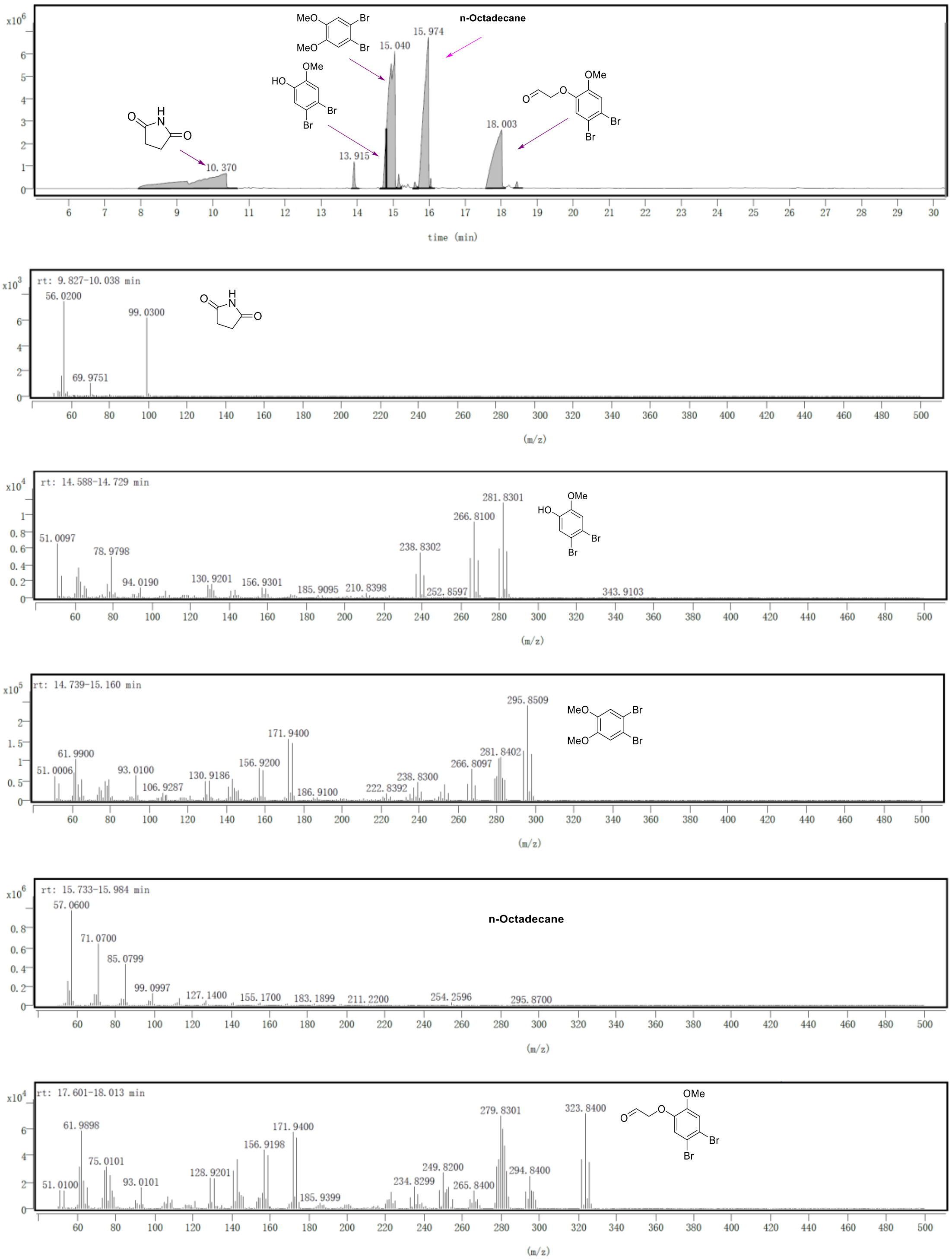

Supplementary Figure 1. GC-MS analysis of reaction of depolymerization and bromination of **1a**.

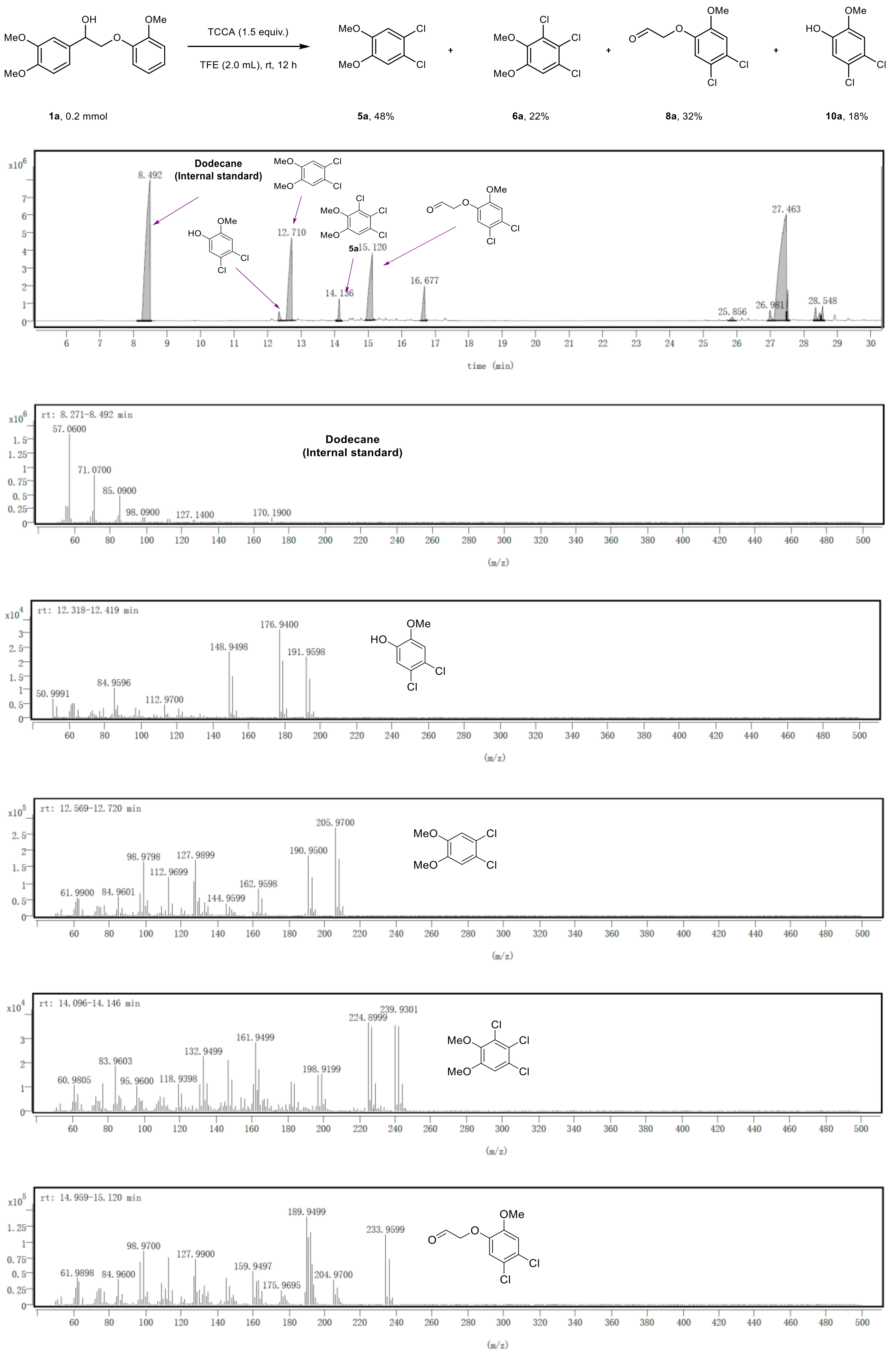

Supplementary Figure 2. GC-MS analysis of reaction of depolymerization and chlorination of **1a** under conditions of TCCA (1.5 equiv.) and TFE.

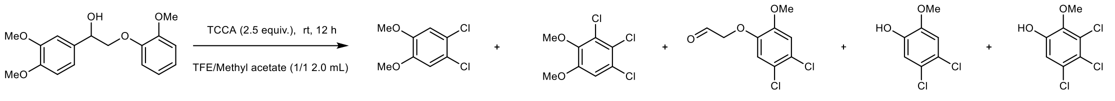

1a, 0.2 mmol **5a**, 28% **6a**, 50% **8a**, 28% **10a**, 16% **11a**, 9%

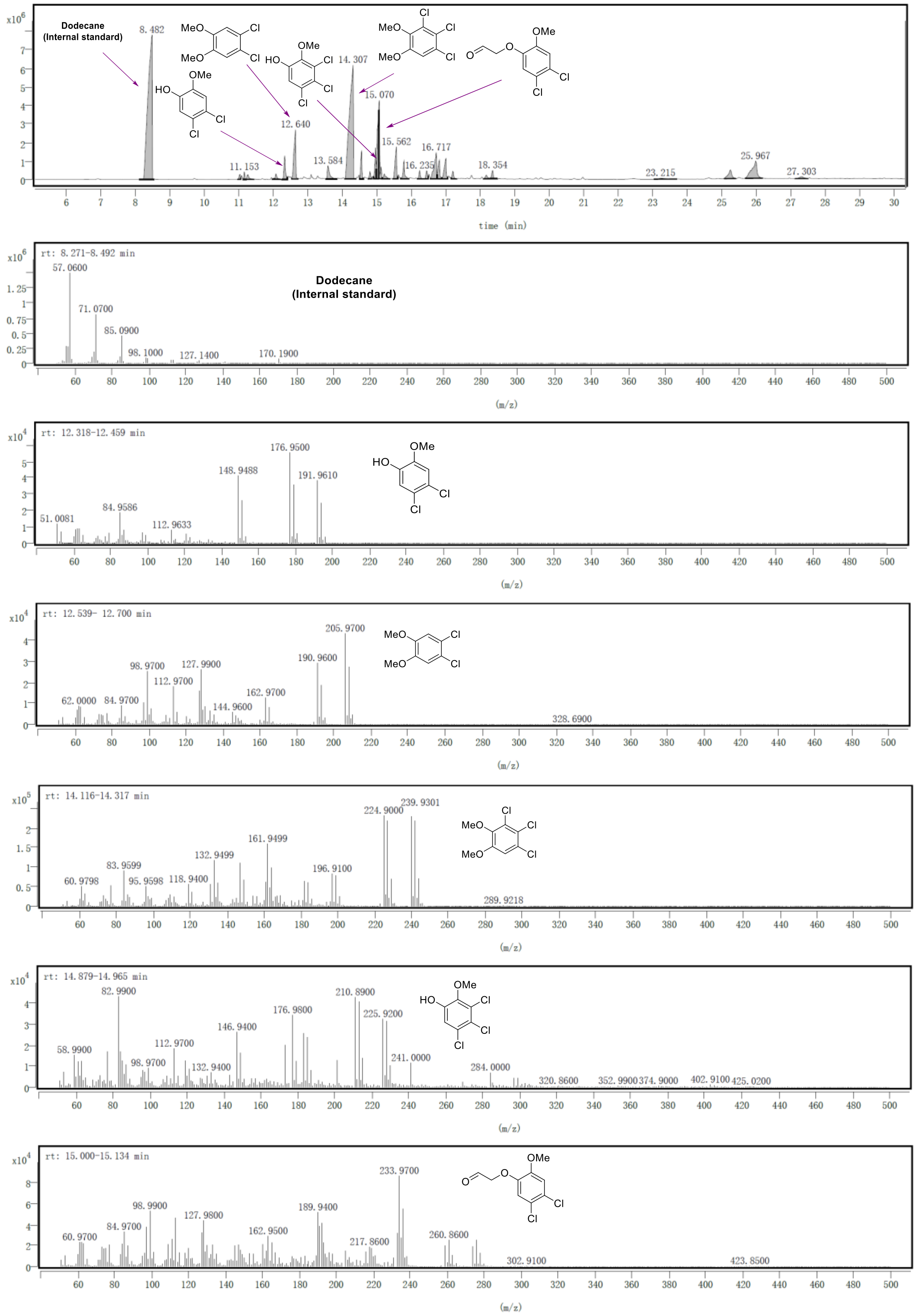

Supplementary Figure 3. GC-MS analysis of reaction of depolymerization and chlorination of **1a** under conditions of TCCA (2.5 equiv.) and TFE/methyl acetate.

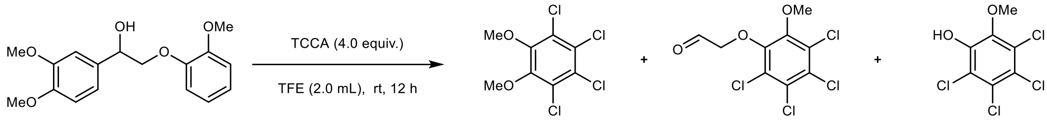

1a, 0.2 mmol **6a**, 92% **9a**, 34% **12a**, 20%

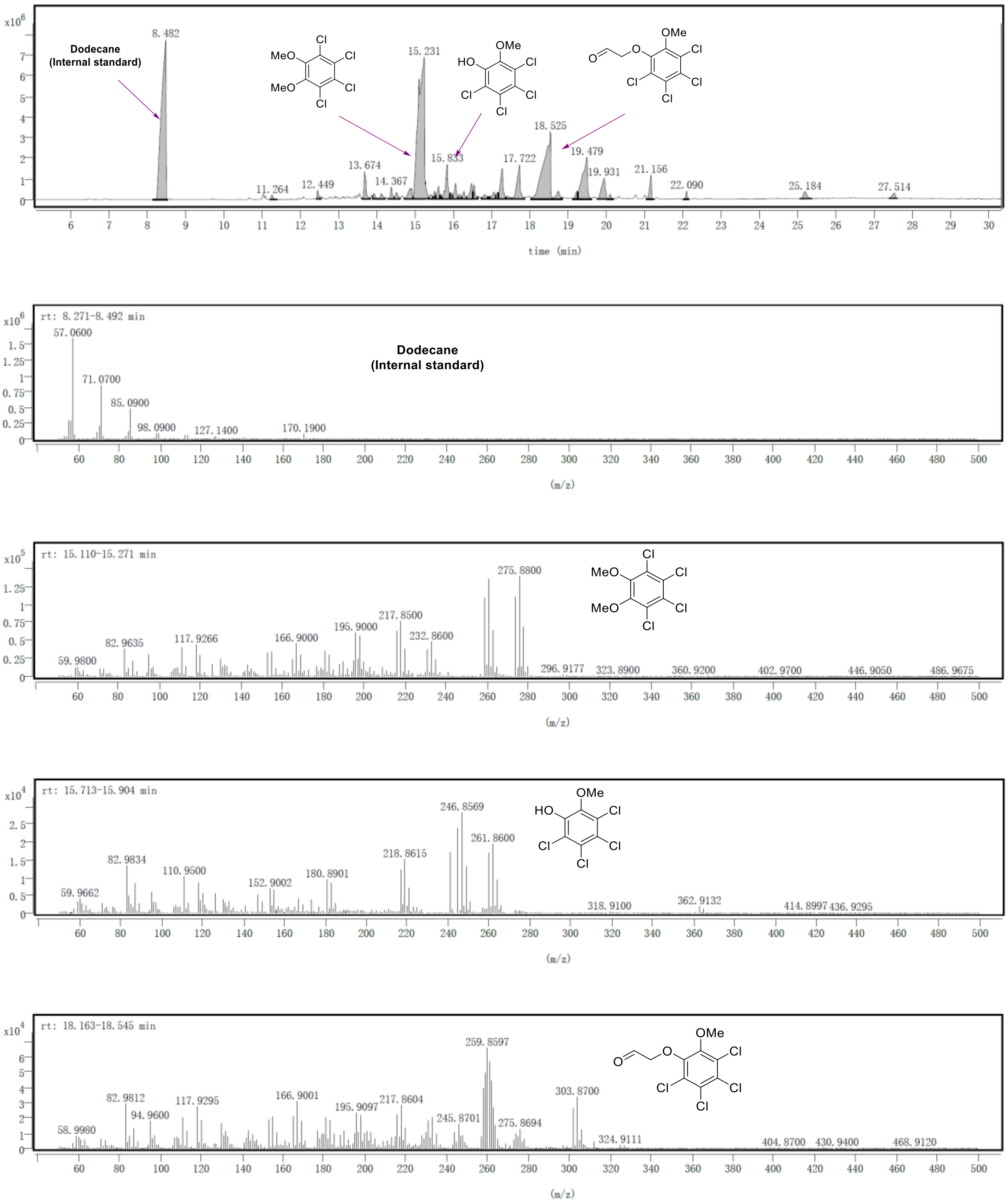

Supplementary Figure 4. GC-MS analysis of reaction of depolymerization and chlorination of **1a** under conditions of TCCA (4.0 equiv.) and TFE.

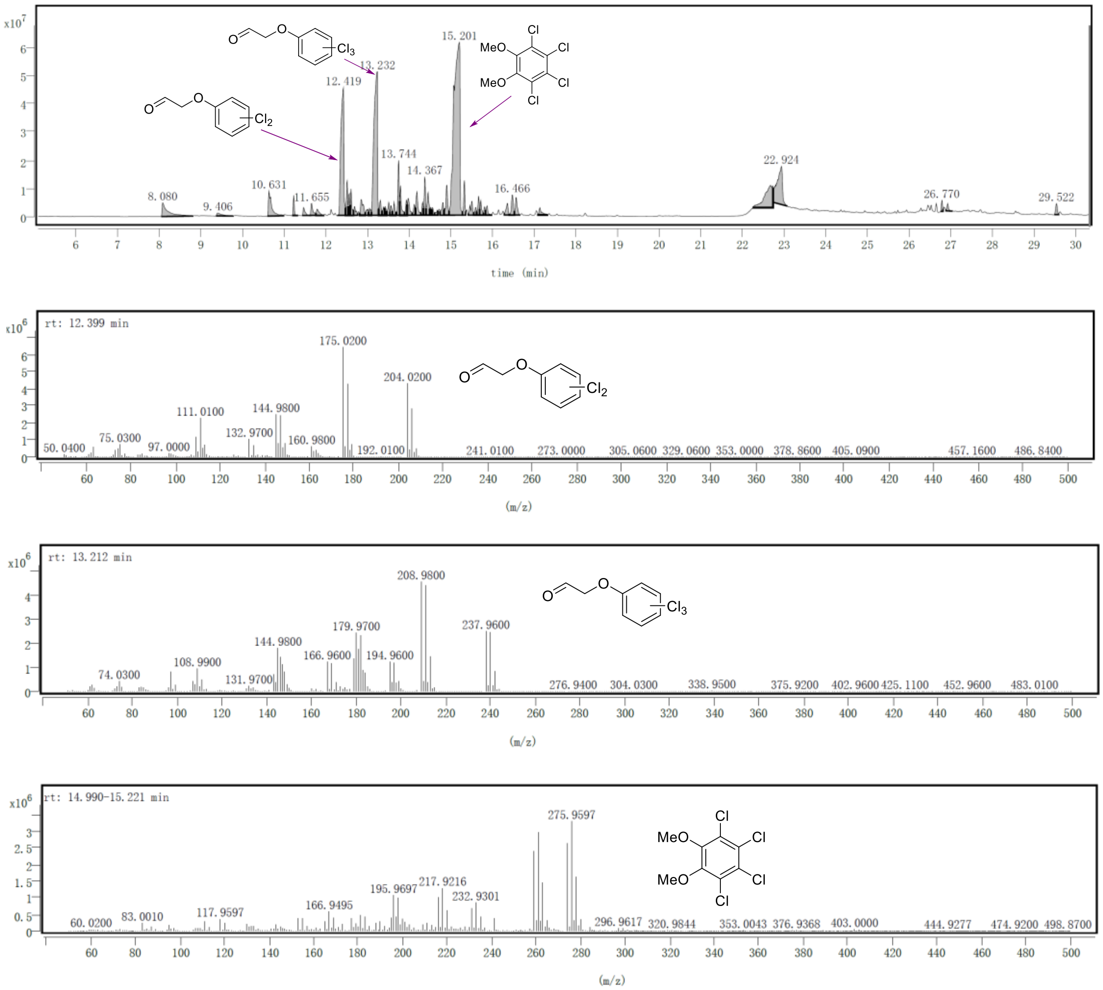

Supplementary Figure 5. GC-MS analysis of reaction of depolymerization and chlorination of **1b**.

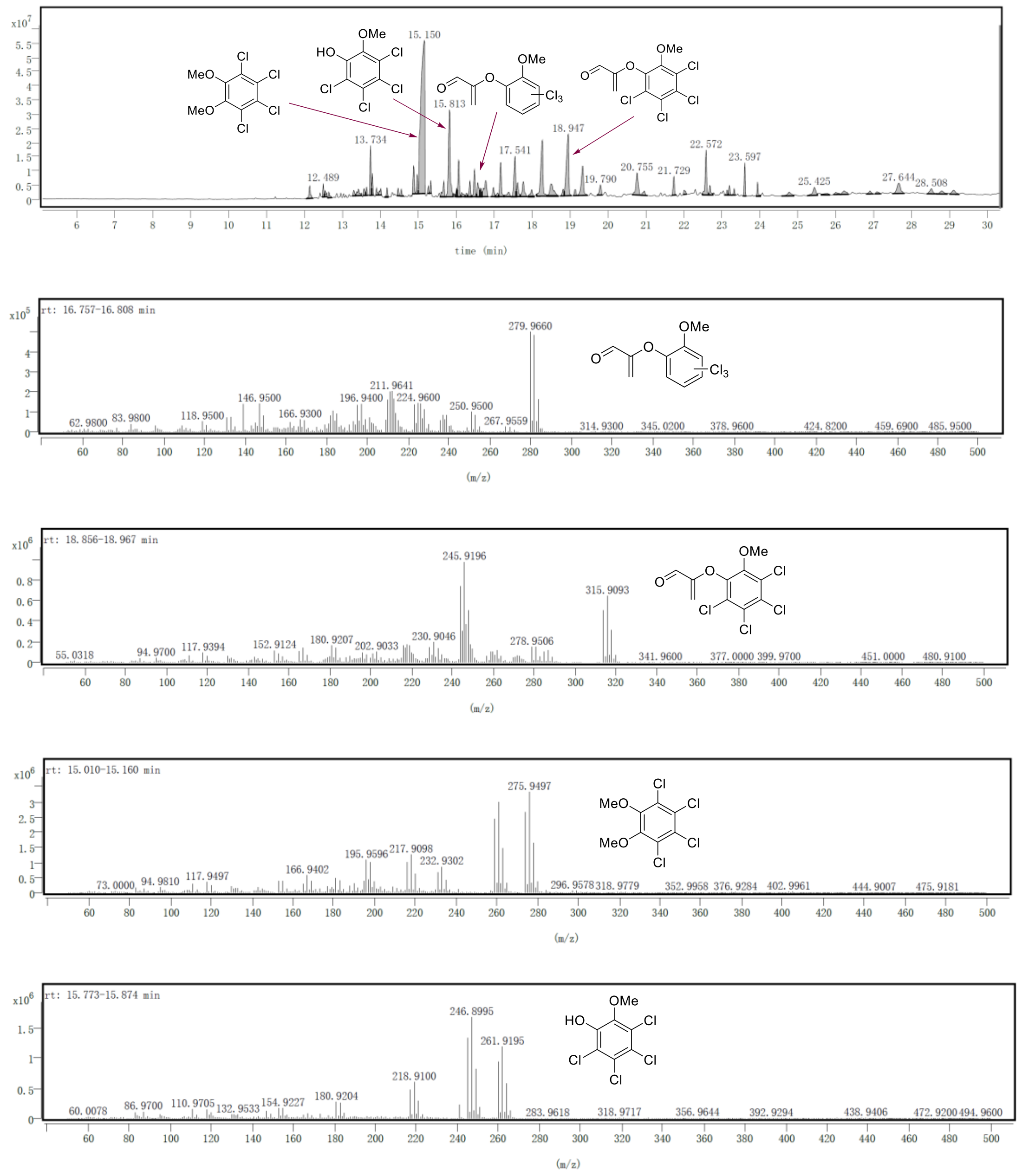

Supplementary Figure 6. GC-MS analysis of reaction of depolymerization and chlorination of **1c**.

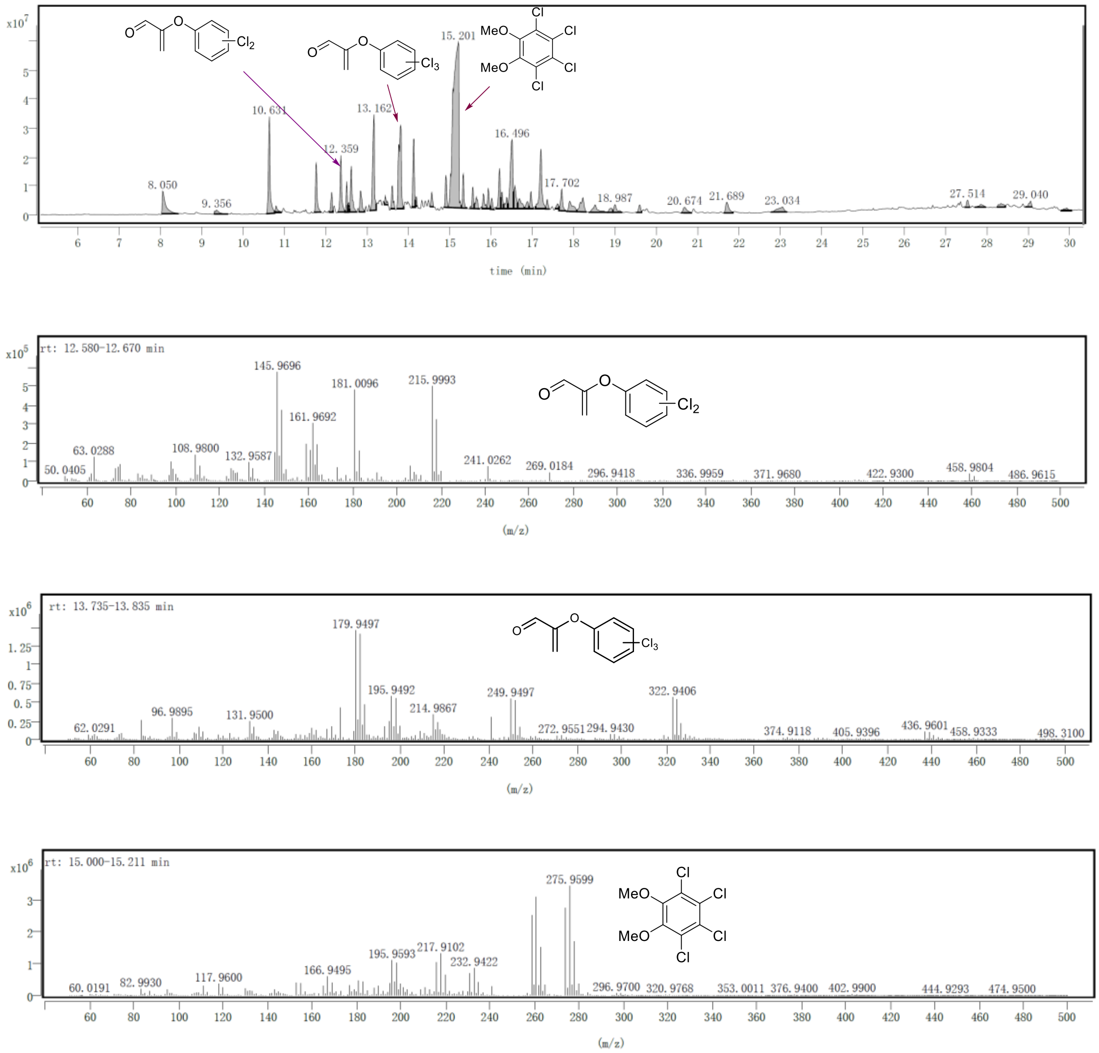

Supplementary Figure 7. GC-MS analysis of reaction of depolymerization and chlorination of **1d**.

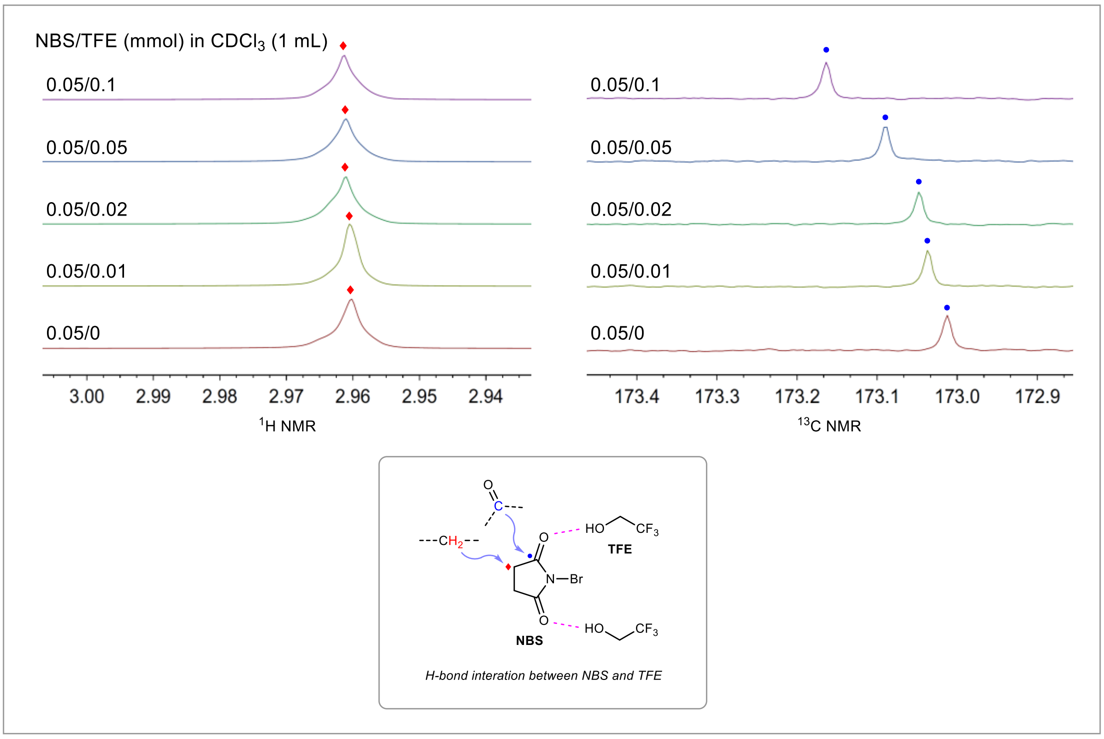

Supplementary Figure 8. Activation of NBS by TFE through hydrogen bonding.

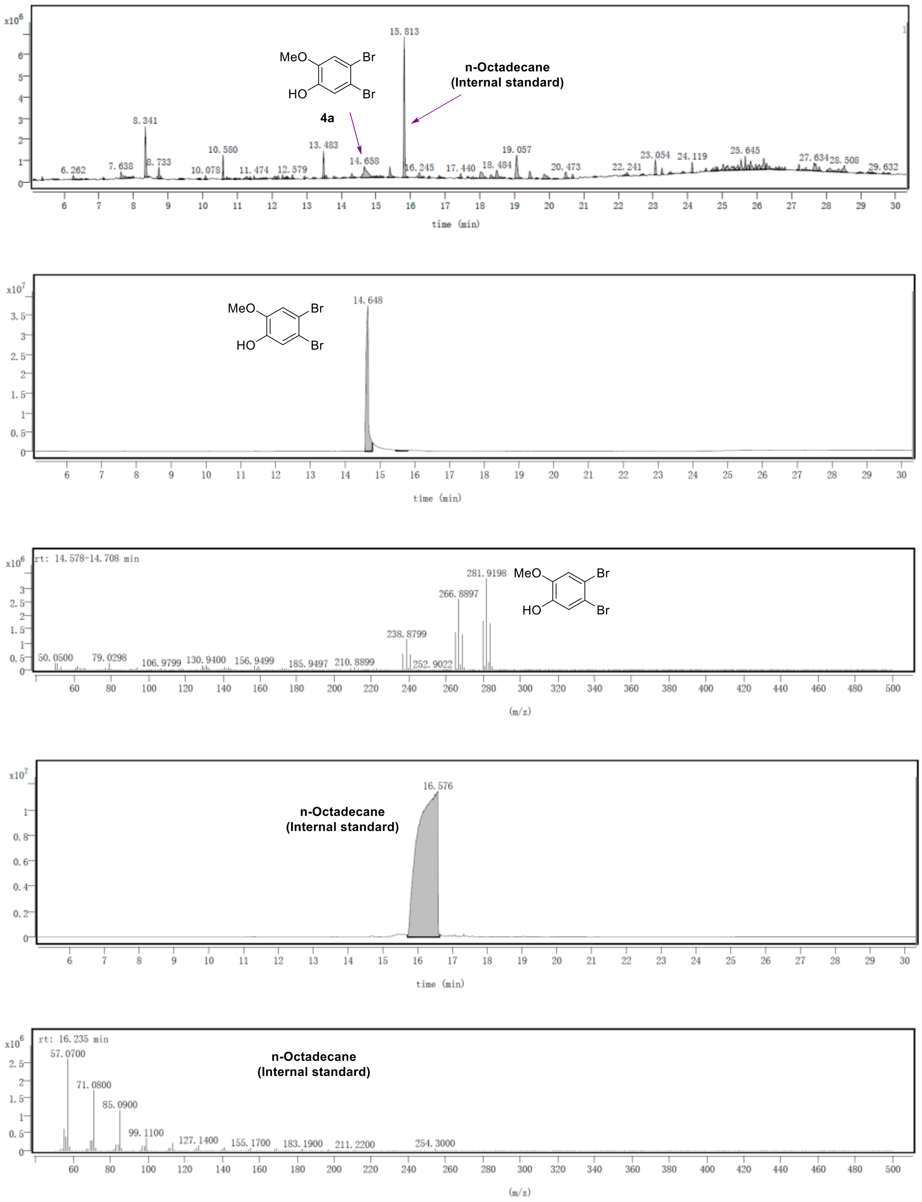

Supplementary Figure 9. Identification and quantification of monomer product **4a** obtained by bromination and depolymerization of larch lignin.

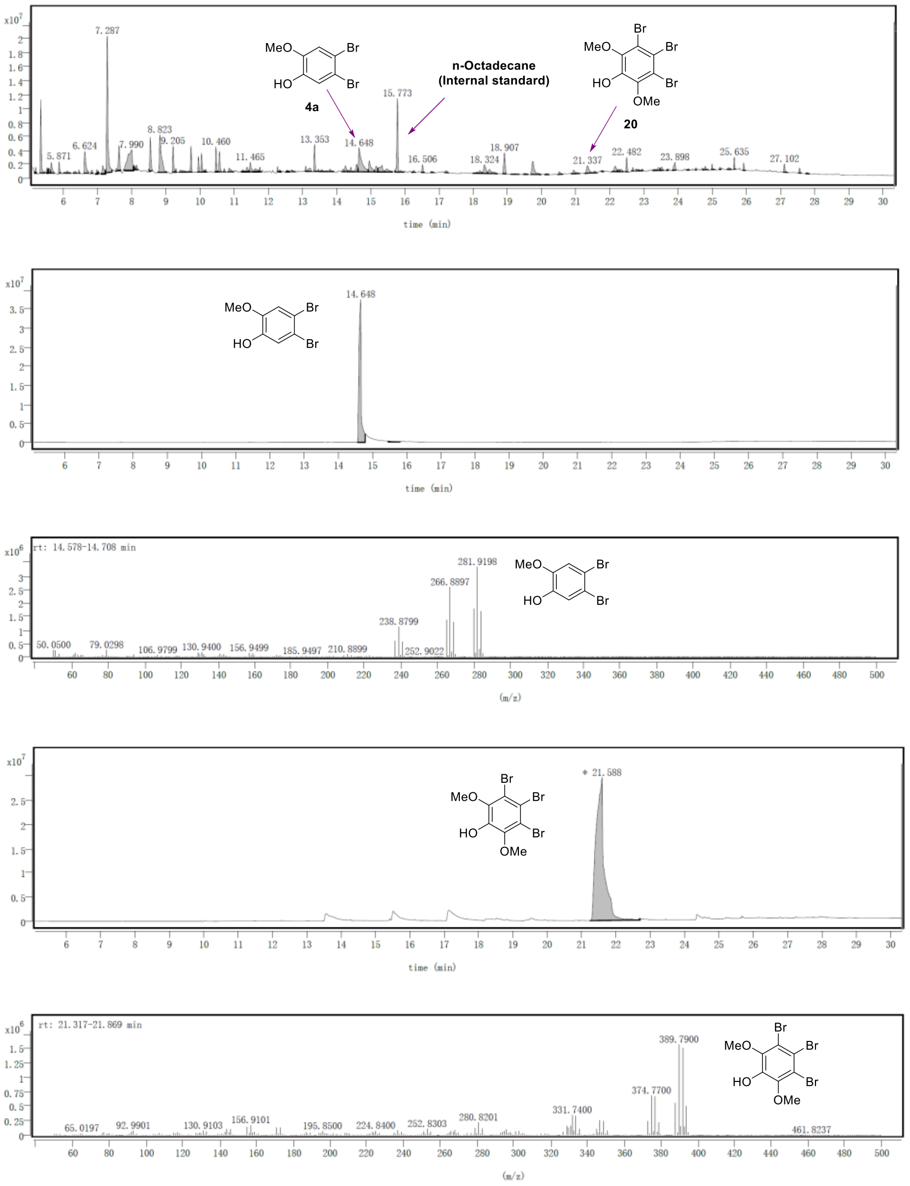

Supplementary Figure 10. Identification and quantification of monomer products **4a** and **20** obtained by bromination and depolymerization of birch lignin.

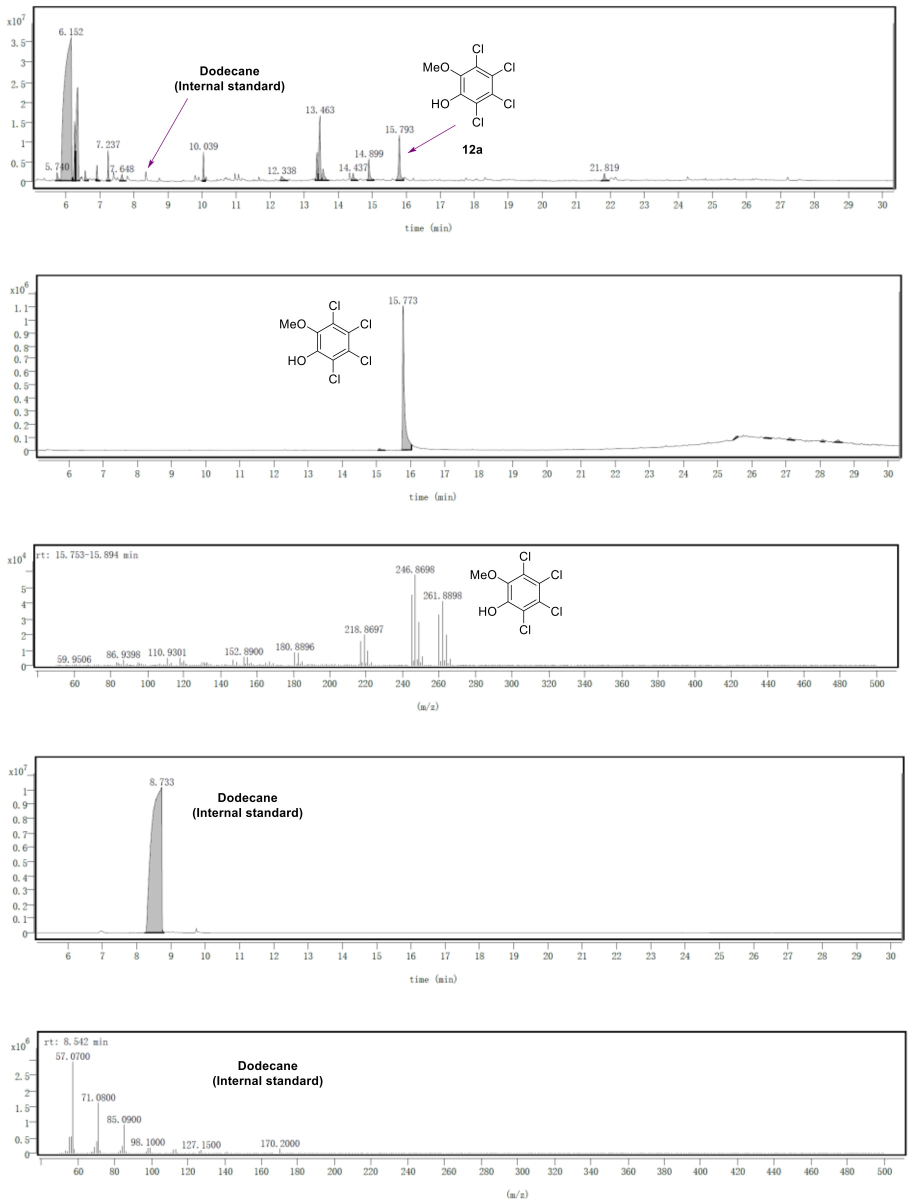

Supplementary Figure 11. Identification and quantification of monomer product **12a** obtained by chlorination and depolymerization of larch lignin.

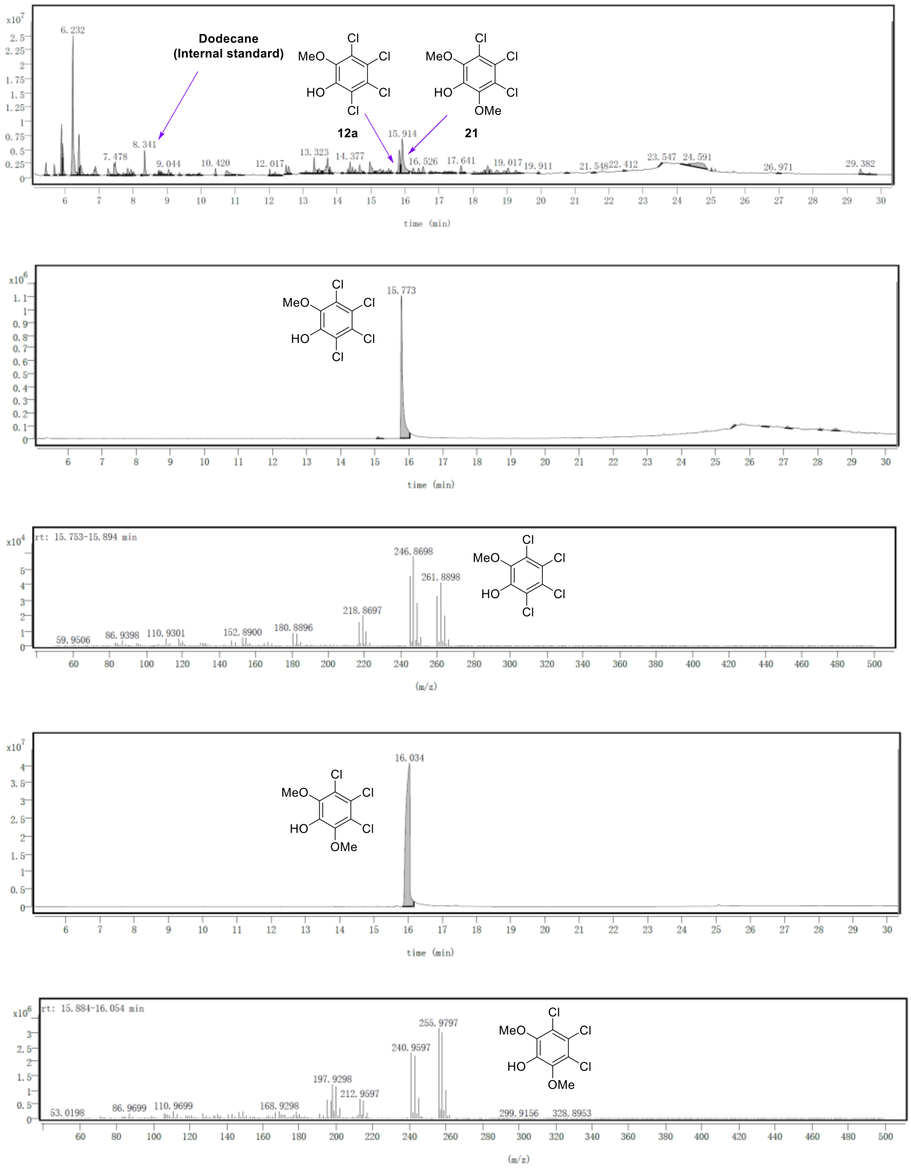

Supplementary Figure 12. Identification and quantification of monomer products **12a** and **21** obtained by chlorination and depolymerization of birch lignin.

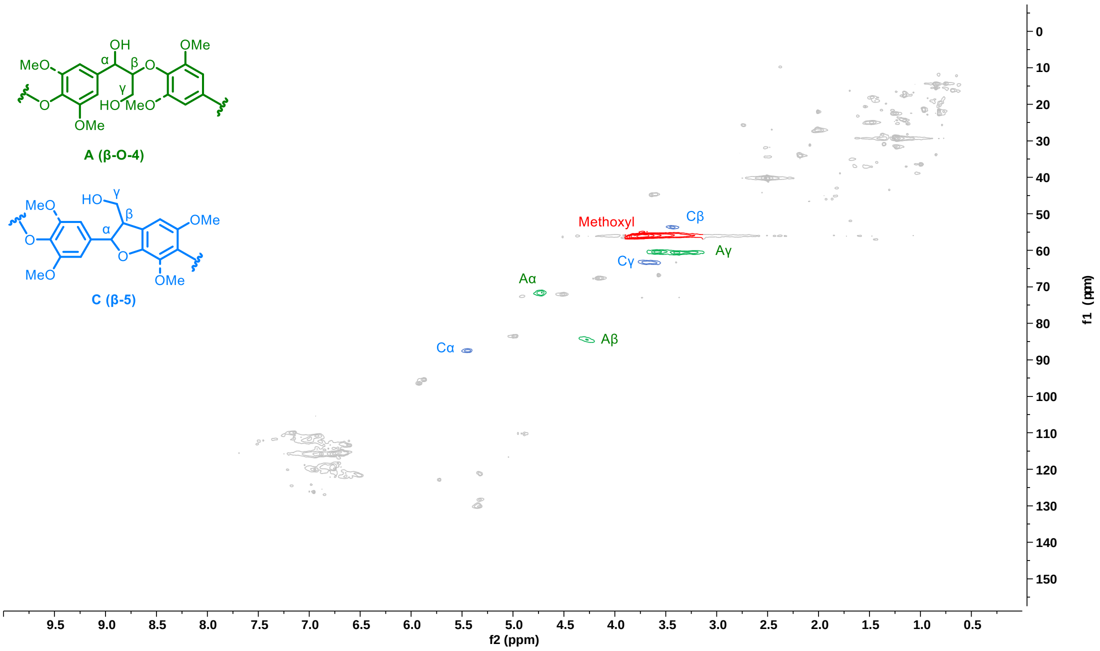

Supplementary Figure 13. 2D HSQC spectrum for native larch lignin (in DMSO-d_6).

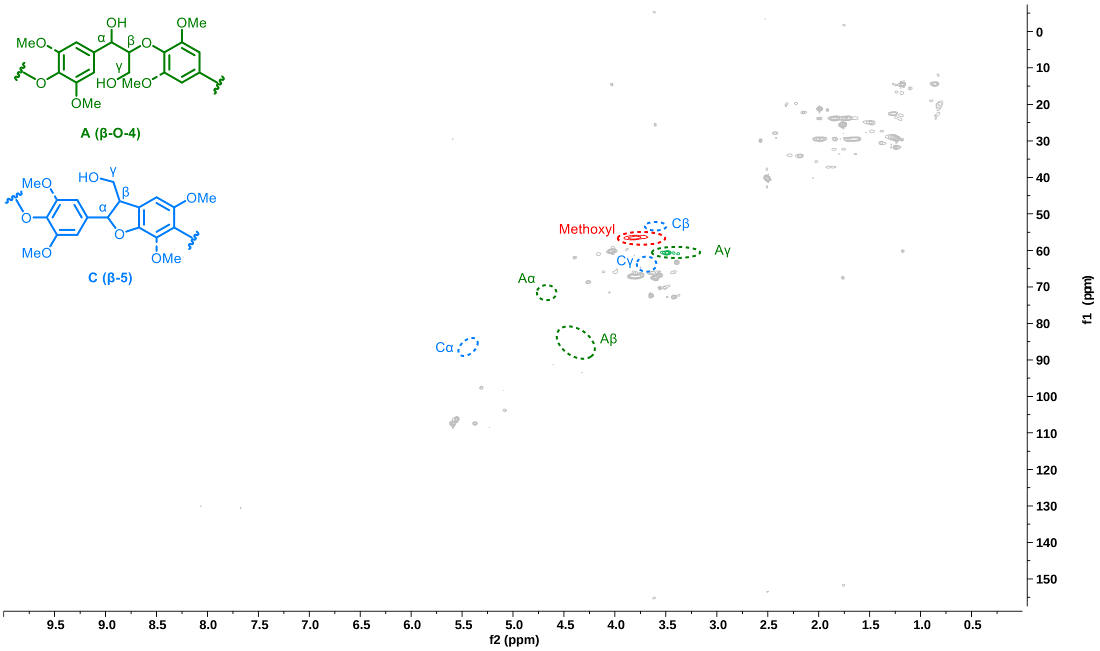

Supplementary Figure 14. 2D HSQC spectrum after depolymerization and bromination of larch lignin (in DMSO-d_6).

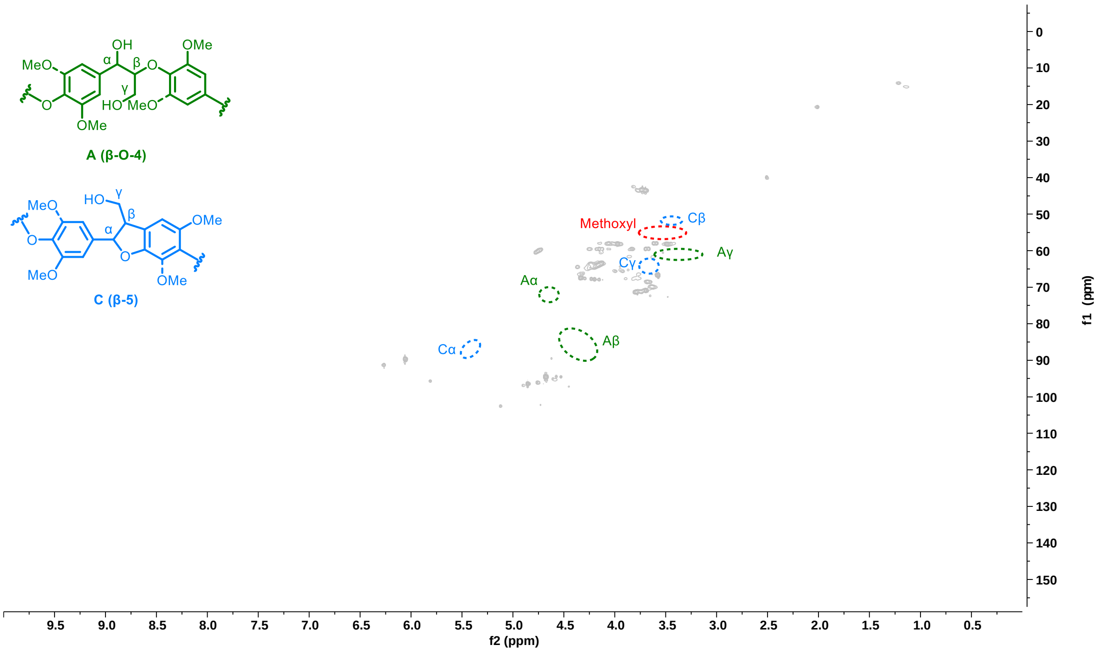

Supplementary Figure 15. 2D HSQC spectrum after depolymerization and chlorination of larch lignin (in DMSO-d6).

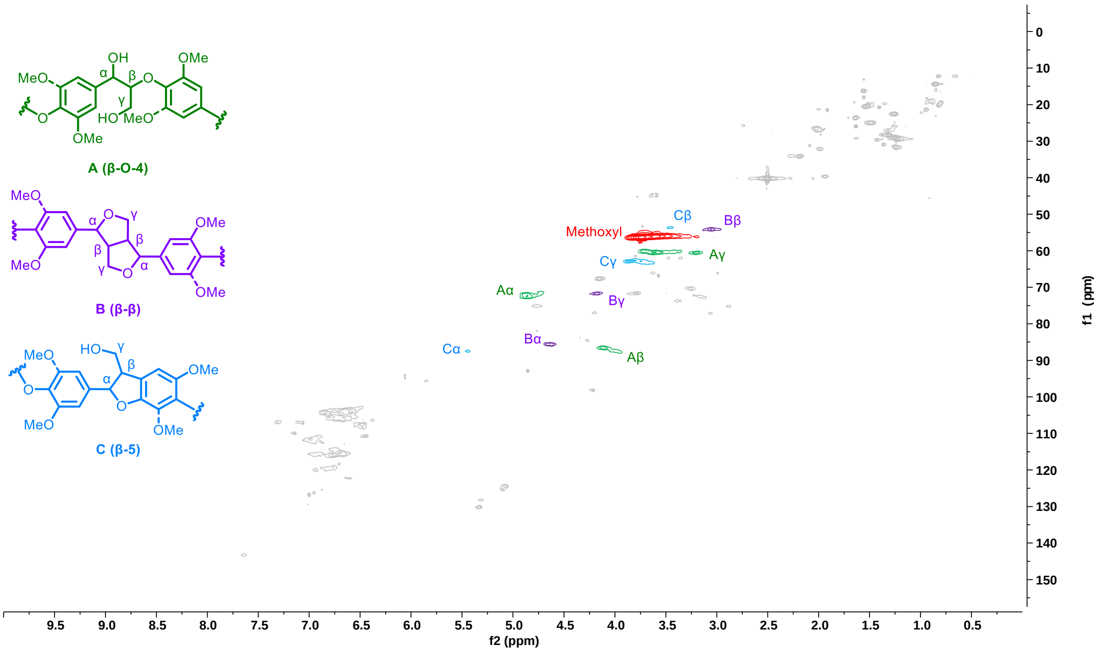

Supplementary Figure 16. 2D HSQC spectrum for native birch lignin (in DMSO-d6).

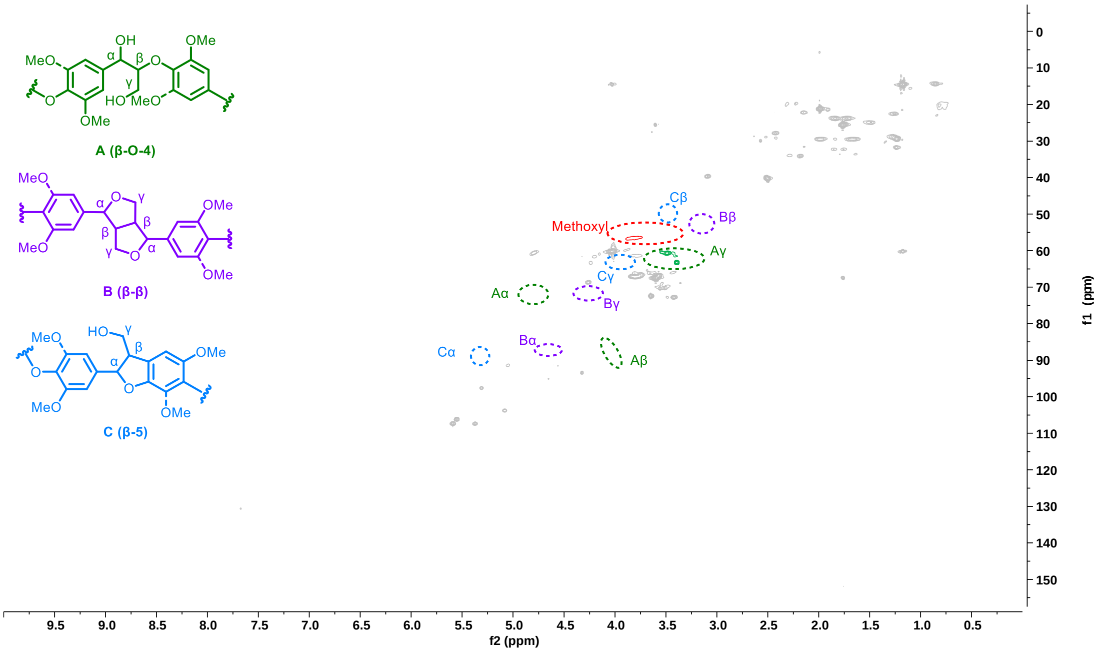

Supplementary Figure 17. 2D HSQC spectrum after depolymerization and bromination of birch lignin (in DMSO-d_6).

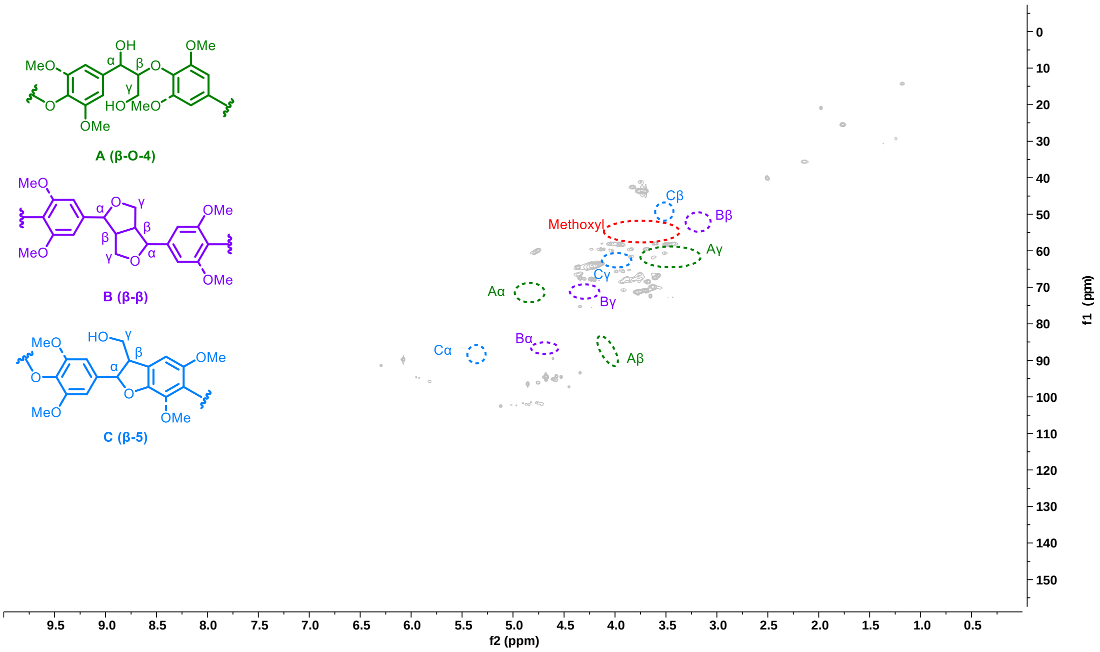

Supplementary Figure 18. 2D HSQC spectrum after depolymerization and chlorination of birch lignin (in DMSO-d_6).

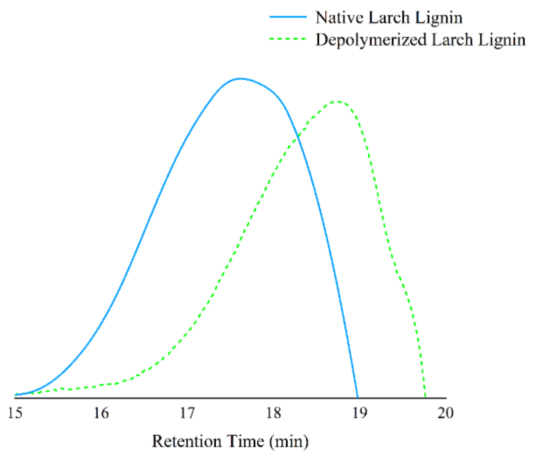

Supplementary Figure 19. GPC analysis of larch lignin before and after depolymerization and bromination.

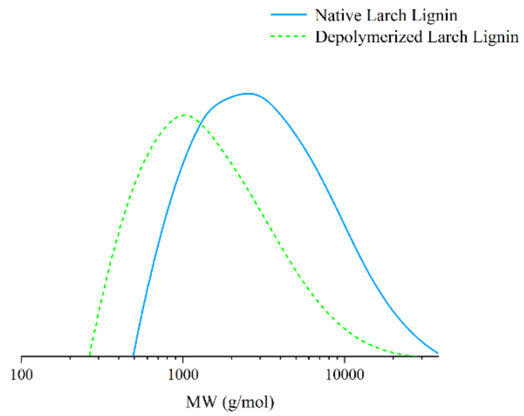

Supplementary Figure 20. GPC analysis of larch lignin before (blue, M_w = 2109, M_n = 896, PDI = 2.35) and after depolymerization and bromination (green, M_w = 911, M_n = 314, PDI =2.90).

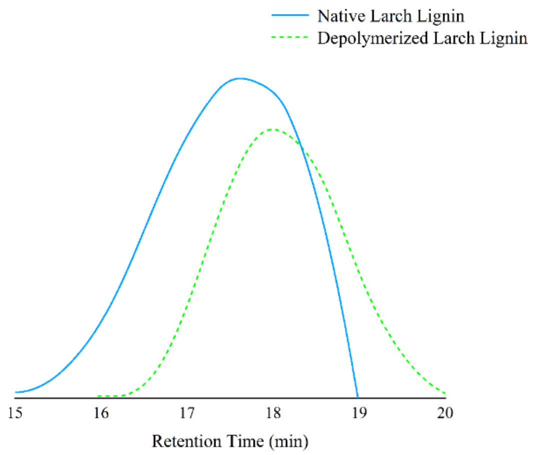

Supplementary Figure 21. GPC analysis of larch lignin before and after depolymerization and chlorination.

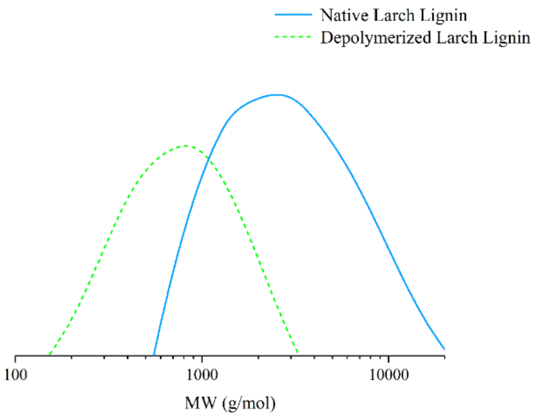

Supplementary Figure 22. GPC analysis of larch lignin before (blue, M_w = 2109, M_n = 896, PDI = 2.35) and after depolymerization and chlorination (green, M_w = 817, M_n = 420, PDI = 1.94).

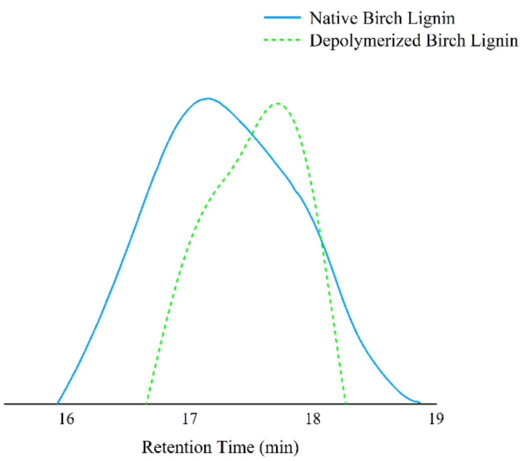

Supplementary Figure 23. GPC analysis of birch lignin before and after depolymerization and bromination.

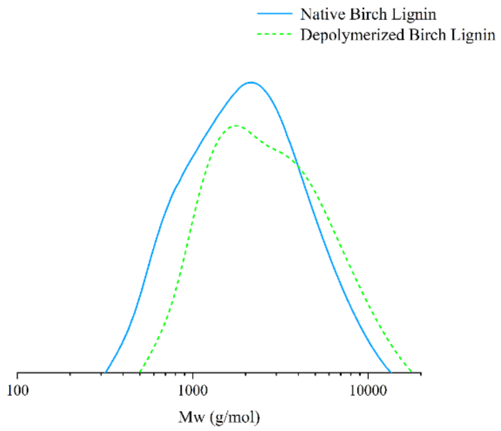

Supplementary Figure 24. GPC analysis of birch lignin before (blue, M_w = 2165, M_n = 1088, PDI = 1.98) and after depolymerization and bromination (green, M_w = 1760, M_n = 908, PDI = 1.93).

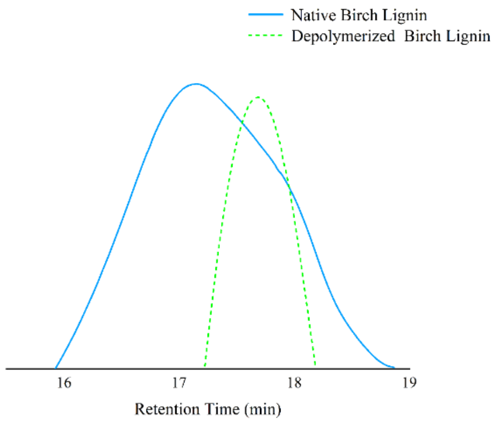

Supplementary Figure 25. GPC analysis of birch lignin before and after depolymerization and chlorination.

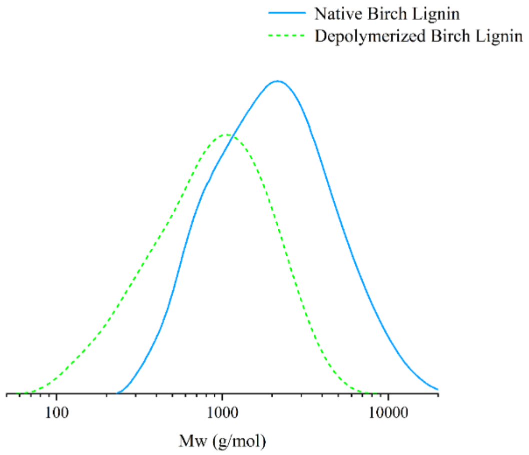

Supplementary Figure 26. GPC analysis of birch lignin before (blue, M_w = 2165, M_n = 1088, PDI = 1.98) and after depolymerization and chlorination (green, M_w = 1064, M_n = 538, PDI = 1.58).

Comparing to the native lignin, GPC measurements of the soluble fraction revealed a notable decrease in the molecular weight of the fragmented lignin.

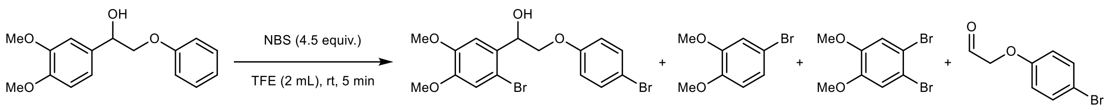

1b, 0.2 mmol | **1b'**, 2% yield, ^{1}H NMR, ^{13}C NMR detected | **14**, < 2% yield, GC-MS detected | **2a**, < 3% yield, GC-MS detected | **3b**, < 3% yield, GC-MS detected

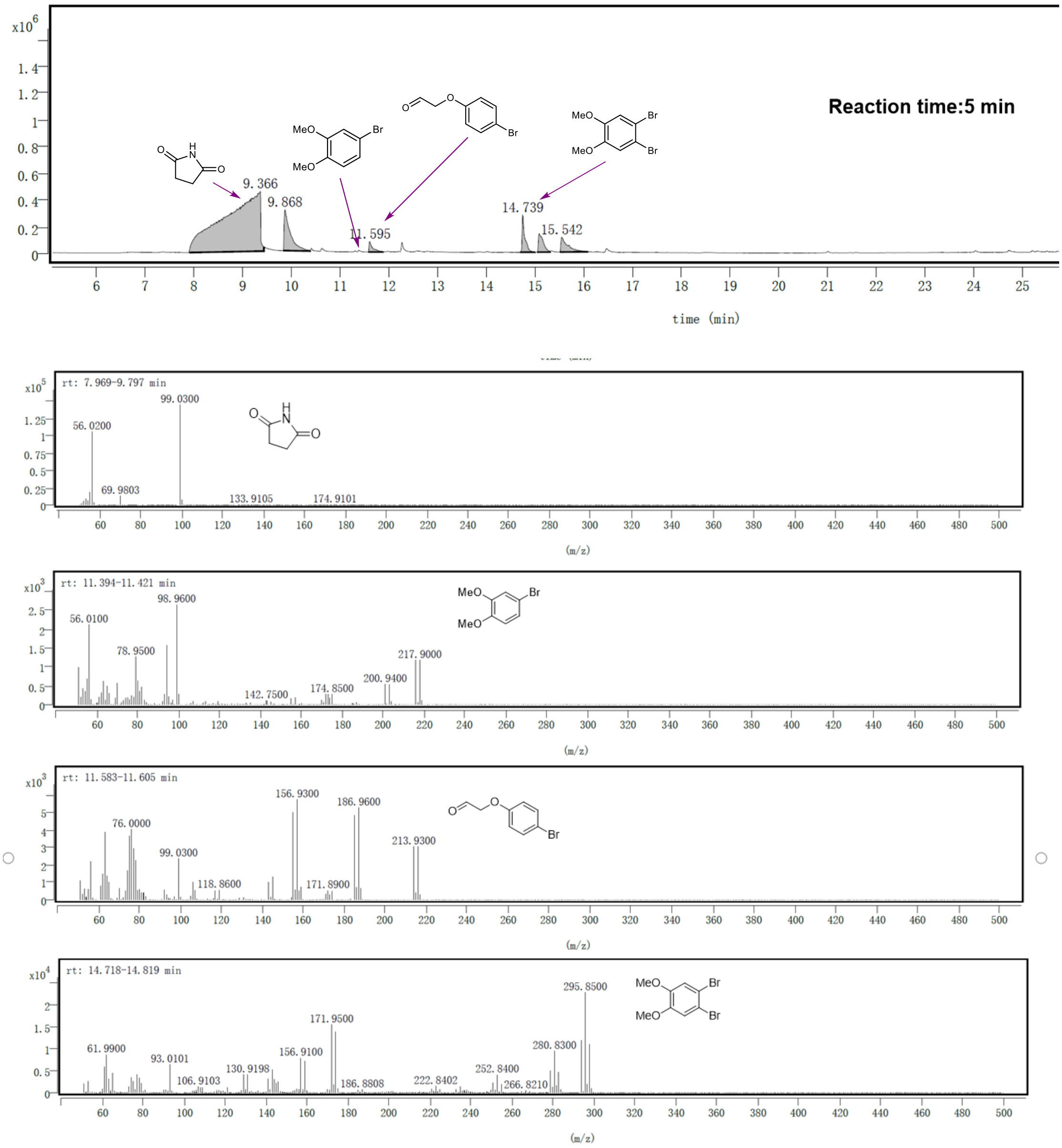

Supplementary Figure 27. GC-MS analysis for reaction of **1b** in 5 min.

^{1}H and ^{13}C NMR spectra proved the formation of product **1b'** via direct bromination of **1b**, and the GC-MS detected the formation of **14**. This result indicates that the polyhalogenation and the carbon-carbon bond cleavage occurred simultaneously.

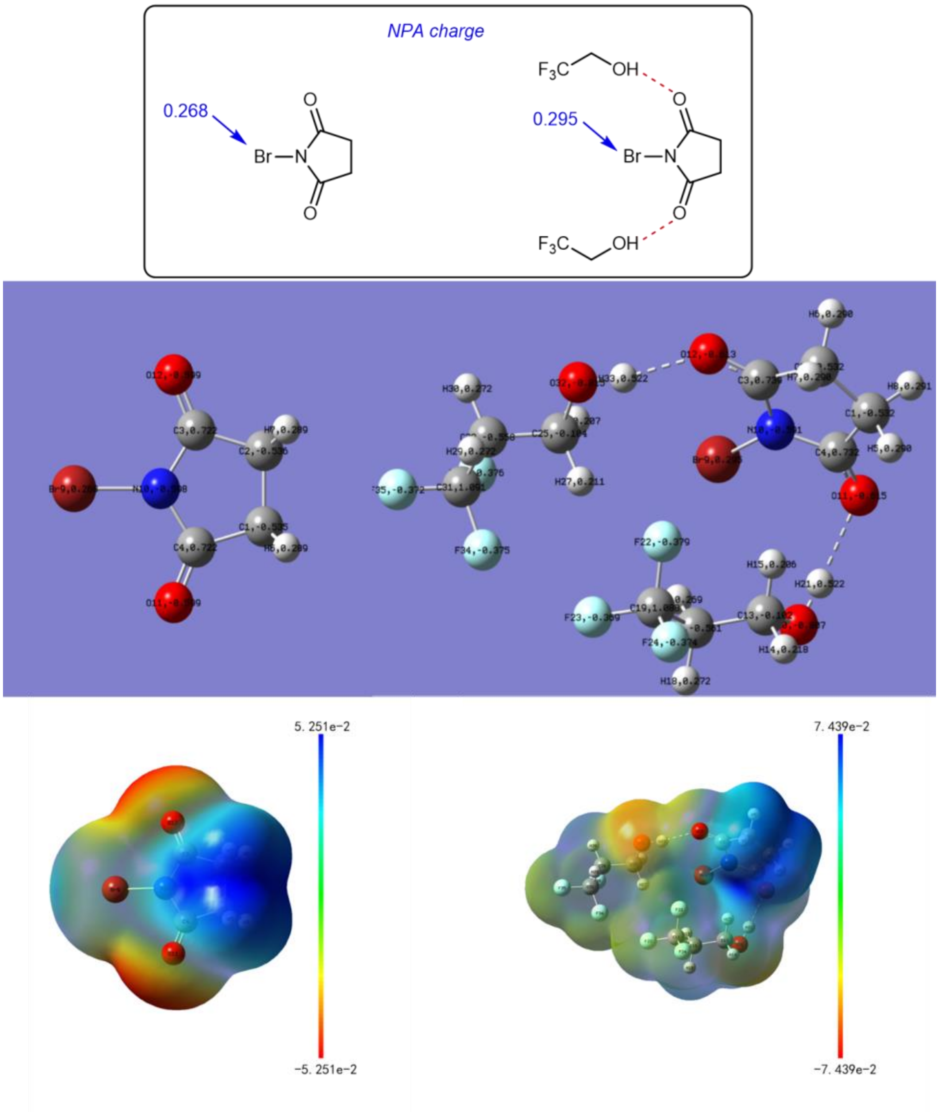

Supplementary Figure 28. Electron density calculation for GC-MS analysis for reaction of **1b** in 5 min.

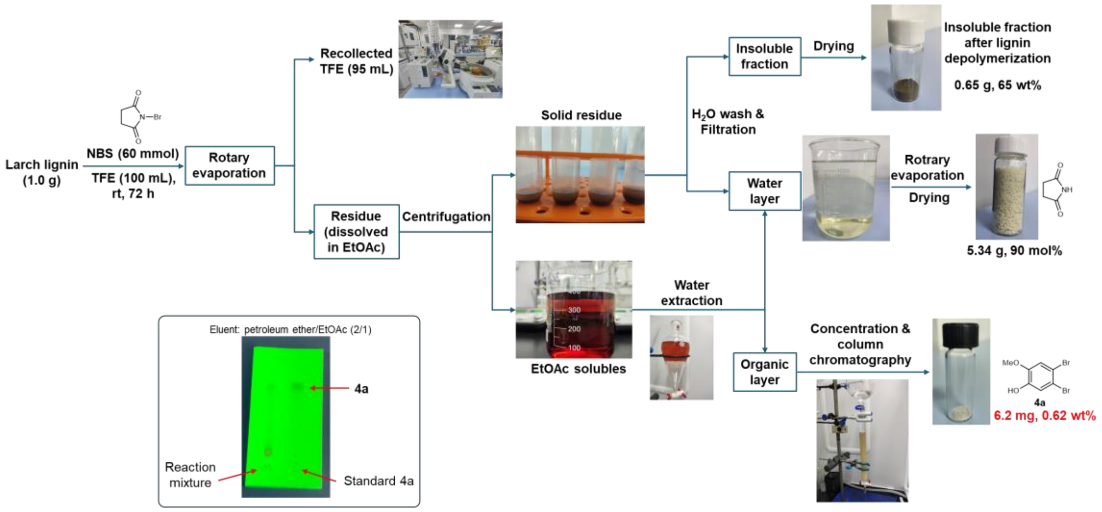

Supplementary Figure 29. Gramm scale experiment for depolymerization and bromination of larch lignin.

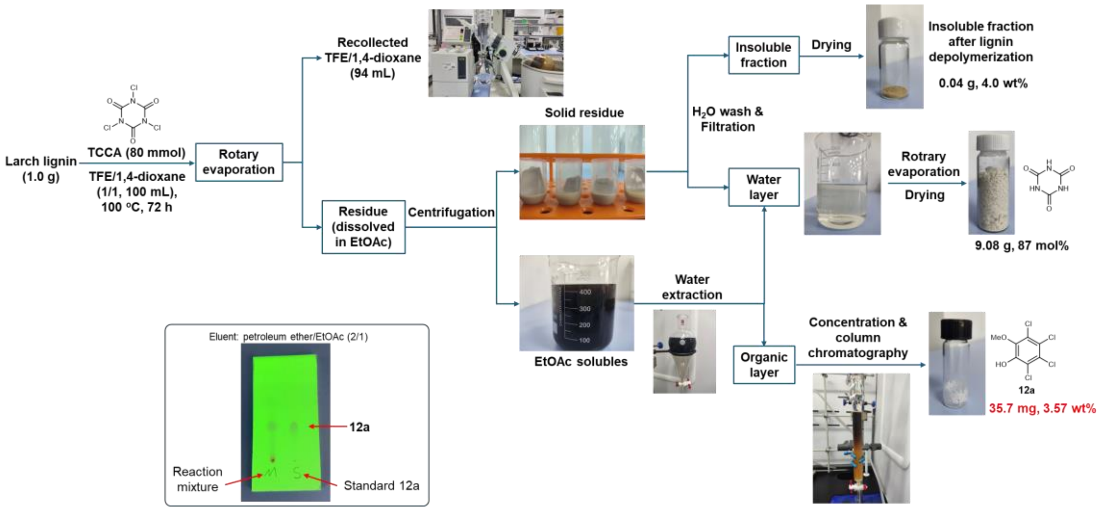

Supplementary Figure 30. Gramm scale experiment for depolymerization and chlorination of larch lignin.

Supplementary Methods

General Information

All commercially available compounds were purchased from Tansoole, TCI, Bidepharm or Adamas, and used without further purification, unless otherwise noted. TFE was purchased from Bidepharm. Reactions were monitored by thin-layer chromatography (TLC) carried out on 25 mm silica gel plates. Flash column chromatography was performed on silica gel (particle size 230-400 mesh, purchased from Tansoole) and eluted with petroleum ether/ethyl acetate. Nuclear magnetic resonance (NMR) spectra were recorded on Bruker-500 MHz spectrometer. ^{1}H and ^{13}C NMR chemical shifts (δ) are given in parts per million relative to $CDCl_3$ (7.26 ppm for ^{1}H) or TMS (0 ppm for ^{1}H), $CDCl_3$ (77.0 ppm for ^{13}C). ^{1}H multiplicities are reported as follows: singlet (s), doublet (d), triplet (t), doublet of doublets (dd), quartet (q), multiplet (m), and broad resonance (br). Heteronuclear single quantum coherence spectroscopy (HSQC) was recorded on a Bruker 500 spectrometer. High resolution mass (HRMS) spectral data were obtained on Agilent Technologies 6224 TOF LC MS spectrometer in ESI mode. IR spectra were recorded on a Perkin Elmer Paragon 1000 spectrometer and are reported in terms of frequency of absorption (cm^{-1}). Gas chromatography-mass spectrometry (GC-MS) data were recorded using an Agilent 8890-5977 (G3540A) instrument with helium as the mobile phase at an initial temperature of 60 °C for 1 min, then increased to 200 °C at a rate of 10 °C/min for 5 min and then raised to 280 °C at a rate of 15 °C/min for 5 min. Gel permeation chromatography (GPC) characterization of native lignin as well as fragmented lignin was performed using tetrahydrofuran as the mobile phase using an Agilent Cirrus GPC instrument with a UV diode array detector, and a refractive index detector.

Preparation of lignin model compounds

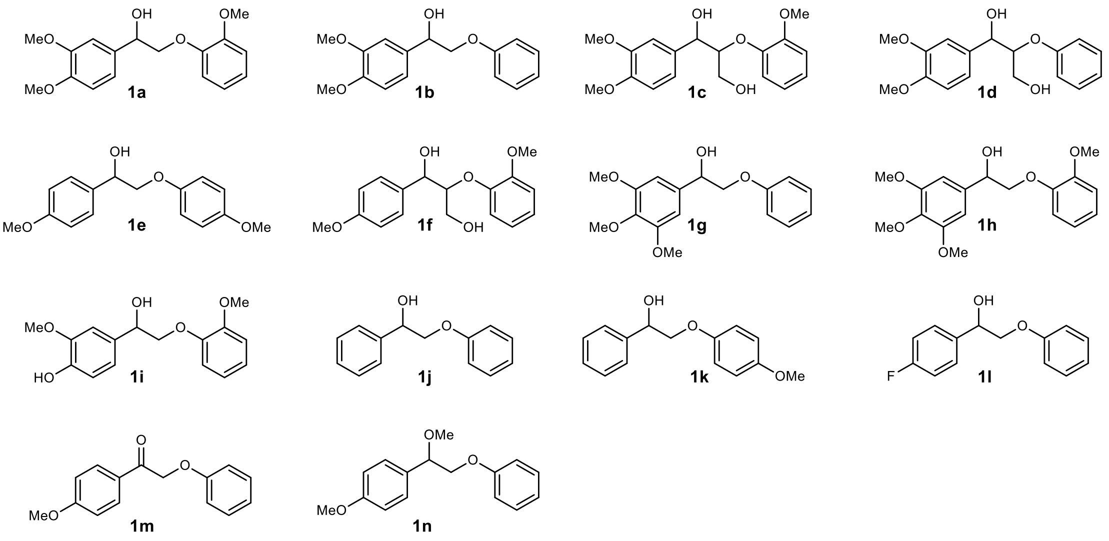

Lignin models **1a-m** were synthesized and used in our laboratory[1].

General procedure for synthesis of lignin models

Synthesis of lignin models **1a, 1b, 1e, 1g, 1h, 1j, 1k,** and **1n**.

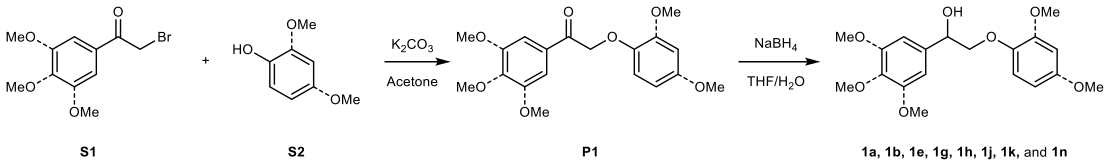

Compound **S1** (0.1 mol, 1.0 equiv.) was added to a stirred solution containing K_2CO_3 (0.15 mol, 1.5 equiv.), compound **S2** (0.2 mol, 2.0 equiv.) and acetone (300 mL), then stirred at rt for 12 h. After filtration, the filtrate was concentrated under the reduced pressure and the crude mixture was purified via flash column chromatography on silica with hexane / ethyl acetate (3:1) to afford the compound **P1**.

The resulting compound **P1** (0.08 mol, 1.0 equiv.) was dissolved in the mixed solvents of THF and H_2O (4:1, 350 mL), then portion-wisely added with sodium borohydride (0.16 mol, 2.0 equiv.). After stirring at rt for 12 h, the mixture was quenched with saturated aqueous NH_4Cl (50 mL) and diluted with 30 mL water. The aqueous portion was extracted with ethyl acetate (3 × 50 mL). The combined organic layers were dried over $MgSO_4$, filtered and concentrated under vacuum. The products were purified by flash silica chromatography with hexane / EtOAc (1:1) to afford the compound **1a, 1b, 1e, 1g, 1h, 1j, 1k,** and **1n**.

Synthesis of lignin models **1c, 1d,** and **1f**.

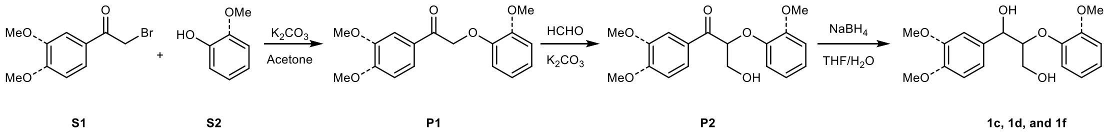

Compound **S1** (0.1 mol, 1.0 equiv.) was added to a stirred solution containing K_2CO_3 (0.15 mol, 1.5 equiv.), compound **S2** (0.2 mol, 2.0 equiv.) and acetone (300 mL), then stirred at rt for 12 h. After filtration, the filtrate was concentrated under the reduced pressure and the crude mixture was purified via flash column chromatography on silica with hexane / ethyl acetate (3:1) to afford the compound **P1**.

A solution containing compound **P1** (0.03 mol, 1.0 equiv.), K_2CO_3 (0.06 mol, 2.0 equiv.) and DMSO (150 mL) was stirred at rt for 30 min, then added with formaldehyde (37%) (0.06 mol, 2.0 equiv.). The resulting solution was stirred for 3 h, then added with 2 M NaOH (75 mL) and stirred for 1 h. After adding 1 M HCl to adjust the pH to 4, the solution was extracted with ethyl acetate. The extracted layers were dried and concentrated in vacuo to yield a residue, which was washed with ether to yield the compound **P2**.

The resulting compound **P2** (0.02 mol, 1.0 equiv.) was dissolved in the mixed solvents of THF and H_2O (4:1, 150 mL), then portion-wisely added with sodium borohydride (0.04 mol, 2.0 equiv.). After stirring at rt for 12 h, the mixture was quenched with saturated aqueous NH_4Cl (50 mL) and diluted with 30 mL water. The aqueous portion was extracted with ethyl acetate (3 × 50 mL). The combined organic layers were dried over $MgSO_4$, filtered and concentrated under vacuum. The residue was purified by column chromatography on silica gel (300-400 mesh) with a gradient eluent of petroleum ether/ethyl acetate (1:1) to afford the compounds **1c, 1d,** and **1f**.

Synthesis of lignin model **1m**.

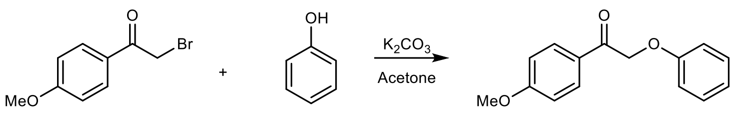

2-bromo-1-(4-methoxyphenyl)ethan-1-one   phenol   **1m**

2-Bromo-1-(4-methoxyphenyl)ethan-1-one (0.1 mol, 1.0 equiv.) was added to a stirred solution containing K_2CO_3 (0.15 mol, 1.5 equiv.), phenol (0.2 mol, 2.0 equiv.) and acetone (600 mL), then stirred at rt for 12 h. After filtration, the filtrate was concentrated under the reduced pressure and the crude mixture was purified by column chromatography on silica gel (300-400 mesh) with a gradient eluent of petroleum ether/ethyl acetate (3:1) to afford **1m** as yellow powder (20.1 g, 83% yield).

General procedure for depolymerization and halogenation of lignin models

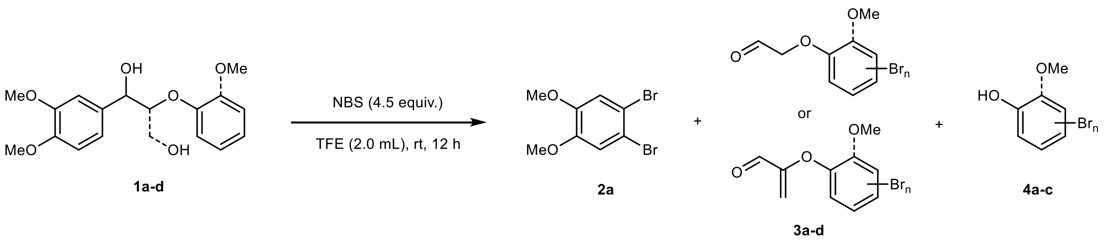

General procedure 1: A 10 mL sealed tube was charged with lignin model compound **1a-d** (0.2 mmol, 1.0 equiv.), N-bromosuccinimide (NBS) (0.9 mmol, 4.5 equiv.) and 2,2,2-Trifluoroethanol (TFE) (2 mL) under air atmosphere. The reaction was stirred for 12 h at room temperature. After the reaction was finished, the mixture was filtered through a silica gel pad (100-200 mesh) and the solvent was removed under vacuum. The residue was purified by column chromatography on silica gel (300-400 mesh) with a gradient eluent of petroleum ether/ethyl acetate (10:1 to 5:1) to yield the desired product.

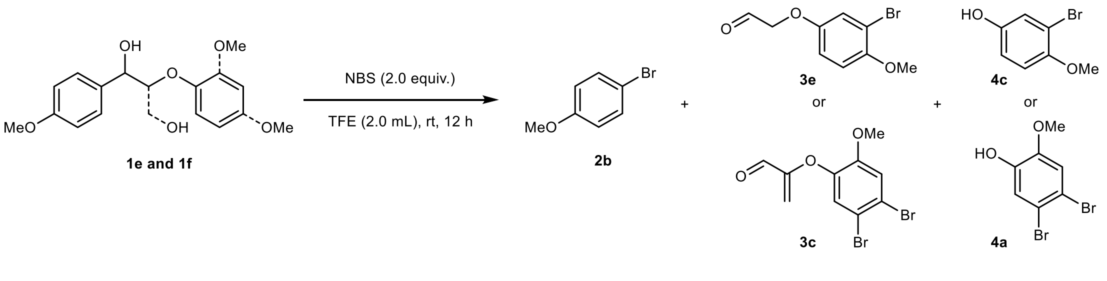

General procedure 2: A 10 mL sealed tube was charged with lignin model compound **1e-f** (0.2 mmol, 1.0 equiv.), NBS (0.4 mmol, 2.0 equiv.) and TFE (2 mL) under air atmosphere. The reaction was stirred for 12 h at room temperature. After the reaction was finished, the mixture was filtered through a silica gel pad (100-200 mesh) and the solvent was removed under vacuum. The residue was purified by column chromatography on silica gel (300-400 mesh) with a gradient eluent of petroleum ether/ethyl acetate (10:1 to 5:1) to yield the desired product.

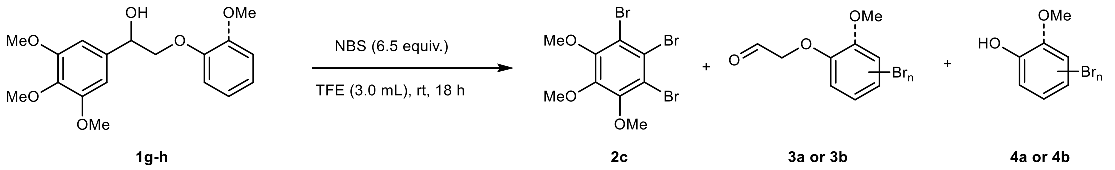

General procedure 3: A 10 mL sealed tube was charged with lignin model compound **1g-h** (0.2 mmol, 1.0 equiv.), NBS (1.3 mmol, 6.5 equiv.) and TFE (3 mL) under air atmosphere. The reaction was stirred for 18 h at room temperature. After the reaction was finished, the mixture was filtered through a silica gel pad (100-200 mesh) and the solvent was removed under vacuum. The residue was purified by column chromatography on silica gel (300-400 mesh) with a gradient eluent of petroleum ether/ethyl acetate (10:1 to 5:1) to yield the desired product.

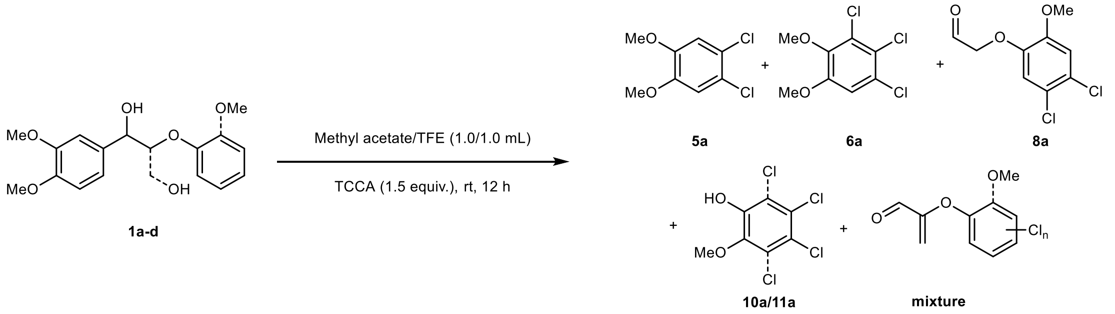

General procedure 4: A 10 mL sealed tube was charged with lignin model compound **1a-d** (0.2 mmol, 1.0 equiv.), trichloroisocyanuric acid (TCCA) (0.3 mmol, 1.5 equiv.) and methyl acetate/TFE (1/1, 2 mL) under air atmosphere. The reaction was stirred for 12 h at room temperature. After the reaction was finished, the mixture was filtered through a silica gel pad (100-200 mesh) and the solvent was removed

under vacuum. The residue was purified by column chromatography on silica gel (300-400 mesh) with a gradient eluent of petroleum ether/ethyl acetate (10:1 to 5:1) to yield the desired product.

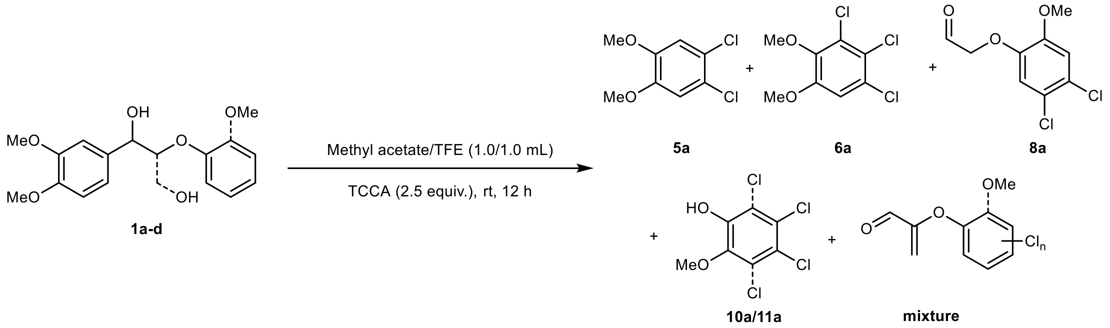

General procedure 5: A 10 mL sealed tube was charged with lignin model compound **1a-d** (0.2 mmol, 1.0 equiv.), TCCA (0.5 mmol, 2.5 equiv.) and methyl acetate/TFE (1/1, 2 mL) under air atmosphere. The reaction was stirred for 12 h at room temperature. After the reaction was complete, the mixture was filtered through a silica gel pad (100-200 mesh) and the solvent was removed under vacuum. The residue was purified by column chromatography on silica gel (300-400 mesh) with a gradient eluent of petroleum ether/ethyl acetate (10:1 to 5:1) to yield the desired product.

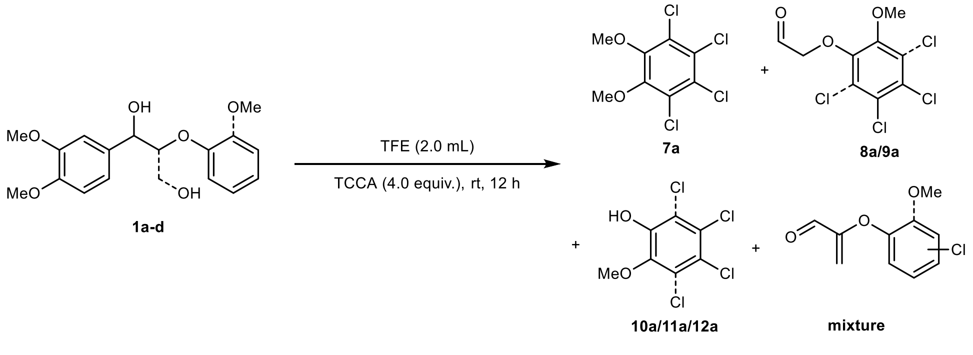

General procedure 6: A 10 mL sealed tube was charged with lignin model compound **1a-d** (0.2 mmol, 1.0 equiv.), TCCA (0.8 mmol, 4.0 equiv.) and TFE (20 mL) under air atmosphere. The reaction was stirred for 12 h at room temperature. After the reaction was complete, the mixture was filtered through a silica gel pad

(100-200 mesh) and the solvent was removed under vacuum. The residue was purified by column chromatography on silica gel (300-400 mesh) with a gradient eluent of petroleum ether/ethyl acetate (10:1 to 5:1) to yield the desired product.

Large-scale reaction

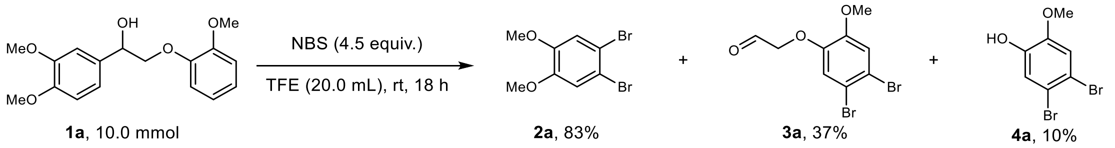

A 50 mL sealed tube was charged with lignin model compound **1a** (3.04 g, 10.0 mmol, 1.0 equiv.), NBS (8.01 g, 45.0 mmol, 4.5 equiv.) and TFE (20 mL) under air atmosphere. The reaction was stirred for 18 h at room temperature. After the reaction was completed, the mixture was filtered through a thin pad of silica gel (100-200 mesh) and the solvent was removed under vacuum. The residue was purified by column chromatography on silica gel (300-400 mesh) with a gradient eluent of petroleum ether/ethyl acetate (10:1 to 5:1) to yield **2a** (2.45 g, 83% yield), **3a** (1.19 g, 37% yield), and **4a** (0.28 g, 10% yield).

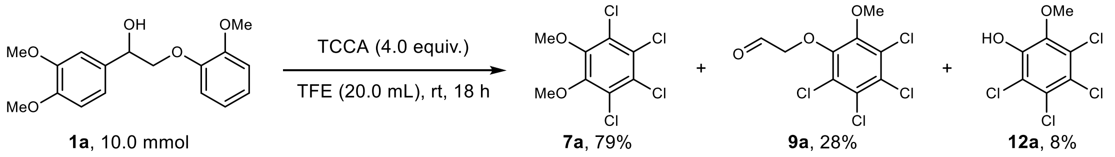

A 50 mL sealed tube was charged with lignin model compound **1a** (3.04 g, 10.0 mmol, 1.0 equiv.), TCCA (9.29 g, 40.0 mmol, 4.0 equiv.) and TFE (20 mL) under air atmosphere. The reaction was stirred for 18 h at room temperature. After the reaction was completed, the mixture was filtered through a thin pad of silica gel (100-200 mesh) and the solvent was removed under vacuum. The residue was purified by column chromatography on silica gel (300-400 mesh) with a gradient eluent of petroleum ether/ethyl acetate (10:1 to 5:1) to yield **7a** (2.17 g, 79% yield), **8a** (0.85 g, 28% yield), and **12a** (0.21 g, 8% yield).

Native lignin extraction

The dioxane-lignin was extracted using a similar method described previously. 100 g larch saw dust, 720 mL 1,4-dioxane and 10 mL 2M HCl were introduced in a round bottom flask. The flask was put in an oil bath and heated at reflux (110 °C) for 3 h under N_2. The mixture was then cooled to room temperature and the lignin containing mixture was collected by filtration. The collected mixture was partially concentrated in vacuo to give a gummy residue which was taken up in 100 mL of acetone/water (9/1, v/v) and precipitated by addition to rapidly stirring water (2.0 L). The crude lignin was collected by filtration and dried under vacuum. The dried crude lignin was taken up in 100 mL of acetone/methanol (9/1, v/v) and precipitated by dropwise addition to rapidly stirring hexane (2.0 L). The precipitated lignin was collected by filtration and dried under vacuum to give a purified larch lignin-dioxane (5.7 g). This lignin was used in subsequent experiments without further processing. Subsequently, lignin of different biomasses was extracted by the same method. The extraction rates of different lignin samples are shown in Supplementary Table 5.

General procedure for depolymerization and halogenation of native lignin

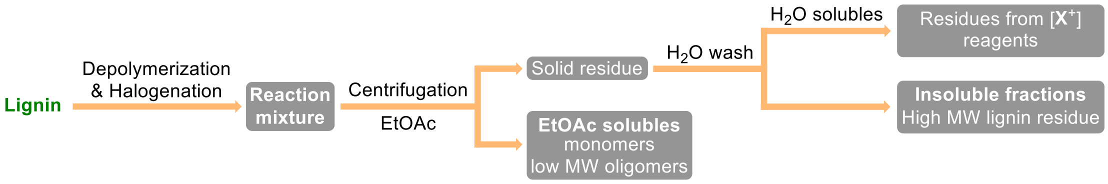

General procedure for depolymerization and bromination of lignin. A 25 mL sealed tube was charged with extracted native lignin (100.0 mg), NBS (6.0 mmol, 1.0678 g) and TFE (10 mL) under air atmosphere. The reaction was stirred for 72 h at room temperature. After the reaction is complete, the mixture was centrifuged with ethyl acetate (20 mL × 3) to separate the solid and liquid phases. The solid-phase fraction was stirred in 100 mL of water for 3 h to remove residues from the bromination reagent, and the insoluble fraction was collected by Brinell funnel filtration before drying and weighing. The liquid phase was concentrated in a vacuum and diluted with 30 mL of ethyl acetate, then extracted with water (50 mL × 3) to remove bromination reagent residues from the solution. The organic phase was

collected and dried with anhydrous sodium sulfate, then concentrated in a vacuum to quantify the product by GC-MS.

General procedure for depolymerization and chlorination of lignin. A 25 mL sealed tube was charged with extracted native lignin (100.0 mg), TCCA (8.0 mmol, 1.8592 g) and TFE/1,4-dioxane (1/1, 10 mL) under air atmosphere. The reaction was stirred for 72 h at 100 ℃. After the reaction is complete, the mixture is centrifuged with ethyl acetate (20 mL × 3) to separate the solid and liquid phases. The solid-phase fraction was stirred in 100 mL of water for 3 h to remove residues from the chlorination reagent, and the insoluble fraction was collected by Brinell funnel filtration before drying and weighing. The liquid phase was concentrated in a vacuum and diluted with 30 mL of ethyl acetate, then extracted with water (50 mL × 3) to remove chlorination reagent residues from the solution. The organic phase was collected and dried with anhydrous sodium sulfate, then concentrated in a vacuum to quantify the product by GC-MS.

The solid and liquid phases were collected and the HSQC spectra of the residue was analyzed using DMSO-d_6 as the solvent and GPC analysis was performed. The comparison of HSQC spectra and GPC before and after lignin depolymerization is shown in Supplementary Figures 13-26.

Gram scale experiment of depolymerization and halogenation of larch lignin

Bromination: A 250 mL flask was charged with extracted larch lignin (1.0 g), NBS (60.0 mmol, 10.678 g), and TFE (100 mL) under air atmosphere. The reaction was stirred for 72 h at room temperature. After the reaction is complete, the TFE solvent was collected by a rotary evaporator. The residue was dissolved in ethyl acetate (200 mL) and centrifuged to separate the solid and liquid phases. The solid-phase fraction was stirred in 500 mL of water for 3 h to remove residues from the bromination reagent, and the insoluble fraction was collected by Brinell funnel filtration before drying and weighing (0.65 g, 65 wt%). The liquid phase was concentrated in a vacuum and diluted with 30.0 mL of ethyl acetate, then extracted with water (100 mL

× 3) to remove bromination reagent residues from the solution. Then combined the bromination reagent residues (in water) and concentrated by a rotary evaporator, followed by a drying process in the 100 °C oven to afford succinimide (5.34 g, 90 mol% yield). The organic layers were combined and evaporated. Purification of the residue by column chromatography on silica gel (300-400 mesh) with a gradient eluent of petroleum ether/ethyl acetate (2:1) gave 6.2 mg **4a** (0.62 wt% yield) as a white solid.

Chlorination: A 250 mL two necked flask was equipped with a reflux condenser and charged with extracted larch lignin (1.0 g), TCCA (80.0 mmol, 18.592 g), and TFE/1,4-dioxane (1/1, 100 mL) under air atmosphere. The reaction was stirred for 72 h at 100 °C. After the reaction is complete, the solvent was collected by a rotary evaporator. The residue was dissolved in ethyl acetate (200 mL) and centrifuged to separate the solid and liquid phases. The solid-phase fraction was stirred in 500 mL of water for 3 h to remove residues from the chlorination reagent, and the insoluble fraction was collected by Brinell funnel filtration before drying and weighing (0.04 g, 4.0 wt%). The liquid phase was concentrated in a vacuum and diluted with 30.0 mL of ethyl acetate, then extracted with water (100 mL × 3) to remove chlorination reagent residues from the solution. Then combined the chlorination reagent residues (in water) and concentrated by a rotary evaporator, followed by a drying process in the 100 °C oven to afford cyanuric acid (9.08 g, 87 mol% yield). The organic layers were combined and evaporated. Purification of the residue by column chromatography on silica gel (300-400 mesh) with a gradient eluent of petroleum ether/ethyl acetate (2:1) gave 35.7 mg **12a** (3.57 wt% yield) as a white solid.

Identification and quantification of monomeric products from lignin halogenation depolymerization

Products are characterized and validated by GC-MS retention time and accurate mass detected to conform to real standards purchased or synthesized (Supplementary Table 7).

After lignin depolymerization and halogenation, the monomer products were analyzed and quantified by GC-MS. Monomers **4a** and **20** were quantified with *n*-octadecane as internal standard (Supplementary Figures 9 and 10). Monomers **12a** and **21** were quantified with dodecane as internal standard (Supplementary Figures 11 and 12).

Synthetic application of aryl halides

A variety of aryl halides obtained from depolymerization and halogenation of lignin are useful precursors and components for chemical production, so the further diversified synthetic transformation of lignin-derived polybrominated or polychlorinated aromatics has been studied to demonstrate their practicability.

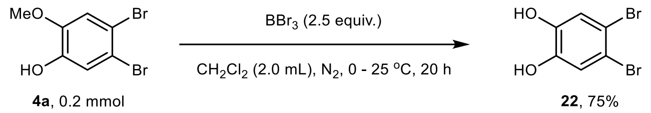

Boron tribromide (1 M in CH_2Cl_2, 0.5 mL, 0.5 mmol, 2.5 equiv.) was added slowly to a solution of 1,2-dibromo-4-hydroxy-5-methoxybenzene **4a** (56.38 mg, 0.2 mmol, 1.0 equiv.) in CH_2Cl_2 (2 mL), and the resulting reaction mixture was stirred at 0 °C under N_2 atmosphere for 1 hour and raised to room temperature for 20 h. The reaction was quenched by the addition of 0.1 M HCl and the resulting biphasic mixture was saturated with sodium chloride. The layers were separated and the aqueous phase was extracted with CH_2Cl_2. The combined organic layers were dried (Na_2SO_4) and concentrated in vacuo and the residue was purified by column chromatography on silica gel (300-400 mesh) with petroleum ether/ethyl acetate (3:1) to give 4,5-dibromobenzene-1,2-diol **22** (40.18 mg, 75% yield) as an off-white solid[2]. ^{1}H NMR (500 MHz, $CDCl_3$): δ 7.13 (s, 2H), 5.70 (s, 2H); ^{13}C NMR (126 MHz, $CDCl_3$): δ 143.67, 119.86, 114.74.

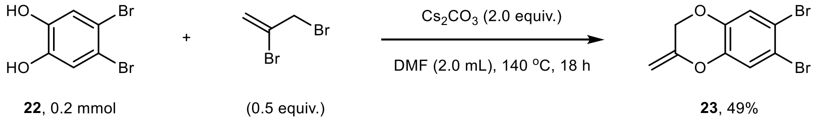

A 10 mL Schlenk tube was charged with 4,5-dibromobenzene-1,2-diol **22** (53.58 mg, 0.2 mmol, 1.0 equiv.), 2,3-dibromopropene (19.98 mg, 0.1 mmol, 0.5 equiv.), Cs_2CO_3 (130.32 mg, 0.4 mmol, 2.0 equiv.) and DMF (2.0 mL) under N_2. The reaction mixture was vigorously stirred for 18 h at 140 °C. After cooling to 100 °C, the reaction mixture was quenched with 2 M HCl (2.5 mL) and then allowed to cool to room temperature. The reaction mixture was diluted with H_2O (20 mL) and extracted with Et_2O (3 × 15 mL). The combined organic layers were washed with brine (3 × 10 mL), dried over $MgSO_4$, filtered and concentrated under reduced pressure. The product was purified by column chromatography on silica gel (300-400 mesh) with *n*-hexane /ethyl acetate (5:1) to afford the corresponding 6,7-dibromo-2-methylene-2,3-dihydrobenzo[b][1,4]dioxine **23** (29.98 mg, 49% yield) as a white solid. ^{1}H NMR (500 MHz, $CDCl_3$): δ 7.22 (s, 1H), 7.18 (s, 1H), 4.81 (d, *J* = 2.2 Hz, 1H), 4.48 (s, 2H), 4.43 (d, *J* = 2.2 Hz, 1H); ^{13}C NMR (126 MHz, $CDCl_3$): δ 148.81, 143.52, 142.34, 121.71, 120.89, 116.19, 116.12, 93.04, 64.57. IR (ATR) *ν* (cm^{-1}) =3089, 2998, 2921, 1676, 1576, 1477, 1459, 1370, 1292, 1237, 1168, 1096, 1019, 999, 973, 888, 862, 789, 719, 630, 580. HRMS: m/z (ESI-TOF) calculated for $C_9H_7Br_2O_2$ $[M+H]^+$: 306.8786, found 306.8784.

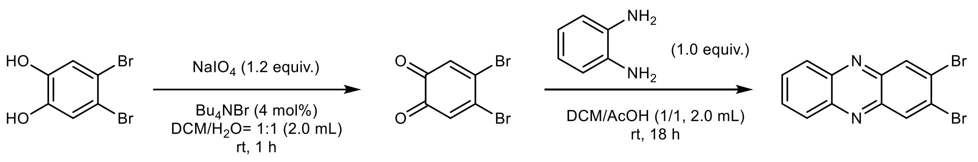

22, 0.2 mmol   4,5-Dibromocyclohexa-3,5-diene-1,2-dione   **24**, 56%

The compound 4,5-dibromobenzene-1,2-diol **22** (53.58 mg, 0.2 mmol, 1.0 equiv.) was dissolved in 2.0 mL of CH_2Cl_2. After adding sodium periodate (51.81 mg, 0.24 mmol, 1.2 equiv.) and (*n*-butyl)$_4$NBr (2.57 mg, 0.008 mmol, 4 mol%), dissolved in 2.0 mL of DI water, the reaction mixture turned red and was stirred for 1 h at room temperature. After extraction with CH_2Cl_2, the organic phase was used without further purification. The crude product 4,5-dibromocyclohex-3,5-dien-1,2-dione obtained in the first step was dissolved in 2 mL of CH_2Cl_2/acetic acid (1:1) with benzene-1,2-diamine (21.62 mg, 0.2 mmol, 1.0 equiv.), and the reaction mixture was stirred for 18 h at room

temperature. The crude residue was purified by column chromatography on silica gel (300-400 mesh) with *n*-hexane/ethyl acetate (3:1) to yield 2,3-dibromophenazine **24** (37.85 mg, 56% yield) as a yellow solid[3]. ^{1}H NMR (500 MHz, $CDCl_3$): δ 8.61 (s, 2H), 8.22 (dd, *J* = 6.7, 3.4 Hz, 2H), 7.88 (dd, *J* = 6.9, 3.4 Hz, 2H); ^{13}C NMR (126 MHz, $CDCl_3$): δ 142.91, 141.33, 132.39, 130.43, 128.77, 126.54.

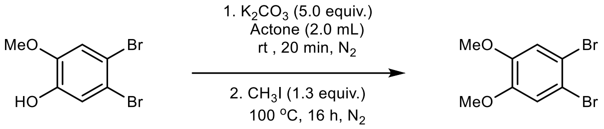

4a, 0.2 mmo **2a**, 91%

To a solution of 1,2-dibromo-4-hydroxy-5-methoxybenzene **4a** (56.38 mg, 0.2 mmol, 1.0 equiv.) in acetone (2 mL) was added K_2CO_3 (138.21 mg, 1.0 mmol, 5.0 equiv.), and stirred for 20 min under N_2. Iodomethane (36.90 mg, 0.26 mmol, 1.3 equiv.) was added into the flask and stirred at 70 °C for 16 h under N_2. The mixture was then filtered, the filtrate was vacuum-concentrated and purified by column chromatography on silica gel (300-400 mesh) with *n*-hexane/ethyl acetate (10:1) to yield 1,2-dibromo-4,5-dimethoxybenzene **2a** (53.86 mg, 91% yield) as a white solid[4]. ^{1}H NMR (500 MHz, $CDCl_3$): δ 7.06 (s, 2H), 3.86 (s, 6H); ^{13}C NMR (126 MHz, $CDCl_3$): δ 148.90, 115.93, 114.79, 56.30.

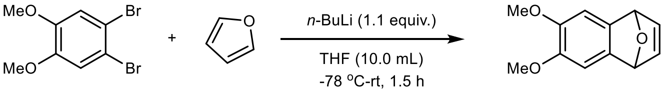

2a, 1.0 mmol **25**, 56%

Under an atmosphere of nitrogen, a two-necked bottle with a magnetic stir bar was charged with 1,2-dibromo-4,5-dimethoxybenzene **2a** (295.95 mg, 1.0 mmol, 1.0 equiv.), anhydrous THF (10.0 mL) and freshly distilled furan (2 mL). Then *n*-BuLi (2.5 M in hexane, 0.44 mL, 1.10 mmol, 1.1 equiv.) was added dropwise at -78 °C. The solution was stirred at -78 °C for 1.5 h. Subsequently, 10.0 mL distillated water was added to the reaction mixture and left it warm up to room temperature. Diethyl ether was added to the reaction mixture and the organic phase was separated. The aqueous solution was extracted with diethyl ether and the combined organic solution was dried over $MgSO_4$. The ether was then removed in vacuo and the resulting

mixture was purified by column chromatography on silica gel (300-400 mesh) with *n*-hexane/ethyl acetate (5:1) to afford the product 6,7-dimethoxy-1,4-dihydro-1,4-epoxynaphthalene **25** (114.36 mg, 56% yield) as a light brown solid[5]. ^{1}H NMR (500 MHz, $CDCl_3$): δ 7.04 (s, 2H), 6.97 (s, 2H), 5.68 (s, 2H), 3.85 (s, 6H); ^{13}C NMR (126 MHz, $CDCl_3$): δ 145.89, 143.39, 141.77, 106.81, 82.63, 56.51.

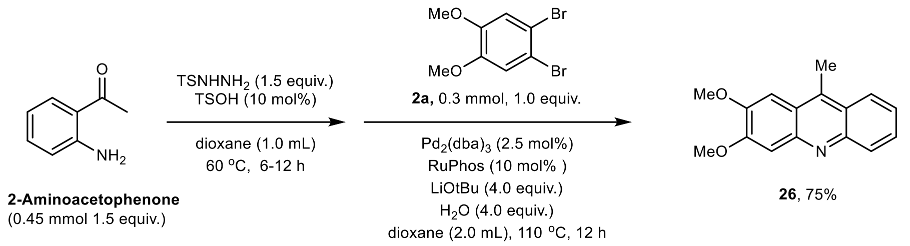

An oven-dried microwave tube was charged with a 2-aminoacetophenone (60.82 mg, 0.45 mmol, 1.5 equiv.), 4-methylbenzenesulfonohydrazide ($TsNHNH_2$) (83.80 mg, 0.45 mmol, 1.5 equiv.), a catalytic amount of 4-methylbenzenesulfonic acid (TsOH) (5.16 mg, 0.03 mmol, 10 mol%) and 1,4-dioxane (1 mL). The tube was sealed using a cap with a septum, and heated to 60 °C. The mixture was stirred at this temperature for 6–12 h, and the reaction was monitored by TLC.

After completion of the reaction, the mixture was allowed to cool to room temperature. $Pd_2(dba)_3$ (6.86 mg, 0.0075 mmol, 2.5 mol%), RuPhos (13.99 mg, 0.03 mmol, 10 mol%), LiO*t*Bu (96.06 mg, 1.2 mmol, 4.0 equiv.), 1,2-dibromo-4,5-dimethoxybenzene **2a** (88.78 mg, 0.3 mmol, 1.0 equiv.) and water (21.61 mg, 1.2 mmol, 4.0 equiv.) were added into the mixture. The tube was evacuated and back-filled with N_2 three times, and then it was heated to 110 °C. The mixture was stirred at this temperature for 12 h, and then it was allowed to cool to room temperature. The mixture was filtered through a short silica plug. The filtrate was concentrated in vacuo and purified by column chromatography on silica gel (300-400 mesh) with *n*-hexane/ethyl acetate (3:1) to afford 2,3-dimethoxy-9-methylacridine **26** (56.99 mg, 75% yield) as a yellow solid[6]. ^{1}H NMR (500 MHz, $CDCl_3$): δ 8.23-8.15 (m, 2H), 7.74-7.71 (m, 1H), 7.56-7.48 (m, 2H), 7.31 (s, 1H), 4.10 (d, J = 0.9 Hz, 6H), 3.05 (s, 3H); ^{13}C

NMR (126 MHz, $CDCl_3$): δ 153.66, 149.58, 146.88, 146.53, 138.76, 129.17, 128.58, 124.48, 124.39, 123.77, 121.61, 106.28, 100.42, 55.94, 55.63, 13.58.

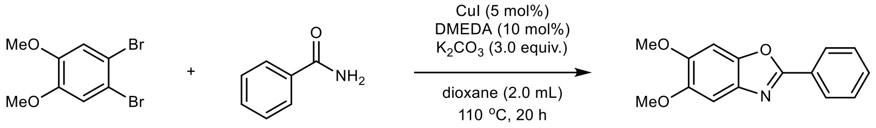

2a, 0.2 mmol **benzamide** (1.5 equiv.) **27**, 60%

A 10 mL Schlenk tube was charged with a benzamide (36.34 mg, 0.3 mmol, 1.5 equiv.) and dioxane (2 mL). Then, CuI (1.90 mg, 0.01 mmol, 5 mol%), DMEDA (1.76 mg, 0.02 mmol, 10 mol%), K_2CO_3 (82.92 mg, 0.6 mmol, 3.0 equiv.) and 1,2-dibromo-4,5-dimethoxybenzene **2a** (59.19 mg, 0.2 mmol, 1.0 equiv.) were added in under air, the reaction mixture was stirred for 20 h at 110 °C. The reaction mixture cooled down to room temperature. The mixture was then filtered, the filtrate was vacuum-concentrated and purified by column chromatography on silica gel (300-400 mesh) with *n*-hexane/ethyl acetate (5:1) to afford 5,6-dimethoxy-2-phenylbenzo[d]oxazole **27** (30.63 mg, 60% yield) as a white solid[7]. ^{1}H NMR (500 MHz, $CDCl_3$): δ 8.20-8.16 (m, 2H), 7.50 (dd, J = 5.1, 2.0 Hz, 3H), 7.26 (s, 1H), 7.14 (s, 1H), 3.96 (s, 3H), 3.95 (s, 3H); ^{13}C NMR (126 MHz, $CDCl_3$): δ 162.27, 148.41, 147.78, 145.14, 134.90, 130.88, 128.88, 127.48, 126.99, 101.74, 94.32, 56.51, 56.44.

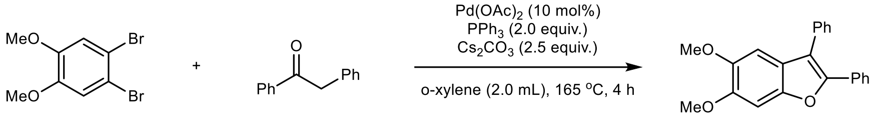

2a, (0.2 mmol) **1,2-diphenylethan-1-one** (1.0 equiv.) **28**, 58%

Dry, degassed *o*-xylene (2 mL) was added to an oven-dried reaction flask charged with $Pd(OAc)_2$ (4.53 mg, 0.02 mmol, 10 mol%), Cs_2CO_3 (162.91 mg, 0.5 mmol, 2.5 equiv.), PPh_3 (104.92 mg, 0.4 mmol, 2.0 equiv.), 1,2-diphenylethan-1-one (39.25 mg, 0.2 mmol, 1.0 equiv.) and 1,2-dibromo-4,5-dimethoxybenzene **2a** (59.19 mg, 0.2 mmol, 1.0 equiv.) under N_2 at room temperature. The resultant stirred suspension was heated to 165 °C for 4 h. The reaction mixture cooled down to room temperature. The mixture was then filtered, the filtrate was vacuum-concentrated and purified by

column chromatography on silica gel (300-400 mesh) with *n*-hexane/ethyl acetate (5:1) to afford 5,6-dimethoxy-2,3-diphenylbenzofuran **28** (38.32 mg, 58% yield) as a white powder[8]. ^{1}H NMR (500 MHz, $CDCl_3$): δ 7.61-7.57 (m, 2H), 7.53-7.46 (m, 4H), 7.44-7.39 (m, 1H), 7.31-7.26 (m, 3H), 7.13 (s, 1H), 6.89 (s, 1H), 3.97 (s, 3H), 3.87 (s, 3H); ^{13}C NMR (126 MHz, $CDCl_3$): δ 149.70, 148.71, 148.45, 146.83, 133.16, 130.96, 129.72, 129.07, 128.40, 127.79, 127.61, 126.44, 122.18, 117.76, 101.17, 95.16, 56.47, 56.37.

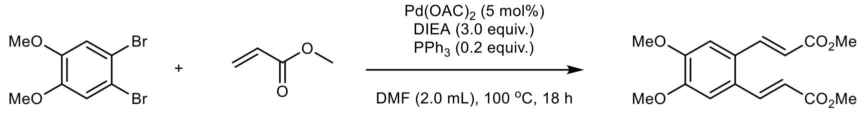

2a, 0.2 mmol **methyl acrylate** (5.0 equiv.) **29**, 78%

1,2-dibromo-4,5-dimethoxybenzene **2a** (59.19 mg, 0.2 mmol, 1.0 equiv.) and methyl acrylate (86.09 mg, 1.0 mmol, 5.0 equiv.) were added to a suspension of $Pd(OAc)_2$ (2.26 mg, 0.25 mmol, 5 mol%), DIEA (77.54 mg, 0.6 mmol, 3.0 equiv.), PPh_3 (10.49 mg, 0.04 mmol, 0.2 equiv.) in DMF (2 mL) under nitrogen atmosphere. The mixture was heated to 100 °C by oil bath and stirred for 18 h, cooled down to room temperature, diluted with Et_2O, and washed with H_2O. The aqueous layer was extracted with Et_2O, and the combined organic layers were washed with brine, dried with anhydrous Na_2SO_4, and concentrated in vacuo. The residue was purified by column chromatography on silica gel (300-400 mesh) with *n*-hexane/ethyl acetate (5:1) to afford dimethyl 3,3'-(4,5-dimethoxy-1,2-phenylene)(2E,2'E)-diacrylate **29** (47.78 mg, 78% yield) as a white solid[9]. ^{1}H NMR (500 MHz, $CDCl_3$): δ 8.05 (d, *J* = 15.8 Hz, 2H), 7.03 (s, 2H), 6.29 (d, *J* = 15.6 Hz, 2H), 3.94 (s, 6H), 3.83 (s, 6H); ^{13}C NMR (126 MHz, $CDCl_3$): δ 167.06, 150.80, 140.73, 127.67, 119.30, 109.01, 56.02, 51.82.

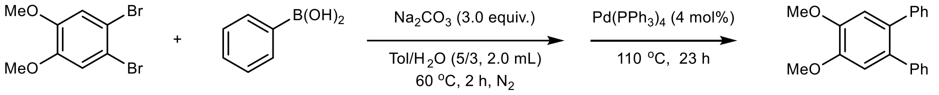

2a, 0.2 mmol **phenylboronic acid** (3.0 equiv.) **30**, 90%

A 10 mL Schlenk tube was charged with a 1,2-dibromo-4,5-dimethoxybenzene **2a** (59.19 mg, 0.2 mmol, 1.0 equiv.), phenylboronic acid (73.16 mg, 0.6 mmol, 3.0 equiv.), sodium carbonate (63.59 mg, 0.6 mmol, 3.0 equiv.) and toluene/water = 5:3 (2 mL) under nitrogen. The reaction mixture was vigorously stirred for 2 h at 60 °C. The catalyst $Pd(PPh_3)_4$ (9.24 mg, 0.008 mmol, 4 mol%) was then added and the reaction mixture was stirred for 23 h at 110 °C. Toluene was evaporated and the crude residue was extracted three times with CH_2Cl_2. The organic layers were combined and evaporated. The residue was purified by column chromatography on silica gel (300-400 mesh) with *n*-hexane/ethyl acetate (3:1) to afford 4',5'-dimethoxy-1,1':2',1''-terphenyl **30** (52.26 mg, 90% yield) as a white solid[10]. ^1H NMR (500 MHz, $CDCl_3$): δ 7.23-7.16 (m, 6H), 7.15-7.11 (m, 4H), 6.94 (s, 2H), 3.94 (s, 6H); ^{13}C NMR (126 MHz, $CDCl_3$): δ 148.21, 141.47, 133.05, 129.99, 127.91, 126.29, 113.68, 56.09.

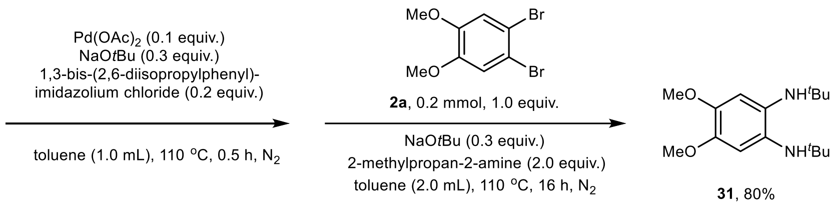

A 10 mL Schlenk tube was charged with 1,3-bis-(2,6-diisopropylphenyl)-imidazolium chloride (17.00 mg, 0.04 mmol, 0.2 equiv.), $Pd(OAc)_2$ (4.53 mg, 0.02 mmol, 0.1 equiv.), NaO^{t}Bu (5.76 mg, 0.06 mmol, 0.3 equiv.) and toluene (1 mL). The mixture was heated to 110 °C by oil bath and stirred for 0.5 h under nitrogen，after which it was cooled to room temperature and transferred to a Schlenk flask containing 1,2-dibromo-4,5-dimethoxybenzene **2a** (59.19 mg, 0.2 mmol, 1.0 equiv.) and NaO^{t}Bu (57.66 mg, 0.6 mmol, 3.0 equiv.) in toluene (2.0 mL). 2-methylpropan-2-amine (29.25 mg, 0.4 mmol, 2.0 equiv.) was added, the reaction heated to 110 °C and stirred for 16 h. The reaction mixture cooled down to room temperature. The mixture was then filtered, the filtrate was vacuum-concentrated and purified by column chromatography on silica gel (300-400 mesh) with *n*-hexane/ethyl acetate (5:1) to afford N^1,N^2-di-*tert*-butyl-4,5-dimethoxybenzene-1,2-diamine **31** (44.86 mg, 80% yield) as a yellow oil[11].

^{1}H NMR (500 MHz, $CDCl_3$): δ 6.59 (s, 2H), 3.82 (s, 6H), 1.24 (s, 18H); ^{13}C NMR (126 MHz, $CDCl_3$): δ 143.48, 132.69, 108.17, 56.42, 52.53, 30.20.

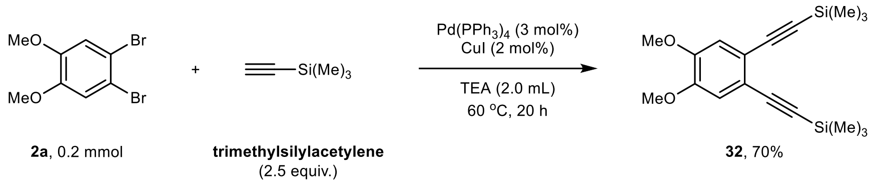

Under a nitrogen atmosphere, trimethylsilylacetylene (49.11 mg, 0.5 mmol, 2.5 equiv.) was added to a solution of 1,2-dibromo-4,5-dimethoxybenzene **2a** (59.19 mg, 0.2 mmol, 1.0 equiv.) in deoxygenated distilled triethylamine (TEA, 2 mL) with $Pd(PPh_3)_4$ (6.93 mg, 0.006 mmol, 3 mol%) and copper iodide (0.76 mg, 0.004 mmol, 2 mol%) in a 10 mL Schlenk flask at 0 ℃. After stirring for 1 hour at room temperature, the reaction system was heated to 60 ℃, and was further stirred for 20 hours at this temperature. The reaction system was cooled to room temperature and then a saturated aqueous solution of ammonium chloride was added. The aqueous solution was extracted three times with dichloromethane. The combined dichloromethane layer was washed with brine and dried over sodium sulfate. After evaporation of the dichloromethane, the crude residue was purified by column chromatography on silica gel (300-400 mesh) with *n*-hexane/ethyl acetate (3:1) to afford pure (4,5-dimethoxy-1,2-phenylene)bis(ethyne-2,1-diyl)bis(trimethylsilane) **32** (46.28 mg, 70% yield) as a white solid[12]. ^{1}H NMR (500 MHz, $CDCl_3$): δ 6.92 (s, 2H), 3.88 (s, 6H), 0.27 (s, 18H); ^{13}C NMR (126 MHz, $CDCl_3$): δ 149.04, 119.00, 114.40, 103.45, 96.88, 56.04, 0.19.

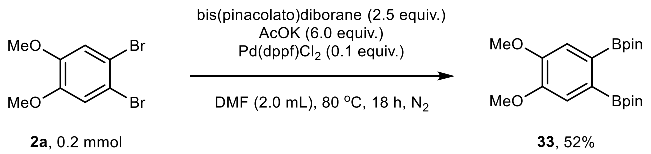

To bis(pinacolato)diborane (126.97 mg, 0.5 mmol, 2.5 equiv.), anhydrous potassium acetate (117.77 mg, 1.2 mmol, 6.0 equiv.) and a 1,2-dibromo-4,5-dimethoxybenzene **2a** (59.19 mg, 0.2 mmol, 1.0 equiv.), 2 mL of anhydrous DMF was added under

nitrogen. [1,1'-bis(diphenylphosphino)ferrocene]palladium(II) dichloride (14.63 mg, 0.02 mmol, 0.1 equiv.) was added and the mixture was stirred in nitrogen atmosphere at 80 °C for 18 h. The reaction mixture cooled down to room temperature. The mixture was then filtered, the filtrate was purified by column chromatography on silica gel (300-400 mesh) with *n*-hexane/ethyl acetate (5:1) to afford 2,2'-(4,5-dimethoxy-1,2-phenylene)bis(4,4,5,5-tetramethyl-1,3,2-dioxaborolane) **33** (40.57 mg, 52% yield) as a pale yellow oil[13]. ^{1}H NMR (500 MHz, $CDCl_3$): δ 7.15 (s, 2H), 3.91 (s, 6H), 1.36 (s, 24H); ^{13}C NMR (126 MHz, $CDCl_3$): δ 149.69, 116.17, 83.78, 55.75, 24.91.

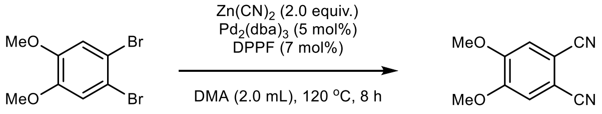

2a, 0.2 mmol **34**, 75%

A 10 mL Schlenk tube was charged with 1,2-dibromo-4,5-dimethoxybenzene **2a** (59.19 mg, 0.2 mmol, 1.0 equiv.) in *N*,*N*-dimethylacetamide (2 mL). The reaction mixture was heated to 120°C on oil bath and $Pd_2(dba)_3$ (9.16 mg, 0.01 mmol, 5 mol%) and dppf (7.76 mg, 0.014 mmol, 7 mol%) were added. Afterwards, $Zn(CN)_2$ (46.97 mg, 0.4 mmol, 2.0 equiv.) was added in 4-5 portions during the 2 h till TLC indicated completion of the reaction. The reaction mixture was cooled, diluted with EtOAc and filtered, the filtrate was vacuum-concentrated and purified by column chromatography on silica gel (300-400 mesh) with *n*-hexane/ethyl acetate (5:1) to afford 4,5-dimethoxyphthalonitrile **34** (28.22 mg, 75% yield) as a white powder[14]. ^{1}H NMR (500 MHz, $CDCl_3$): δ 7.16 (s, 2H), 3.97 (s, 6H); ^{13}C NMR (126 MHz, $CDCl_3$): δ 152.61, 115.76, 114.79, 108.96, 56.68.

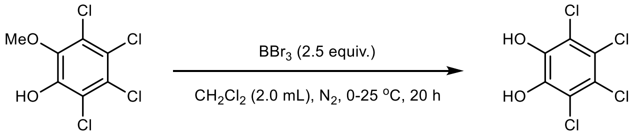

12a, 0.2 mmol **35**, 40%

Boron tribromide (1 M in CH_2Cl_2, 0.5 mL, 0.5 mmol, 2.5 equiv.) was added slowly to a solution of 2,3,4,5-tetrachloro-6-methoxyphenol **12a** (52.3 mg, 0.2 mmol, 1.0 equiv.) in CH_2Cl_2 (2 mL) and the resulting reaction mixture was stirred at 0 °C in N_2 atmosphere for 1 hour and raised to room temperature for 20 h. The reaction was quenched by the addition of 0.1 M HCl and the resulting biphasic mixture was saturated with sodium chloride. The layers were separated and the aqueous phase was extracted with CH_2Cl_2. The combined organic layers were dried (Na_2SO_4) and concentrated in vacuo and the residue was purified by column chromatography on silica gel (300-400 mesh) with *n*-hexane/ethyl acetate (3:1) to afford 3,4,5,6-tetrachloro-2-benzenediol **35** (19.83 mg, 40% yield) as light brown solid[15]. ^1H NMR (500 MHz, $CDCl_3$): δ 5.96 (s, 2H); ^{13}C NMR (126 MHz, $CDCl_3$): δ 140.00, 123.78, 118.87.

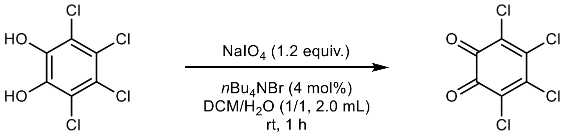

35, 0.2 mmol **36**, 80%

The compound 3,4,5,6-tetrachloro-2-benzenediol **35** (49.57 mg, 0.2 mmol, 1.0 equiv.) was dissolved in 1 mL of CH_2Cl_2. After adding sodium periodate (51.81 mg, 0.24 mmol, 1.20 equiv.) and (n-butyl)$_4$NBr (2.57 mg, 0.008 mmol, 4 mol%), dissolved in 1.0 mL of DI water, the reaction mixture was stirred for 1 h at room temperature. After extraction with CH_2Cl_2, the combined organic layers were dried (Na_2SO_4) and concentrated in vacuo and the residue was purified by column chromatography on silica gel (300-400 mesh) with *n*-hexane/ethyl acetate (3:1) to afford 3,4,5,6-tetrachlorocyclohexa-3,5-diene-1,2-dione **36** (39.34 mg, 80% yield) as red powder[16]. ^{13}C NMR (126 MHz, $CDCl_3$): δ 168.99, 143.92, 132.10.

Supplementary Discussion

Investigation for activation of [X^+] reagent

In order to verify the possibility of this depolymerization and halogenation reaction, the activation of NBS by TFE was preliminarily studied. The effect of TFE on the chemical shifts of methylene hydrogen and carbonyl carbon was analyzed to determine the effect of TFE on the activation of NBS. As shown in Supplementary Figure 8, in the presence of TFE, the ^{1}H NMR signal of methylene in NBS shifts to the low field, indicating that the electron density of methylene group decreases. At the same time, with the increase of the amount of TFE, the ^{13}C NMR signal of carbonyl carbon shifts to the lower field, indicating that the electron density on the carbonyl group decreased, which was transferred to the bromine atom in NBS through hydrogen bonding, and the electron density on the bromine atom also decreased, further increasing its reactivity.

Reactivity of lignin linkages for research of bond cleavage

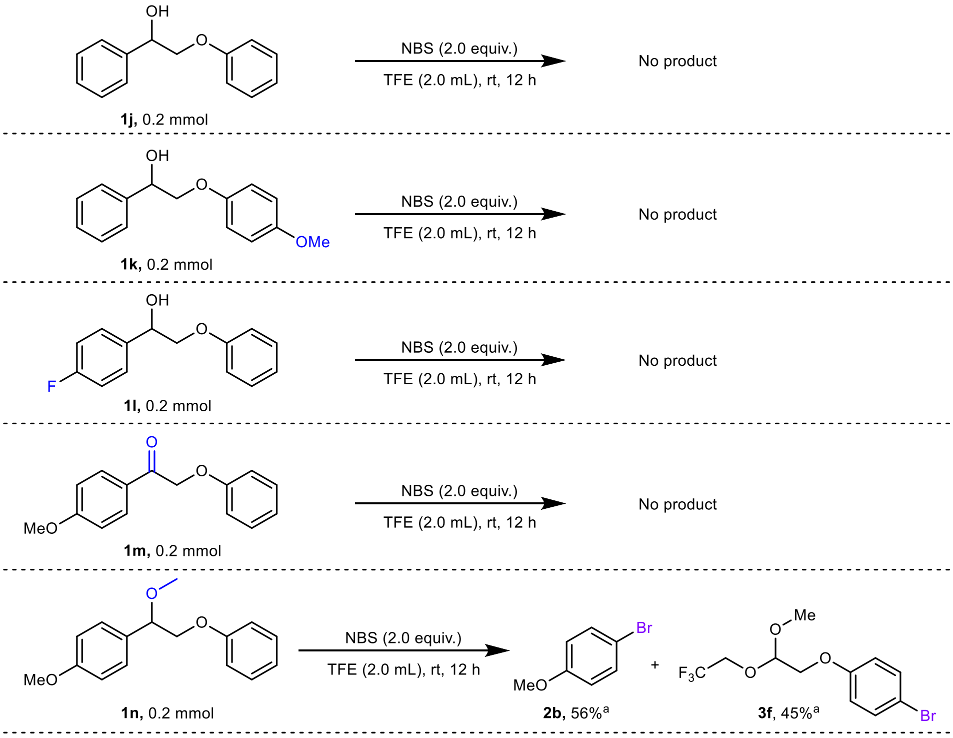

[a]Isolated Yields.

Polysubstituted product formation

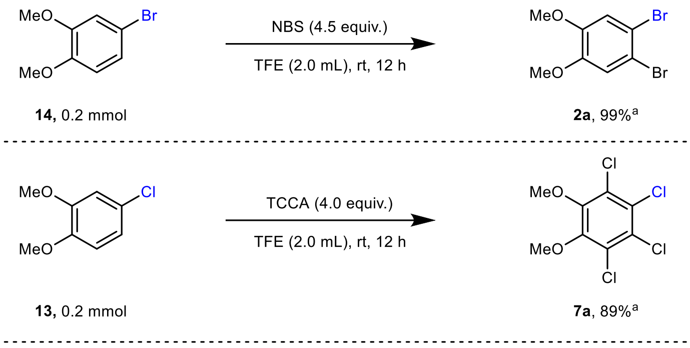

[a]Yields determined by ^{1}H NMR spectroscopy of the crude mixture with CH_2Br_2 as an internal standard.

Under standard conditions, the reaction of monosubstituted 3,4-dimethoxy-1-bromobenzene **14** and 3,4-dimethoxy-1-chlorobenzene **13** yielded a target product with high yields, confirming the formation of polysubstituted aryl halides.

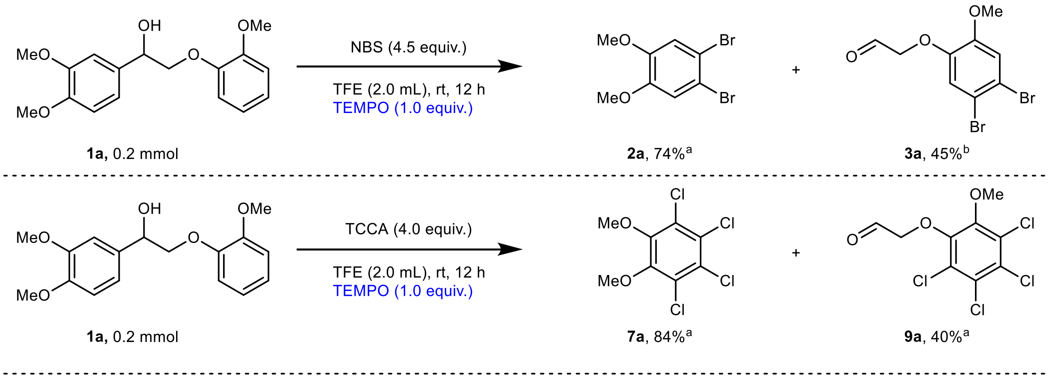

[a]Yields determined by ^{1}H NMR spectroscopy of the crude mixture with CH_2Br_2 as an internal standard.[b]Yields determined by GC-MS with n-*o*ctadecane as an internal standard.

Bromine radical trapping experiment: A 10 mL Schlenk tube was charged with Lignin model compound **1a** (60.86 mg, 0.2 mmol, 1.0 equiv.), NBS (160.18 mg, 0.9 mmol, 4.5 equiv.), 2,2,6,6-tetramethylpiperidinooxy (TEMPO) (31.25 mg, 0.2 mmol, 1.0 equiv.) and TFE (2 mL) at air atmosphere. The reaction was stirred at room temperature for 12 h. After the reaction was completed, CH_2Br_2 was used as the internal standard, the yield of **2a** in the mixture was detected by ^{1}H NMR spectroscopy, and the yield of **3a** in the mixture was detected by GC-MS with *n*-octadecane as the internal standard. ^{1}H NMR yield: **2a** (74%). GC-MS Yield: **3a** (45%).

Chlorine radical trapping experiment: A 10 mL Schlenk tube was charged with Lignin model compound **1a** (60.86 mg, 0.2 mmol, 1.0 equiv.), TCCA (185.92 mg, 0.8

mmol, 4.0 equiv.), TEMPO (31.25 mg, 0.2 mmol, 1.0 equiv.) and TFE (2 mL) at air atmosphere. The reaction was stirred at room temperature for 12 h. After the reaction was completed, CH_2Br_2 was used as the internal standard, the yield of **7a** and **9a** in the mixture was detected by ^{1}H NMR spectroscopy. ^{1}H NMR yield: **7a** (84%), **9a** (40%).

New compounds characterization

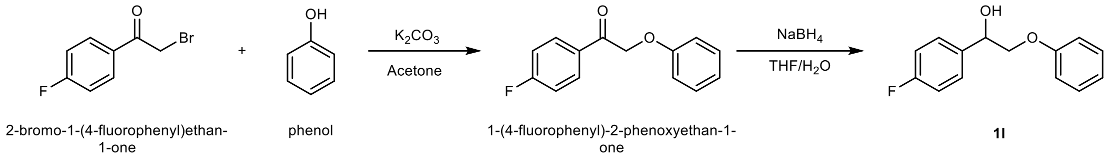

1l were prepared in a two-step process from the reaction of corresponding 2-bromo-1-(4-fluorophenyl)ethan-1-one and phenol according to the literature procedure[17].

2-Bromo-1-(4-fluorophenyl)ethan-1-one (1.30 g, 6.0 mmol, 1.0 equiv.) was added to a stirred solution containing K_2CO_3 (1.24 g, 9.0 mmol, 1.5 equiv.), phenol (0.70 g, 7.5 mmol, 1.25 equiv.) and acetone (30 mL), then stirred at rt for 5 h. After filtration, the filtrate was concentrated under the reduced pressure and the crude mixture was purified by column chromatography on silica gel (300-400 mesh) with *n*-hexane/ethyl acetate (3:1) to afford 1-(4-fluorophenyl)-2-phenoxyethan-1-one (1.24 g, 90% yield).

The resulting compound (0.92 g, 4.0 mmol, 1.0 equiv.) was dissolved in the mixed solvents of THF and H_2O (3:1, 24 mL), then portion-wisely added with sodium borohydride (0.30 g, 8.0 mmol, 2.0 equiv.). After stirring at rt for 6 h, the mixture was quenched with saturated aqueous NH_4Cl (50 mL) and diluted with 30 mL water. The aqueous portion was extracted with ethyl acetate (3 × 30 mL). The combined organic layers were dried over $MgSO_4$, filtered and concentrated under vacuum. The residue was purified by column chromatography on silica gel (300-400 mesh) with *n*-hexane/ethyl acetate (1:1) to afford 1-(4-fluorophenyl)-2-phenoxyethan-1-ol **1l** as a yellow oil (0.60 g, 65% yield). ^{1}H NMR (500 MHz, $CDCl_3$): δ 7.38 (dd, *J* = 8.5, 5.5 Hz, 2H), 7.27 (dd, *J* = 8.8, 7.3 Hz, 2H), 7.08-7.01 (m, 2H), 6.96 (tt, *J* = 7.4, 1.1 Hz,

1H), 6.91-6.86 (m, 2H), 5.05 (dd, J = 8.7, 3.2 Hz, 1H), 4.03 (dd, J = 9.6, 3.3 Hz, 1H), 3.95 (dd, J = 9.6, 8.6 Hz, 1H), 3.03 (s, 1H); ^{13}C NMR (126 MHz, $CDCl_3$): δ 163.58, 161.62, 158.34, 135.58, 135.55, 129.64, 128.09, 128.03, 121.46, 115.56, 115.39, 114.70, 73.23, 71.98. IR (ATR) v (cm^{-1}) = 3214, 3116, 3015, 2915, 1600, 1587, 1509, 1496, 1454, 1289, 1217, 1172, 1157, 1076, 1036, 913, 869, 833, 753, 688, 576. HRMS (m/z): $[M+H]^+$ calcd. for $C_{14}H_{14}FO_2$, 233.0972; found 233.0973.

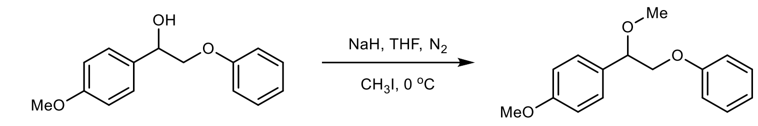

To a solution containing sodium hydride (0.14 g, 3.5 mmol, 60% in mineral oil, 1.75 equiv.) and anhydrous THF (10 mL), 1-(4-methoxyphenyl)-2-phenoxyethan-1-ol (0.48 g, 2.0 mmol, 1.0 equiv.) in THF (10 mL) was added in two portions at 0 °C under nitrogen atmosphere. After stirring at 0 °C for 1 h, the resulting suspension was added with iodomethane (0.49 g, 3.5 mmol, 1.75 equiv.) dropwise. The reaction mixture was stirred overnight and monitored by TLC, then quenched with saturated ammonium chloride solution. The aqueous layer was extracted with ethyl acetate (3 × 30 mL). The combined organic extracts were washed with brine, dried over Na_2SO_4 and concentrated in vacuo. The crude product was purified by column chromatography on silica gel (300-400 mesh), first with *n*-hexane to remove the mineral oil followed by gradient eluent of 5-12% EtOAc in *n*-hexane to afford 1-methoxy-4-(1-methoxy-2-phenoxyethyl)benzene **1n** as a yellow oil. (0.35 g, 69% yield). ^{1}H NMR (500 MHz, $CDCl_3$): δ 7.33-7.28 (m, 2H), 7.24 (dd, J = 8.7, 7.3 Hz, 2H), 6.95-6.86 (m, 5H), 4.53 (dd, J = 7.9, 3.7 Hz, 1H), 4.15 (dd, J = 10.2, 7.9 Hz, 1H), 3.96 (dd, J = 10.2, 3.8 Hz, 1H), 3.79 (s, 3H), 3.32 (s, 3H); ^{13}C NMR (126 MHz, $CDCl_3$): δ 159.66, 158.81, 130.59, 129.43, 128.30, 120.94, 114.83, 114.06, 81.78, 72.34, 56.97, 55.31. IR (ATR) v (cm^{-1}) = 3012, 2926, 2831, 1596, 1584, 1510, 1493, 1449, 1302, 1236, 1170, 1105, 1075, 1030, 827, 754, 689, 550. HRMS (m/z): $[M+Na]^+$ calcd. for $C_{16}H_{18}NaO_3$, 281.1148, found 281.1141.

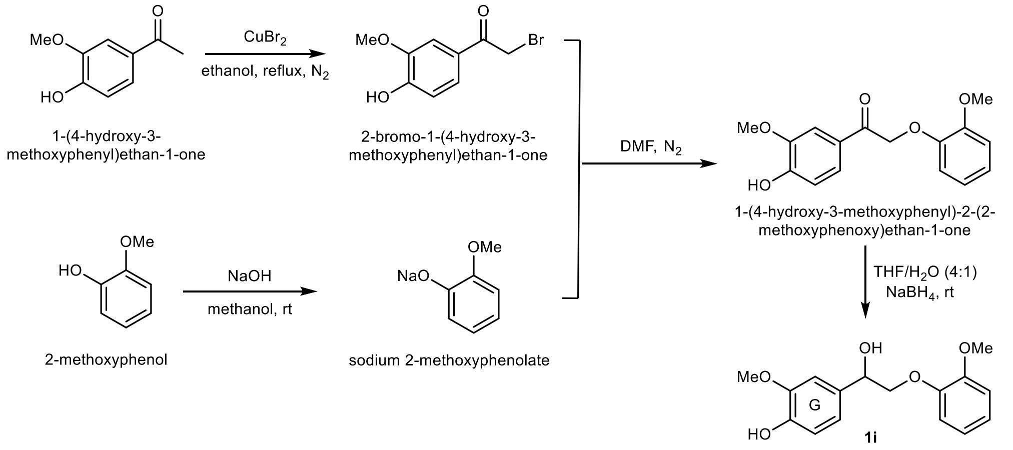

To a solution of 1-(4-hydroxy-3-methoxyphenyl)ethan-1-one (1.66 g, 0.01 mol, 1.0 equiv.) in ethanol was added $CuBr_2$ (4.44 g, 0.02 mol, 2.0 equiv.), the mixture was stirred at 80 °C for 5 h. After filtration, the filtrate was concentrated under the reduced pressure and the crude mixture was purified via flash column chromatography to afford 2-bromo-1-(4-hydroxy-3-methoxyphenyl)ethan-1-one (2.21 g, 90% yield).

2-Methoxyphenolate was synthesized by mixing NaOH (1.59 g, 0.04 mol, 4.0 equiv.) with guaiacol (2.48 g, 0.02 mol, 2.0 equiv.) in methanol, and the mixture was stirred overnight. The solvent was removed by evaporation and white solid was obtained. Then this white solid was mixed with 2-bromo-1-(4-hydroxy-3-methoxyphenyl)ethan-1-one (2.21 g) in DMF at 0 °C, the mixture was stirred at room temperature for overnight. Diluted HCl aqueous was used to adjust the pH value of the solution to 3, then the solution was extracted with $CHCl_3$ for three times. The combined organic phase was dried by anhydrous Na_2SO_4 and filtered. The solvent was removed by evaporation and residuum was purified by column chromatography on silica gel (300-400 mesh) with *n*-hexane/ethyl acetate (10:1) to give 1-(4-hydroxy-3-methoxyphenyl)-2-(2-methoxyphenoxy)ethan-1-one (2.25 g, 78% yield) as a white solid product.

The resulting compound (2.00 g, 7.0 mmol, 1.0 equiv.) was dissolved in the mixed solvents of THF and H_2O (4:1, 25 mL), then portion-wisely added with sodium borohydride (0.53 g, 14.0 mmol, 2.0 equiv.). After stirring at rt for 6 h, the mixture was quenched with saturated aqueous NH_4Cl (20 mL) and diluted with 15 mL water. The aqueous portion was extracted with ethyl acetate (3 × 30 mL). The combined

organic layers were dried over $MgSO_4$, filtered and concentrated under vacuum. The residue was purified by column chromatography on silica gel (300-400 mesh) with *n*-hexane/ethyl acetate (3:1) to afford 4-(1-hydroxy-2-(2-methoxyphenoxy)ethyl)-2-methoxyphenol **1i** as a white powder (1.72 g, 85% yield). ^{1}H NMR (500 MHz, $CDCl_3$): δ 7.05–6.97 (m, 2H), 6.96–6.86 (m, 5H), 5.67 (s, 1H), 5.03 (dd, *J* = 9.4, 2.9 Hz, 1H), 4.15 (dd, *J* = 10.0, 2.9 Hz, 1H), 3.95 (t, *J* = 9.7 Hz, 1H), 3.90 (s, 3H), 3.88 (s, 3H), 3.49 (s, 1H). ^{13}C NMR (126 MHz, $CDCl_3$): δ 150.14, 147.98, 146.66, 145.41, 131.51, 122.57, 121.10, 119.37, 116.03, 114.25, 111.97, 108.76, 76.41, 72.17, 55.95, 55.85. IR (ATR) *v* (cm^{-1}) = 3486, 3470, 3203, 3015, 2973, 2963, 2941, 2840, 1735, 1602, 1578, 1547, 1494, 1439, 1375, 1336, 1235, 1204, 1091, 1047, 926, 880, 839, 806, 681, 585, 566. HRMS (m/z): $[M+Na]^+$ calcd. for $C_{16}H_{18}NaO_5$, 313.1046, found 313.1042.

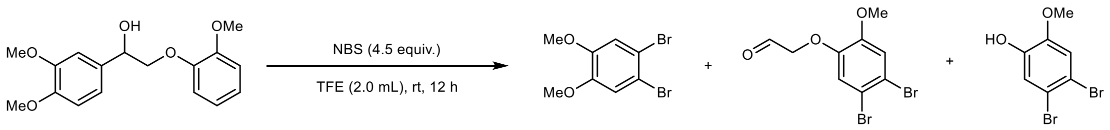

1a, 0.2 mmol **2a**, 99% **3a**, 61% **4a**, 15%

The reaction was conducted following the general procedure 1 in a 0.2 mmol scale for 12 h. The residue was purified by column chromatography on silica gel (300-400 mesh) with petroleum ether/ethyl acetate (10:1) to afford the product **2a** as a white solid (58.60 mg, 99% yield) and on silica gel with petroleum ether/ethyl acetate (5:1) to afford **3a** as a white solid (39.52 mg, 61% yield) and **4a** as a white solid (8.46 mg, 15% yield). **2a:** ^{1}H NMR (500 MHz, $CDCl_3$): δ 7.06 (s, 2H), 3.86 (s, 6H); ^{13}C NMR (126 MHz, $CDCl_3$): δ 148.90, 115.93, 114.79, 56.30. **3a:** ^{1}H NMR (500 MHz, $CDCl_3$): δ 9.86 (t, *J* = 0.9 Hz, 1H), 7.13 (s, 1H), 7.04 (s, 1H), 4.60 (d, *J* = 0.9 Hz, 2H), 3.87 (s, 3H); ^{13}C NMR (126 MHz, $CDCl_3$): δ 198.28, 149.52, 146.96, 119.32, 117.19, 116.84, 114.74, 74.23, 56.33. IR (ATR) *v* (cm^{-1}) = 3014, 2929, 2848, 1735, 1589, 1495, 1438, 1356, 1250, 1207, 1179, 1061, 1027, 911, 852, 809, 735, 650, 584. HRMS (m/z): $[M+H]^+$ calcd. for $C_9H_9Br_2O_3$, 324.8892, found 324.8887. **4a**: ^{1}H NMR (500 MHz,

$CDCl_3$): δ 7.18 (s, 1H), 7.06 (s, 1H), 5.58 (s, 1H), 3.88 (s, 3H); ^{13}C NMR (126 MHz, $CDCl_3$): δ 146.45, 145.57, 119.15, 115.50, 115.36, 113.79, 56.36.

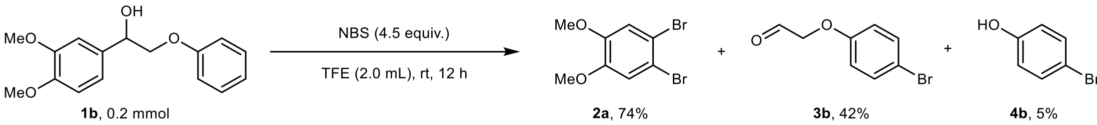

The reaction was conducted following the general procedure 1 in a 0.2 mmol scale for 12 h. The residue was purified by column chromatography on silica gel (300-400 mesh) with petroleum ether/ethyl acetate (10:1) to afford the product **2a** (43.80 mg, 74% yield) and on silica gel with petroleum ether/ethyl acetate (5:1) to afford **3b** as a white solid (18.06 mg, 42% yield) and **4b** as a brown solid (1.74 mg, 5% yield) [19]. **3b**: ^1H NMR (500 MHz, $CDCl_3$): δ 9.85 (t, J = 1.0 Hz, 1H), 7.42 (d, J = 9.0 Hz, 2H), 6.79 (d, J = 8.9 Hz, 2H), 4.56 (d, J = 1.1 Hz, 2H); ^{13}C NMR (126 MHz, $CDCl_3$): δ 198.54, 156.74, 132.63, 116.39, 114.32, 72.81. IR (ATR) ν (cm^{-1}) = 3020, 2919, 2887, 1775, 1712, 1598, 1587, 1501, 1486, 1466, 1376, 1285, 1242, 1043, 1006, 818, 721, 695, 601. HRMS: m/z (ESI-TOF) calculated for $C_8H_{11}BrNO_2$ $[M+NH_4]^+$: 231.9967, found 231.9970. **4b**: ^1H NMR (500 MHz, $CDCl_3$): δ 7.33 (d, J = 8.7 Hz, 2H), 6.72 (d, J = 8.7 Hz, 2H), 4.89 (s, 1H). ^{13}C NMR (126 MHz, $CDCl_3$): δ 154.53, 132.52, 117.22, 112.99.

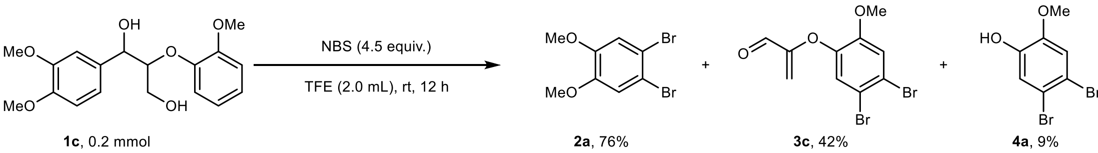

The reaction was conducted following the general procedure 1 in a 0.2 mmol scale for 12 h. The residue was purified by column chromatography on silica gel (300-400 mesh) with petroleum ether/ethyl acetate (10:1) to afford the product **2a** (44.98 mg, 76% yield) and on silica gel with petroleum ether/ethyl acetate (5:1) to afford **3c** as a yellow oil (28.22 mg, 42% yield) and **4a** (5.07 mg, 9% yield). **3c**: ^1H NMR (500 MHz, $CDCl_3$): δ 9.43 (s, 1H), 7.28 (s, 1H), 7.21 (s, 1H), 5.37 (d, J = 3.1 Hz, 1H), 5.21 (d, J = 3.1 Hz, 1H), 3.81 (s, 3H); ^{13}C NMR (126 MHz, $CDCl_3$): δ 186.24, 157.35, 150.79,

142.67, 126.11, 120.95, 117.79, 114.86, 108.97, 56.37. IR (ATR) *v* (cm^{-1}) = 3023, 2923, 2846, 2256, 1703, 1613, 1583, 1486, 1436, 1351, 1310, 1257, 1178, 1108, 1029, 970, 909, 855, 732, 667, 644. HRMS (m/z): $[M+H]^+$ calcd. for $C_{10}H_9Br_2O_3$, 336.8892, found 336.8887.

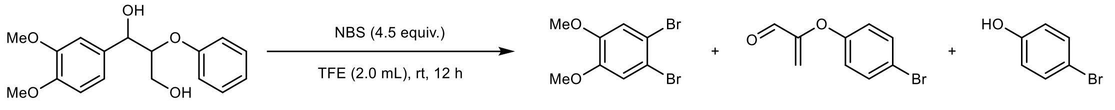

The reaction was conducted following the general procedure 1 in a 0.2 mmol scale for 12 h. The residue was purified by column chromatography on silica gel (300-400 mesh) with petroleum ether/ethyl acetate (10:1) to afford the product **2a** (58.60 mg, 99% yield) and on silica gel with petroleum ether/ethyl acetate (5:1) to afford **3d** as a yellow oil (11.35 mg, 25% yield) and **4b** (1.76 mg, 5% yield). **3d**: ^{1}H NMR (500 MHz, $CDCl_3$): δ 9.45 (s, 1H), 7.49 (d, *J* = 8.8 Hz, 2H), 6.94 (d, *J* = 8.8 Hz, 2H), 5.44 (d, *J* = 2.8 Hz, 1H), 5.36 (d, *J* = 2.9 Hz, 1H); ^{13}C NMR (126 MHz, $CDCl_3$): δ 186.83, 158.24, 153.73, 133.03, 121.78, 117.77, 110.47. IR (ATR) *v* (cm^{-1}) = 3101, 2924, 2885, 2143, 1711, 1612, 1582, 1484, 1469, 1375, 1312, 1222, 1071, 1046, 1011, 965, 911, 735, 651, 580. HRMS (m/z): $[M+H]^+$ calcd. for $C_9H_8BrO_2$, 226.9702, found 226.9710.

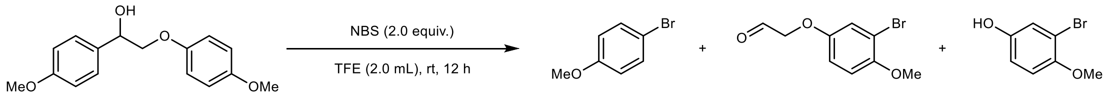

The reaction was conducted following the general procedure 2 in a 0.2 mmol scale for 12 h. The residue was purified by column chromatography on silica gel (300-400 mesh) with petroleum ether/ethyl acetate (10:1) to afford the product **2b** as a colorless oil (35.53 mg, 95% yield)[20] and on silica gel with petroleum ether/ethyl acetate (5:1) to afford **3e** as a yellow oil (22.54 mg, 46% yield) and **4c** as a brown powder (4.06 mg, 10% yield)[21]. **2b:** ^{1}H NMR (500 MHz, $CDCl_3$) δ 7.39 (d, *J* = 9.0 Hz, 2H), 6.79 (d, *J* = 9.0 Hz, 2H), 3.78 (s, 3H); ^{13}C NMR (126 MHz, $CDCl_3$) δ 158.75, 132.27, 115.78,

112.83, 55.42. **3e:** ^{1}H NMR (500 MHz, $CDCl_3$): δ 9.84 (t, *J* = 1.1 Hz, 1H), 7.16 (dd, *J* = 2.6, 0.7 Hz, 1H), 6.85-6.83 (m, 2H), 4.53 (d, *J* = 1.1 Hz, 2H), 3.86 (s, 3H); ^{13}C NMR (126 MHz, $CDCl_3$): δ 198.82, 151.92, 151.31, 120.23, 114.37, 112.74, 112.20, 73.51, 56.78. IR (ATR) *v* (cm^{-1}) = 3033, 2989, 2838, 2502, 2072, 1737, 1600, 1574, 1494, 1440, 1374, 1273, 1241, 1093, 1043, 848, 807, 752, 642, 572. HRMS (m/z): $[M+H]^+$ calcd. for $C_9H_{10}BrO_3$, 244.9807, found 244.9805. **4c:** ^{1}H NMR (500 MHz, $CDCl_3$): δ 7.09-7.07 (m, 1H),6.81-6.74 (m, 2H), 4.76 (br, 1H), 3.83(s, 3H); ^{13}C NMR (126 MHz, $CDCl_3$): δ 150.42, 149.76, 120.55, 115.00, 113.13, 111.96, 56.94.

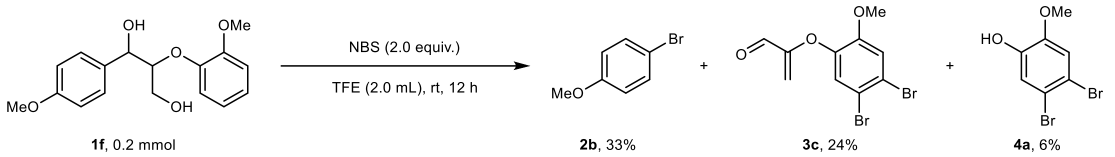

The reaction was conducted following the general procedure 3 in a 0.2 mmol scale for 12 h. The residue was purified by column chromatography on silica gel (300-400 mesh) with petroleum ether/ethyl acetate (10:1) to afford the product **2b** (12.34 mg, 33% yield) and on silica gel with petroleum ether/ethyl acetate (5:1) to afford **3c** (16.12 mg, 24% yield) and **4a** (3.38 mg, 6% yield).

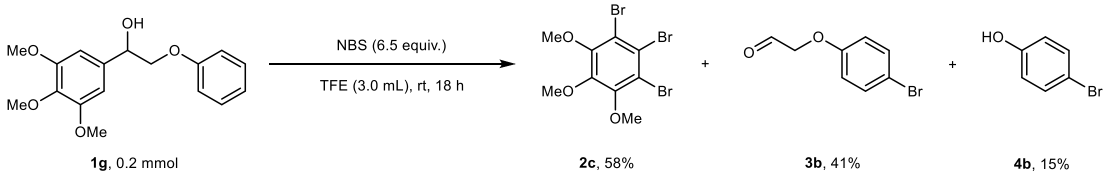

The reaction was conducted following the general procedure 3 in a 0.2 mmol scale for 18 h. The residue was purified by column chromatography on silica gel (300-400 mesh) with petroleum ether/ethyl acetate (10:1) to afford the product **2c** as a white solid (46.96 mg, 58% yield)[22] and on silica gel with petroleum ether/ethyl acetate (5:1) to afford **3b** as a white solid (17.63 mg, 41% yield) and **4b** (5.20 mg, 15% yield). **2c:** ^{1}H NMR (500 MHz, $CDCl_3$): δ 3.93 (s, 3H), 3.90 (s, 6H); ^{13}C NMR (126 MHz, $CDCl_3$): δ 151.70, 146.98, 122.11, 116.84, 61.38, 61.12.

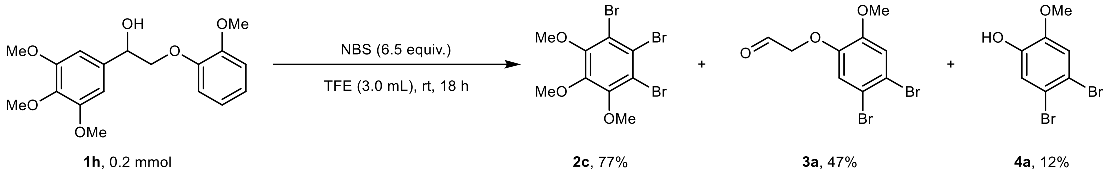

The reaction was conducted following the general procedure 3 in a 0.2 mmol scale for 18 h. The residue was purified by column chromatography on silica gel (300-400 mesh) with petroleum ether / ethyl acetate (10:1) to afford the product **2c** (62.35 mg, 77% yield) and on silica gel with petroleum ether/ethyl acetate (5:1) to afford **3a** (30.45 mg, 47% yield) and **4a** (6.77 mg, 12% yield).

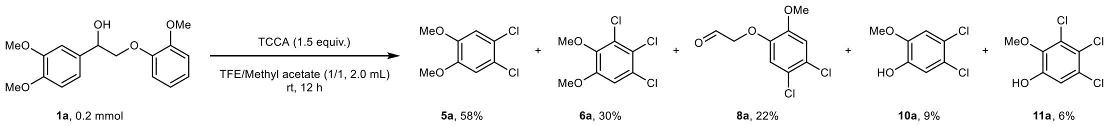

The reaction was conducted following the general procedure 4 in a 0.2 mmol scale for 12 h. The residue was purified by column chromatography on silica gel (300-400 mesh) with petroleum ether / ethyl acetate (10:1) to afford the product **5a** as a white solid (24.01 mg, 58% yield)[23], **6a** as a white solid (14.48 mg, 30%), on silica gel with petroleum ether / ethyl acetate (5:1) to afford **8a** as a white solid (10.34 mg, 22% yield). Yields of **10a**[24] and **11a** were determined by GC-MS and isolated by preparative thin-layer chromatography (PTLC) with petroleum ether/ethyl acetate (3:1). **5a:** ^{1}H NMR (500 MHz, $CDCl_3$): δ 6.91 (s, 2H), 3.87 (s, 6H); ^{13}C NMR (126 MHz, $CDCl_3$): δ 148.32, 123.47, 112.92, 56.33. **6a:** ^{1}H NMR (500 MHz, $CDCl_3$): δ 6.95 (s, 1H), 3.87 (s, 3H), 3.86 (s, 3H); ^{13}C NMR (126 MHz, $CDCl_3$): δ 152.19, 145.50, 128.91, 128.37, 123.38, 112.22, 60.85, 56.42. IR (ATR) *ν* (cm^{-1}) = 3011, 1632, 1615, 1595, 1578, 1483, 1468, 1432, 1410, 1373, 1332, 1272, 1211, 1128, 1035, 1010, 942, 912, 860, 809, 730, 672, 644. HRMS (m/z): $[M+H]^+$ calcd. for $C_8H_8Cl_3O_2$, 240.9584, found 240.9582. **8a:** ^{1}H NMR (500 MHz, $CDCl_3$): δ 9.86 (t, *J* = 1.1 Hz, 1H), 6.98 (s, 1H), 6.89 (s, 1H), 4.60 (d, *J* = 1.1 Hz, 2H), 3.88 (s, 3H); ^{13}C NMR (126 MHz, $CDCl_3$): δ 198.32, 149.01, 146.37, 125.79, 123.53, 116.52, 113.82, 77.24, 74.37, 56.36. IR (ATR) *ν* (cm^{-1}) = 3028, 2987, 2830, 1738, 1558, 1463, 1425, 1362, 1352,

1214, 1158, 1044, 923, 903, 877, 747, 712. HRMS (m/z): $[M+H]^+$ calcd. for $C_9H_9Cl_2O_3$, 234.9923, found 234.9926. **10a**: ^1H NMR (500 MHz, $CDCl_3$): δ7.01 (s, 1H), 6.90 (s, 1H), 5.57 (s, 1H), 3.88 (s, 3H); ^{13}C NMR (126 MHz, $CDCl_3$): δ 145.77, 144.92, 124.21, 122.68, 116.04, 112.33, 56.40. **11a**: ^1H NMR (500 MHz, $CDCl_3$): δ 6.91 (s, 1H), 5.91 (s, 1H), 3.92 (s, 3H). ^{13}C NMR (126 MHz, $CDCl_3$): δ 145.70, 142.14, 123.75, 123.72, 119.79, 110.74, 56.67. IR (ATR) *ν* (cm^{-1}) = 3425, 3410, 3210, 3045, 2917, 1583, 1498, 1460, 1440, 1408, 1343, 1269, 1203, 1120, 1031, 937, 904, 862, 802, 727, 678, 651. HRMS (m/z): $[M-H]^+$ calcd. for $C_7H_4Cl_3O_2$, 224.9276, found 224.9279.

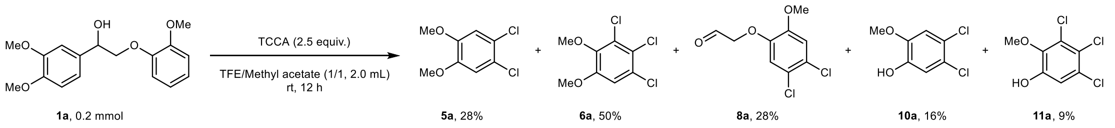

The reaction was conducted following the general procedure 5 in a 0.2 mmol scale for 12 h. The residue was purified by column chromatography on silica gel (300-400 mesh) with petroleum ether/ethyl acetate (10:1) to afford the product **5a** as a white solid (11.59 mg, 28% yield), **6a** as a white solid (24.15 mg, 50%), and on silica gel with petroleum ether/ethyl acetate (5:1) to afford **8a** as a white solid (13.16 mg, 28% yield). **10a** and **11a** were determined by GC-MS and isolated by preparative thin-layer chromatography (PTLC) with petroleum ether / ethyl acetate (3:1).

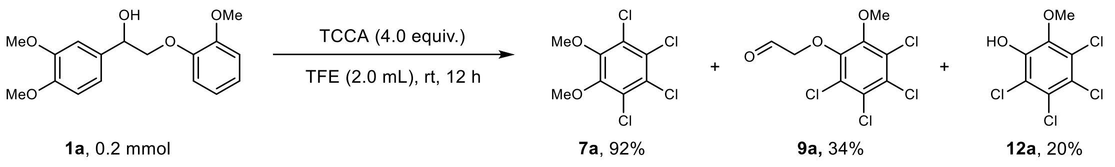

The reaction was conducted following the general procedure 6 in a 0.2 mmol scale for 12 h. The residue was purified by column chromatography on silica gel (300-400 mesh) with petroleum ether/ethyl acetate (10:1) to afford the product **7a** as a white solid (50.77 mg, 92% yield) and on silica gel with petroleum ether / ethyl acetate (5:1) to afford **9a** as a white solid (20.66 mg, 34% yield), and on silica gel with petroleum

ether/ethyl acetate (3:1) to give **12a** as a white solid (10.48 mg, 20% yield)[25]. **7a:** ^{1}H NMR (500 MHz, $CDCl_3$): δ 3.92 (s, 6H); ^{13}C NMR (126 MHz, $CDCl_3$): δ 149.87, 128.20, 127.55, 61.30. IR (ATR) *v* (cm^{-1}) = 2883, 1782, 1763, 1702, 1655, 1600, 1547, 1453, 1440, 1407, 1375, 1310, 1228, 1175, 1134, 1032, 970, 935, 819, 750, 720, 715, 700, 671, 520. HRMS (m/z): $[M+H]^+$ calcd. for $C_8H_7Cl_4O_2$, 276.9165, found 276.9163. **9a:** ^{1}H NMR (500 MHz, $CDCl_3$): δ 9.92 (t, *J* = 1.1 Hz, 1H), 4.64 (d, *J* = 1.0 Hz, 2H), 3.92 (s, 3H); ^{13}C NMR (126 MHz, $CDCl_3$): δ 197.67, 149.33, 148.38, 129.20, 128.78, 127.95, 127.15, 77.77, 61.64. IR (ATR) *v* (cm^{-1}) = 2837, 1794, 1747, 1728, 1600, 1538, 1510, 1453, 1403, 1369, 1350, 1222, 1072, 1023, 976, 909, 882, 817, 743, 731, 722, 702. HRMS (m/z): $[M+H]^+$ calcd. for $C_9H_7Cl_4O_3$, 304.9114, found 304.9114. **12a:** ^{1}H NMR (500 MHz, $CDCl_3$): δ 6.06 (s, 1H), 3.95 (s, 3H); ^{13}C NMR (126 MHz, $CDCl_3$): δ 145.85, 143.37, 128.35, 126.40, 124.03, 119.35, 61.36.

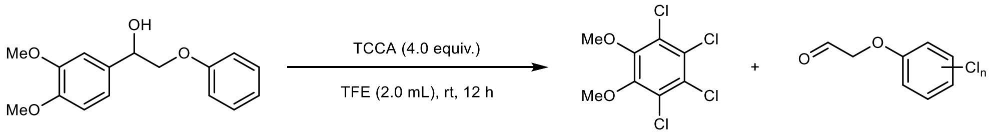

1b, 0.2 mmol **6a**, 74% mixture

The reaction was conducted following the general procedure 6 in a 0.2 mmol scale for 12 h. The residue was purified by column chromatography on silica gel (300-400 mesh) with petroleum ether/ethyl acetate (10:1) to afford the product **6a** as a white solid (40.83 mg, 74% yield), and the right part yielded a mixture of di-chlorinated and tri-chlorinated products (Supplementary Figure 5).

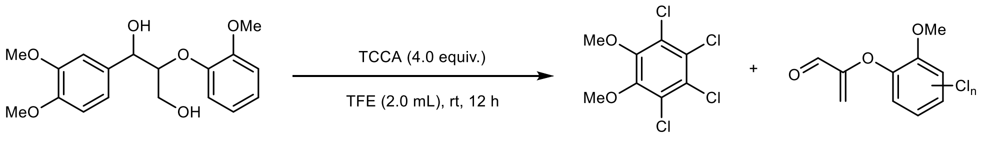

1c, 0.2 mmol **6a**, 62% mixture

The reaction was conducted following the general procedure 6 in a 0.2 mmol scale for 12 h. The residue was purified by column chromatography on silica gel (300-400 mesh) with petroleum ether/ethyl acetate (10:1) to afford the product **6a** as a white

solid (34.21 mg, 62% yield), and the right part yielded a mixture of tri-chlorinated and tetra-chlorinated products (Supplementary Figure 6).

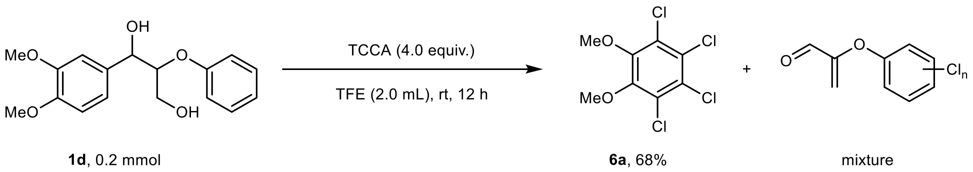

The reaction was conducted following the general procedure 6 in a 0.2 mmol scale for 12 h. The residue was purified by column chromatography on silica gel (300-400 mesh) with petroleum ether/ethyl acetate (10:1) to afford the product **6a** as a white solid (37.52 mg, 68% yield), and the right part yielded a mixture of di-chlorinated and tri-chlorinated products (Supplementary Figure 7).

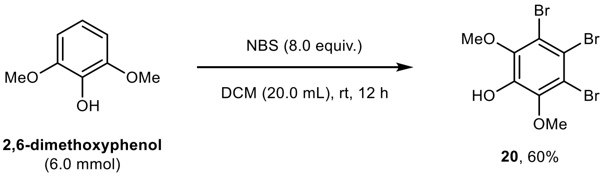

To a stirred solution of 2,6-dimethoxyphenol (0.92 g, 6.0 mmol, 1.0 equiv.) in CH_2Cl_2 (20 mL), NBS (8.54 g, 48.0 mmol, 8.0 equiv.) was added. After the mixture was stirred for 12 h at 25°C, the residue was purified by column chromatography on silica gel (300-400 mesh) with petroleum ether/ethyl acetate (10:1) to afford the product 3,4,5-tribromo-2,6-dimethoxyphenol **20** as a white solid (1.40 g, 60% yield)[22]. ^{1}H NMR (500 MHz, $CDCl_3$): δ 5.78 (s, 1H), 3.92 (s, 6H); ^{13}C NMR (126 MHz, $CDCl_3$): δ 145.62, 142.92, 117.69, 116.23, 60.98.

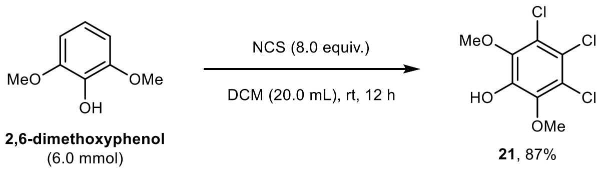

To a stirred solution of 2,6-dimethoxyphenol (0.92 g, 6.0 mmol, 1.0 equiv.) in CH_2Cl_2 (20 mL), NCS (6.40 g, 48.0 mmol, 8.0 equiv.) was added. After the mixture was

stirred for 12 h at 25°C, the residue was purified by column chromatography on silica gel (300-400 mesh) with petroleum ether/ethyl acetate (10:1) to afford the product 3,4,5-trichloro-2,6-dimethoxyphenol **21** as a white solid (1.34 g, 87% yield). ^{1}H NMR (500 MHz, $CDCl_3$): δ 5.79 (s, 1H), 3.94 (s, 6H); ^{13}C NMR (126 MHz, $CDCl_3$): δ 143.63, 142.63, 123.40, 122.88, 61.19. IR (ATR) *v* (cm^{-1}) = 3411, 3290, 2937, 1603, 1560, 1468, 1443, 1422, 1397, 1310, 1217, 1189, 1075, 1000, 971, 926, 830, 780, 745, 721, 711, 696, 653, 556, 456. HRMS (m/z): $[M+H]^+$ calcd. for $C_8H_8Cl_3O_3$, 256.9533, found 256.9530.

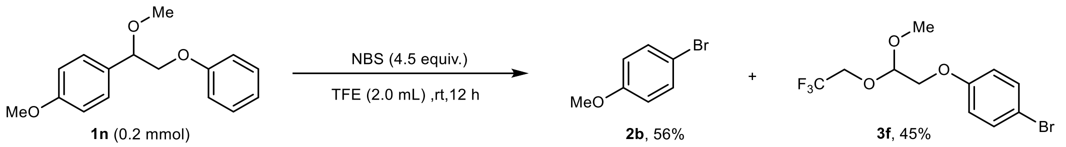

1n (0.2 mmol) **2b**, 56% **3f**, 45%

To a stirred solution of **1n** (51.66 mg, 0.2 mmol, 1.0 equiv.) in TFE (2 mL), NBS (160.19 mg, 0.9 mmol, 4.5 equiv.) was added. After the mixture was stirred for 12 h at 25 °C, the residue was purified by column chromatography on silica gel (300-400 mesh) with petroleum ether/ethyl acetate (10:1) to afford the products 1-bromo-4-methoxybenzene **2b** (56% yield) and 1-bromo-4-(2-methoxy-2-(2,2,2-trifluoroethoxy)ethoxy)benzene **3f** (45% yield). **3f**: ^{1}H NMR (500 MHz, $CDCl_3$): δ 7.40-7.37 (m, 2H), 6.81-6.78 (m, 2H), 4.91 (t, J = 5.5 Hz, 1H), 4.05-3.96 (m, 4H), 3.48 (s, 3H); ^{13}C NMR (126 MHz, $CDCl_3$): δ 157.25, 132.36, 123.75 116.38, 113.66 (q, $J_{C\text{-}F}$ = 278.59 Hz), 101.35, 67.60, 63.15 (q, $J_{C\text{-}F}$ = 35.28 Hz), 54.45. IR (ATR) *v* (cm^{-1}) = 3020, 2910, 1592, 1489, 1458, 1350, 1302, 1283, 1245, 1163, 1144, 1086, 1071, 1049, 1005, 965, 901, 821, 716, 641. HRMS (m/z): $[M+Na]^+$ calcd. for $C_{11}H_{12}BrF_3NaO_3$, 350.9814, found 350.9809.

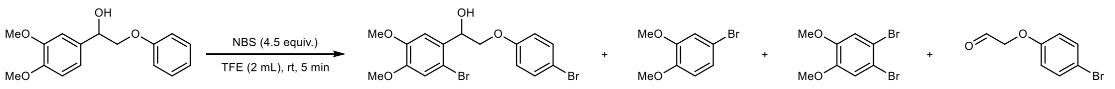

1b, 0.2 mmol **1o**, ^{1}H NMR, ^{13}C NMR detected **14**, GC-MS detected **2a**, GC-MS detected **3b**, GC-MS detected

To a stirred solution of **1b** (54.86 mg, 0.2 mmol, 1.0 equiv.) in TFE (2 mL), NBS (160.19 mg, 0.9 mmol, 4.5 equiv.) was added. The mixture was stirred for 5 min at 25°C, the residue was firstly detected by GC-MS (see Supplementary Figure 27) and

then purified by column chromatography on silica gel (300-400 mesh) with petroleum ether/ethyl acetate (10:1) to afford the product **1o** as a white solid (2% yield). **1o:** ^{1}H NMR (500 MHz, $CDCl_3$): δ 7.40-7.37 (m, 2H), 7.19 (s, 1H), 7.01 (s, 1H), 6.85-6.82 (m, 2H), 5.39 (dd, *J* = 8.5, 2.0 Hz, 1H), 4.18 (dd, *J* = 10.0, 3.0 Hz, 1H), 3.91(s, 3H), 3.88 (s, 3H), 3.81 (t, *J* = 9.0 Hz, 1H); ^{13}C NMR (126 MHz, $CDCl_3$): δ 157.39, 149.27, 148.85, 132.37, 130.27, 116.48, 115.23, 113.55, 111.73, 110.23, 71.87, 71.25, 56.20, 56.08. IR (ATR) *ν* (cm^{-1}) = 3416, 3400, 3257, 3104, 3008, 2963, 1602, 1591, 1576, 1514, 1490, 1460, 1444, 1381, 1284, 1260, 1243, 1223, 1201, 1154, 1099, 1036, 1015, 968, 911, 867, 839, 825, 798, 742, 642, 589, 553. HRMS (m/z): $[M+Na]^+$ calcd. for $C_{16}H_{16}Br_2NaO_4$, 454.9287, found 454.9282.

NMR Spectra

^{1}H NMR (500 MHz, $CDCl_3$)

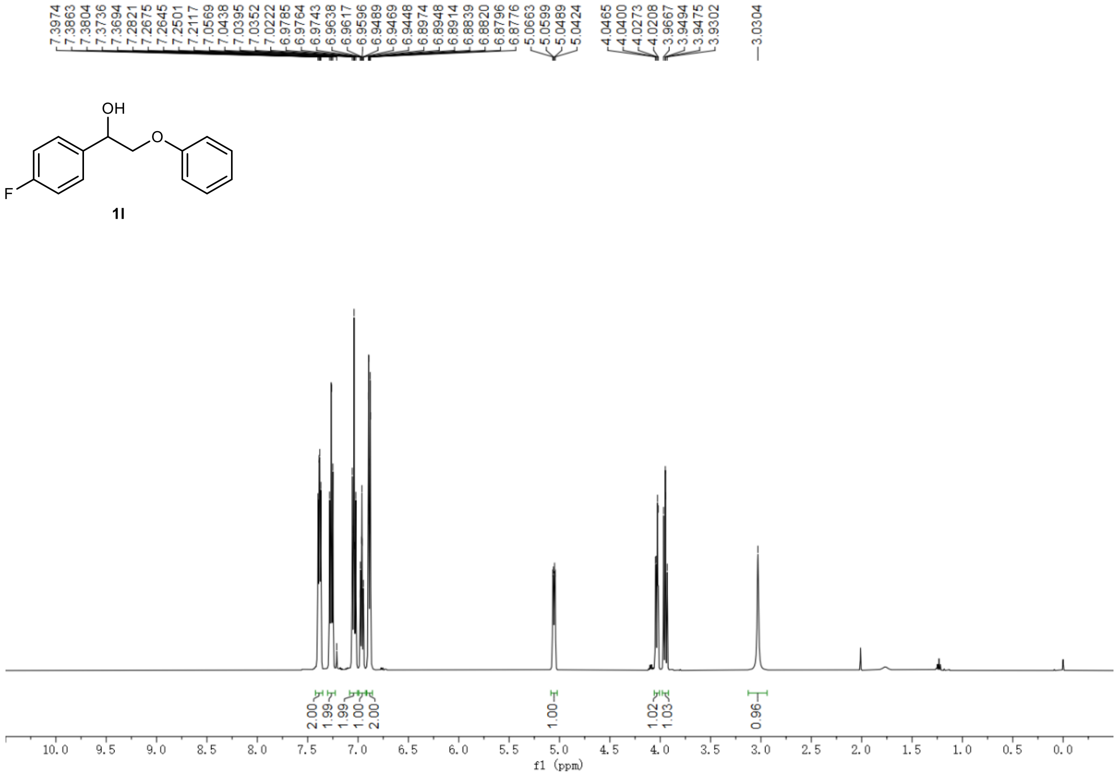

^{13}C NMR (126 MHz, $CDCl_3$)

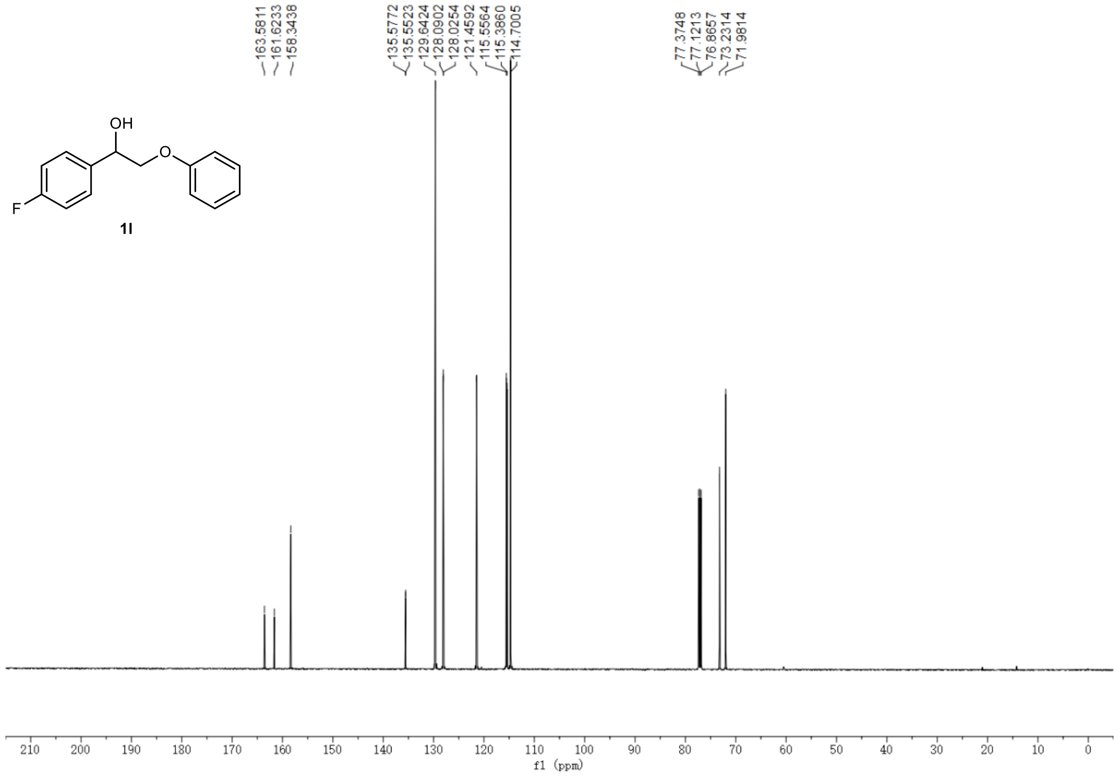

^{1}H NMR (500 MHz, $CDCl_3$)

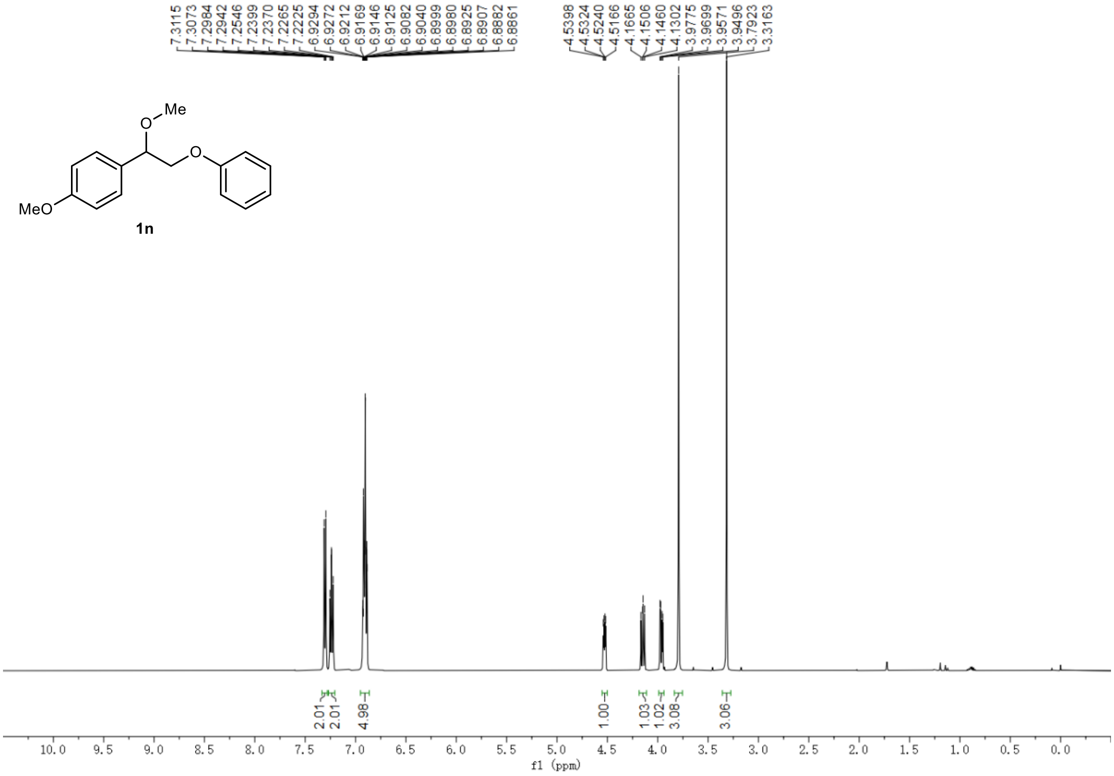

^{13}C NMR (126 MHz, $CDCl_3$)

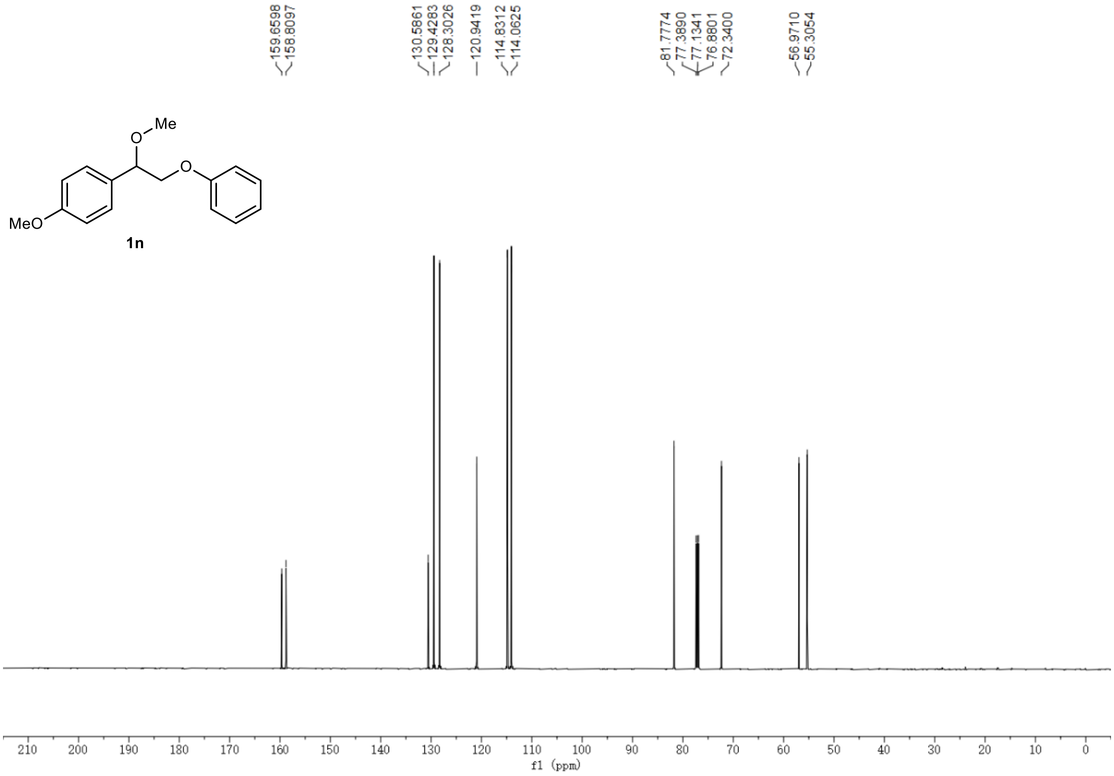

^{1}H NMR (500 MHz, $CDCl_3$)

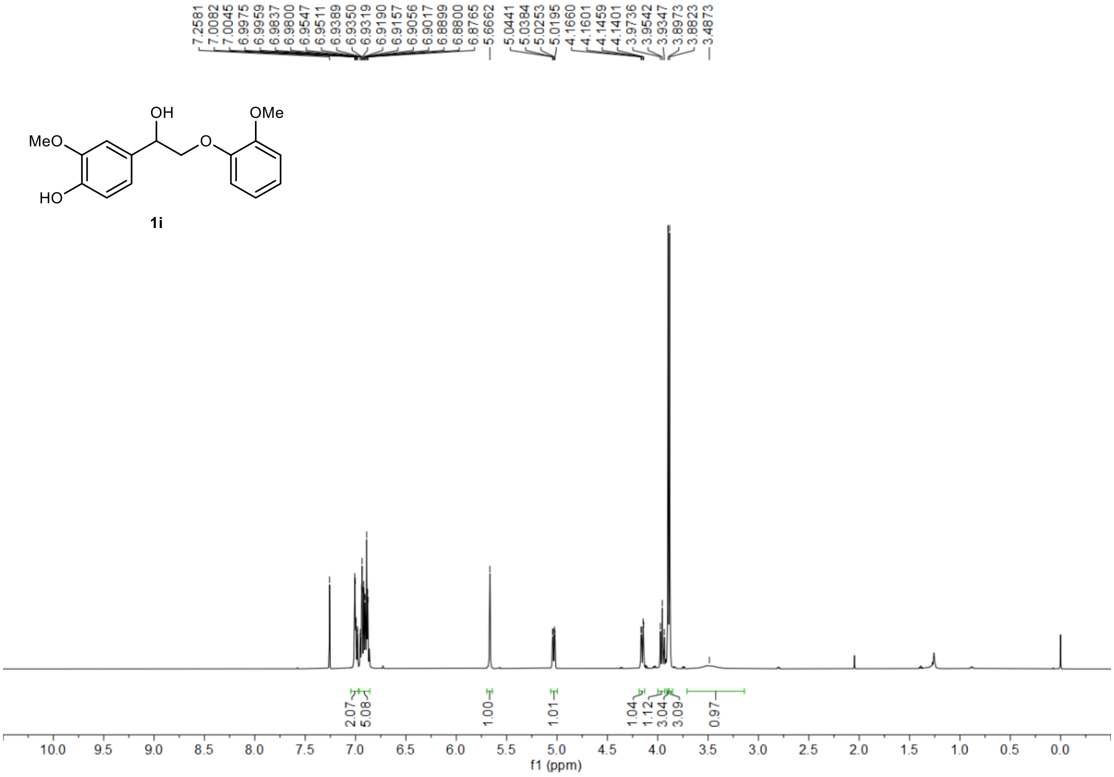

^{13}C NMR (126 MHz, $CDCl_3$)

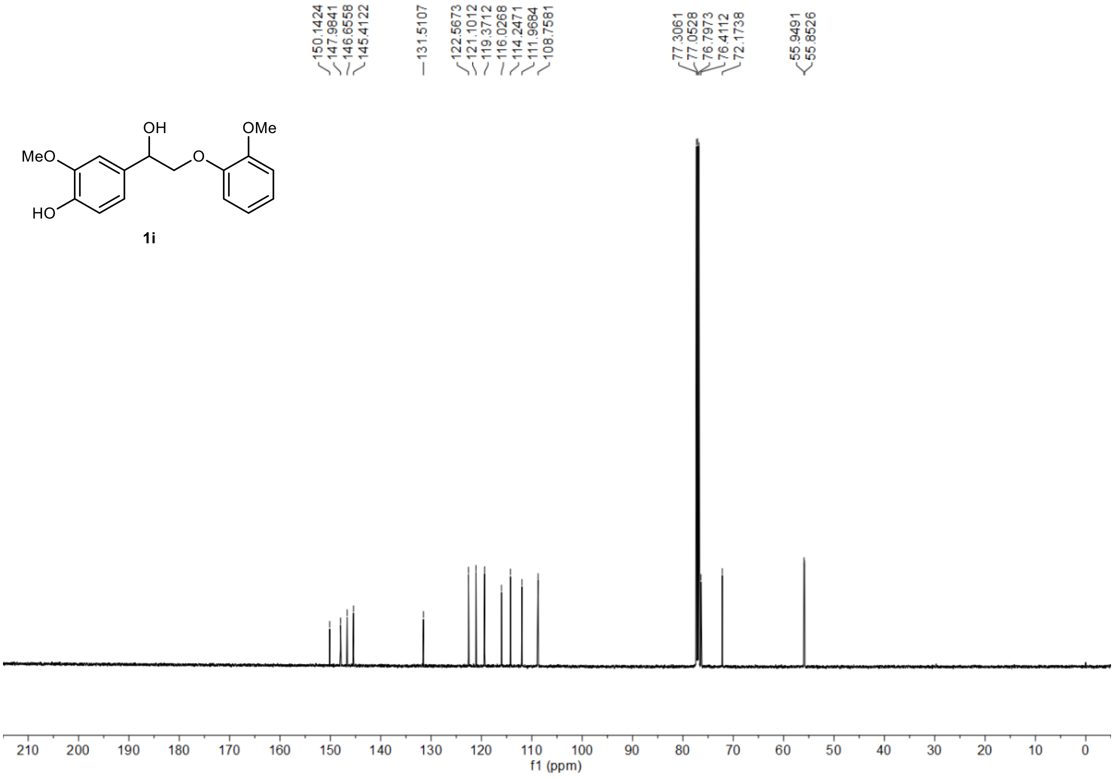

^{1}H NMR (500 MHz, $CDCl_3$)

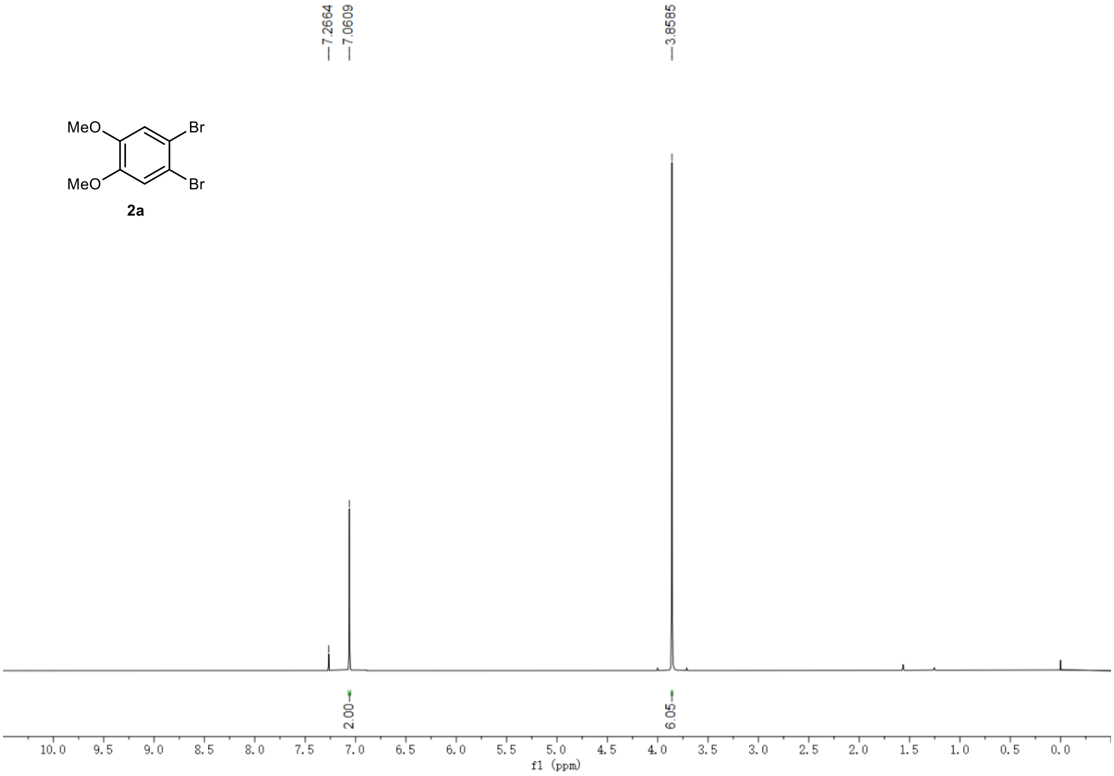

^{13}C NMR (126 MHz, $CDCl_3$)

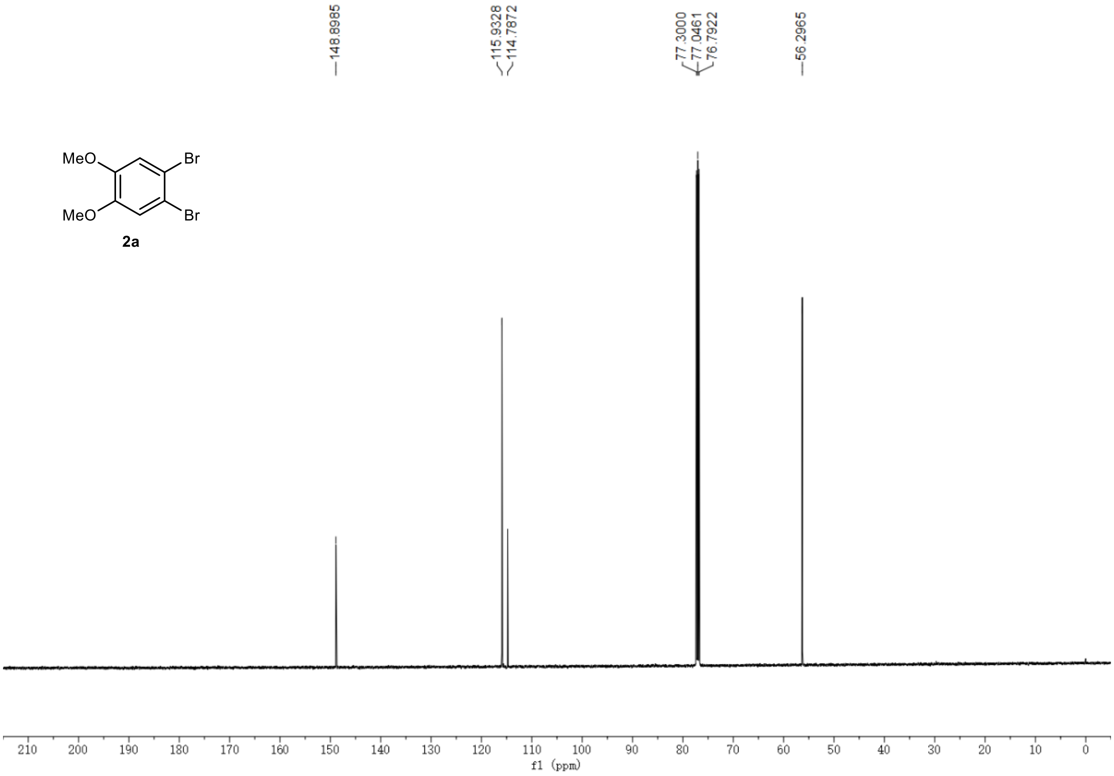

^{1}H NMR (500 MHz, $CDCl_3$)

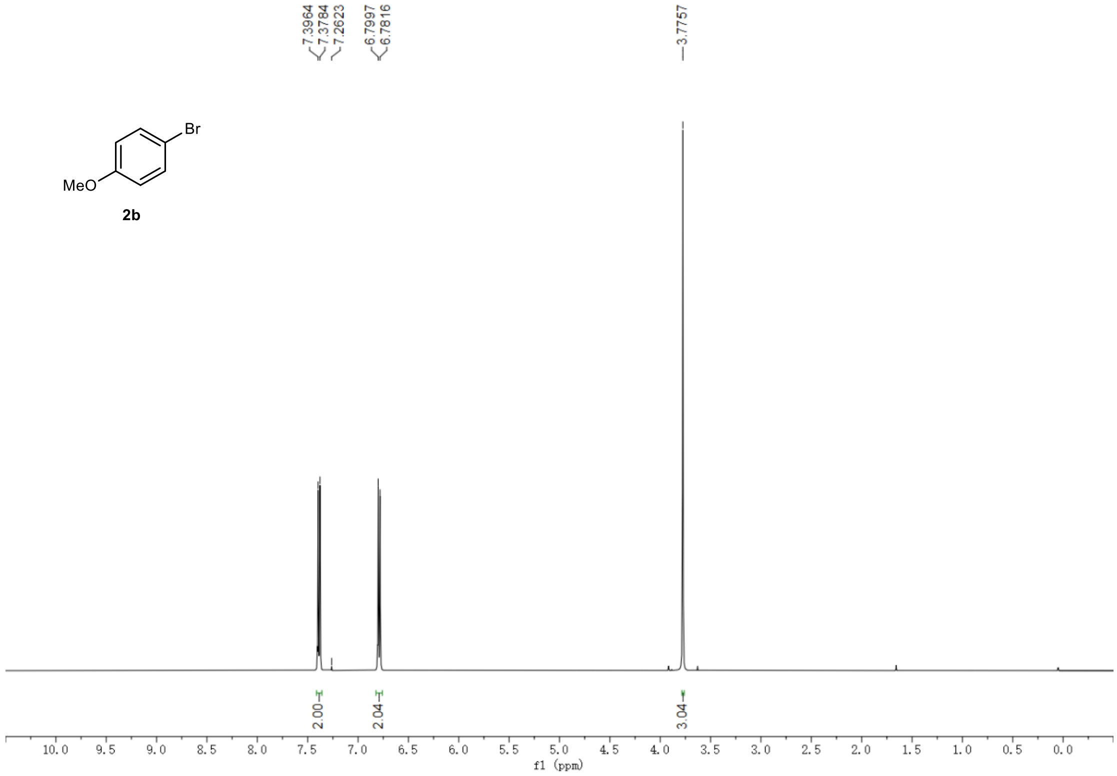

^{13}C NMR (126 MHz, $CDCl_3$)

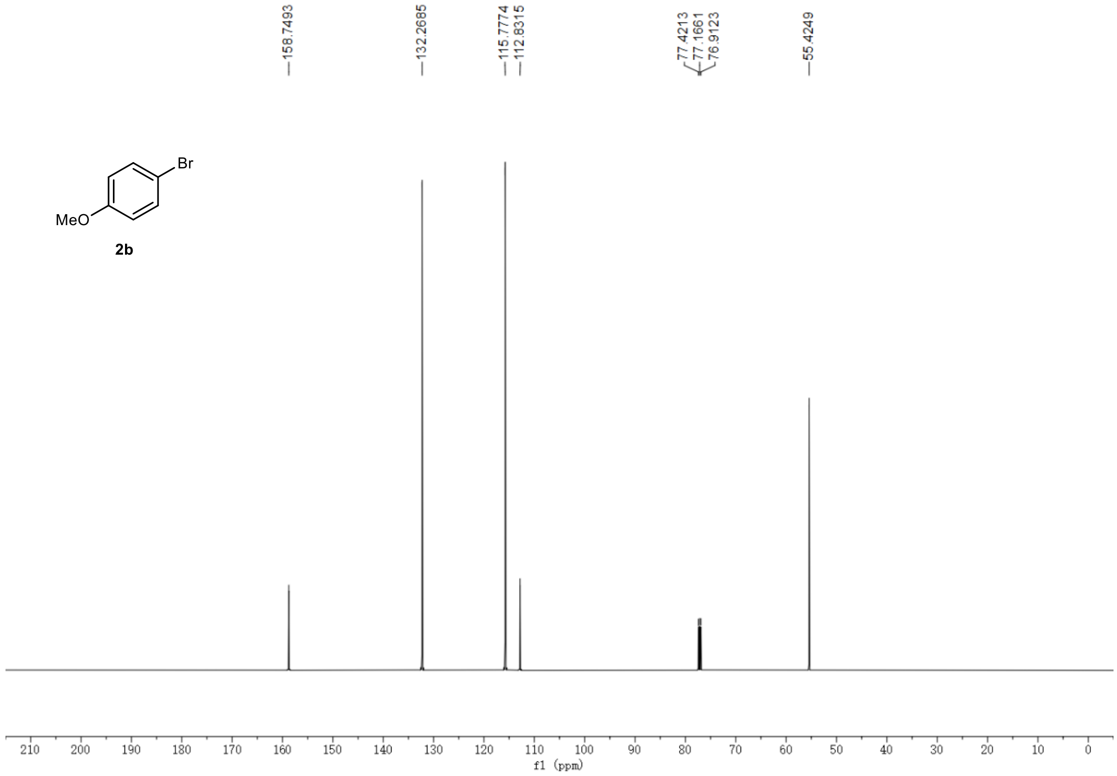

^{1}H NMR (500 MHz, $CDCl_3$)

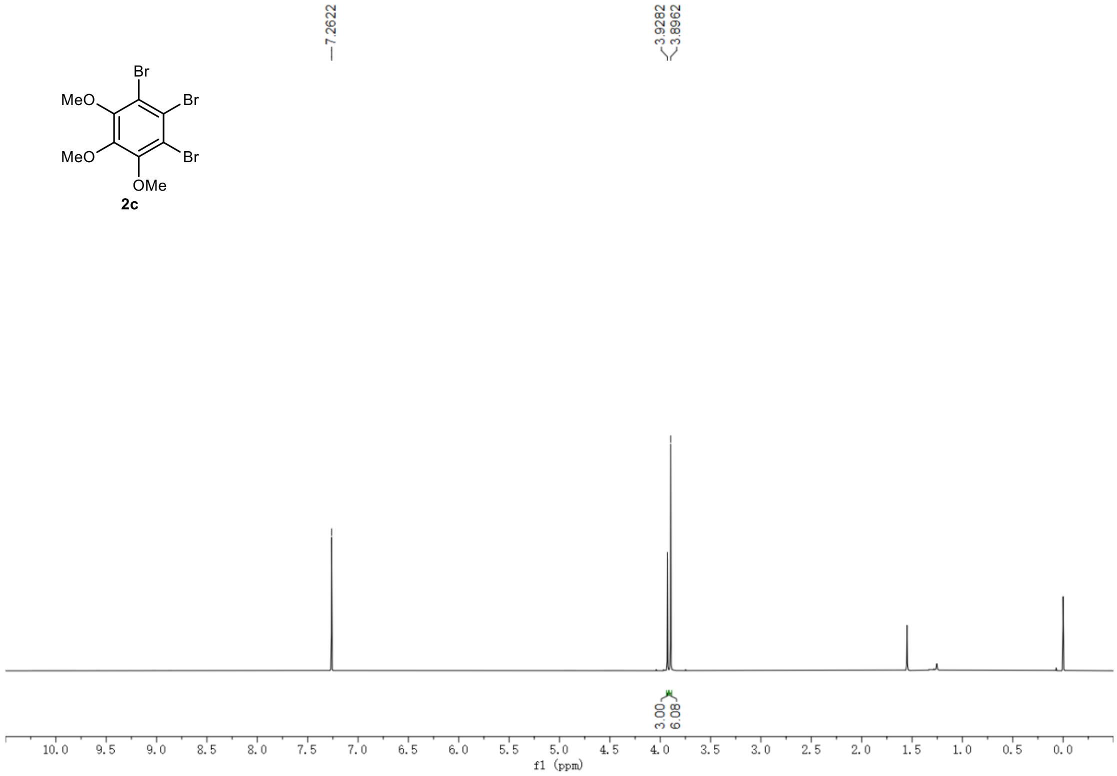

^{13}C NMR (126 MHz, $CDCl_3$)

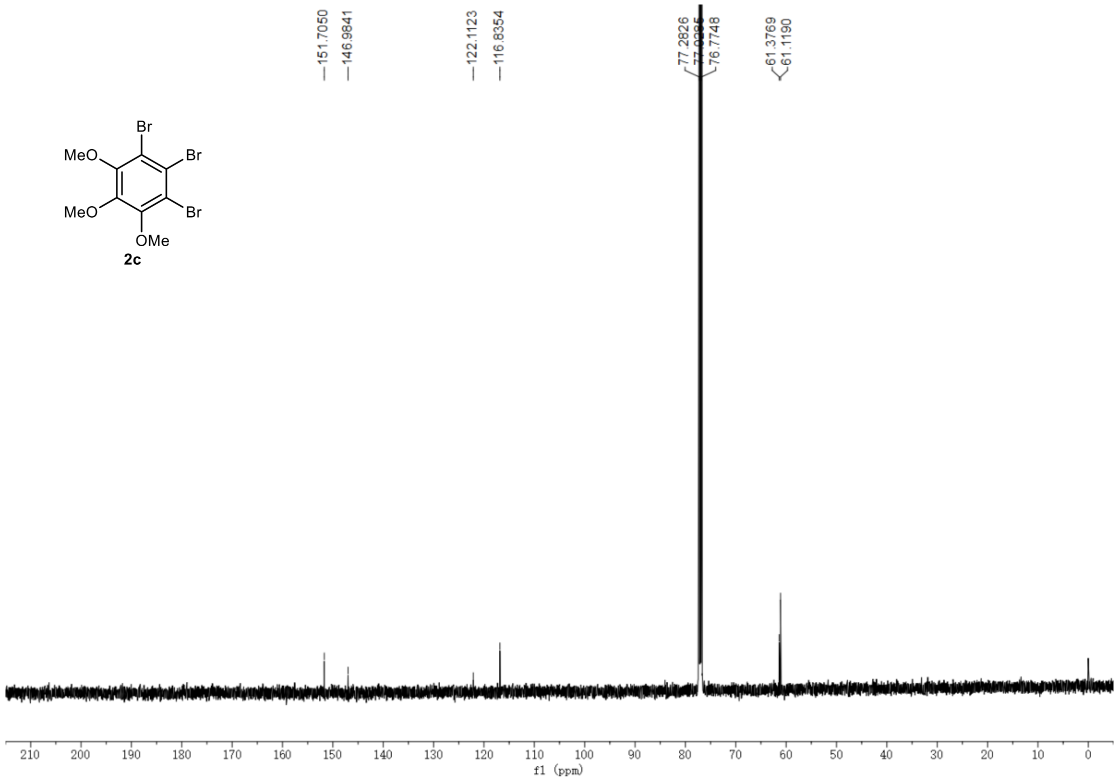

^{1}H NMR (500 MHz, $CDCl_3$)

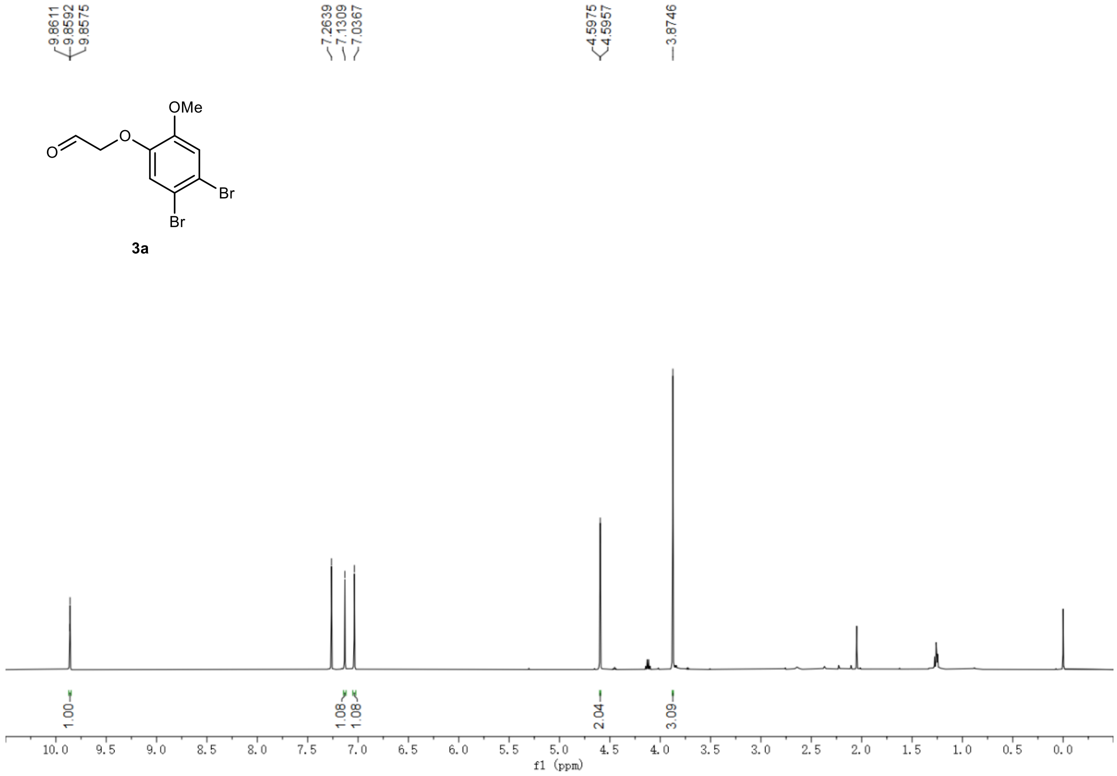

^{13}C NMR (126 MHz, $CDCl_3$)

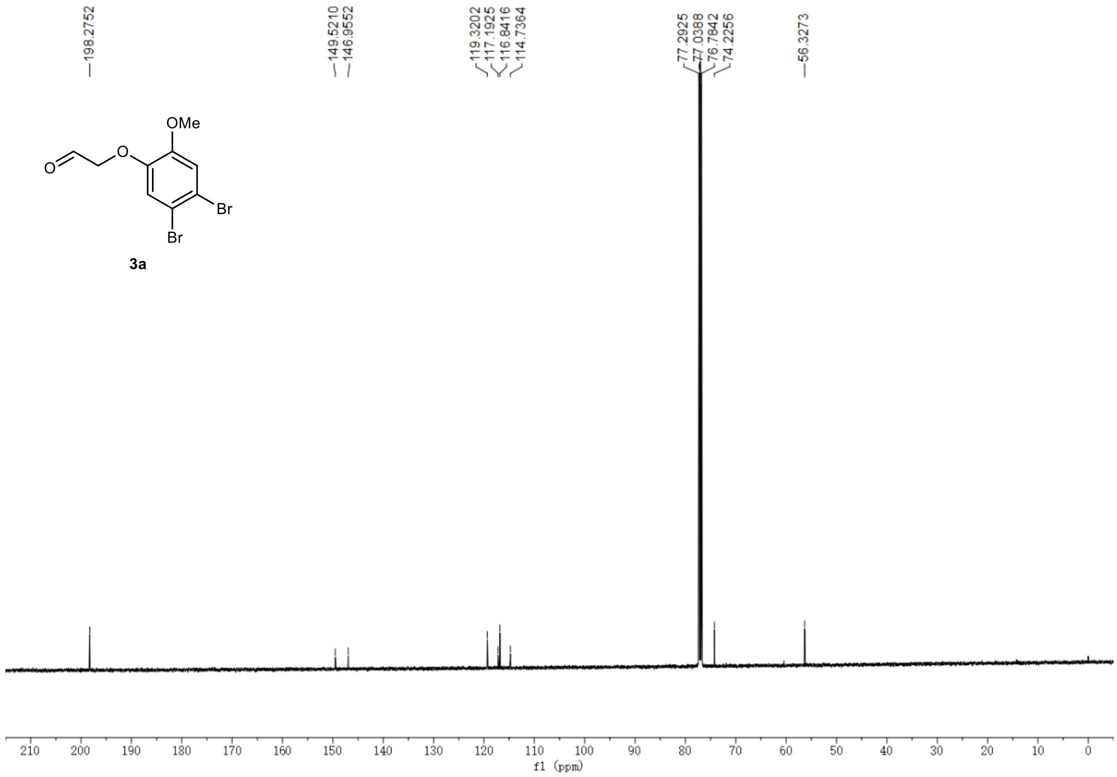

^{1}H NMR (500 MHz, $CDCl_3$)

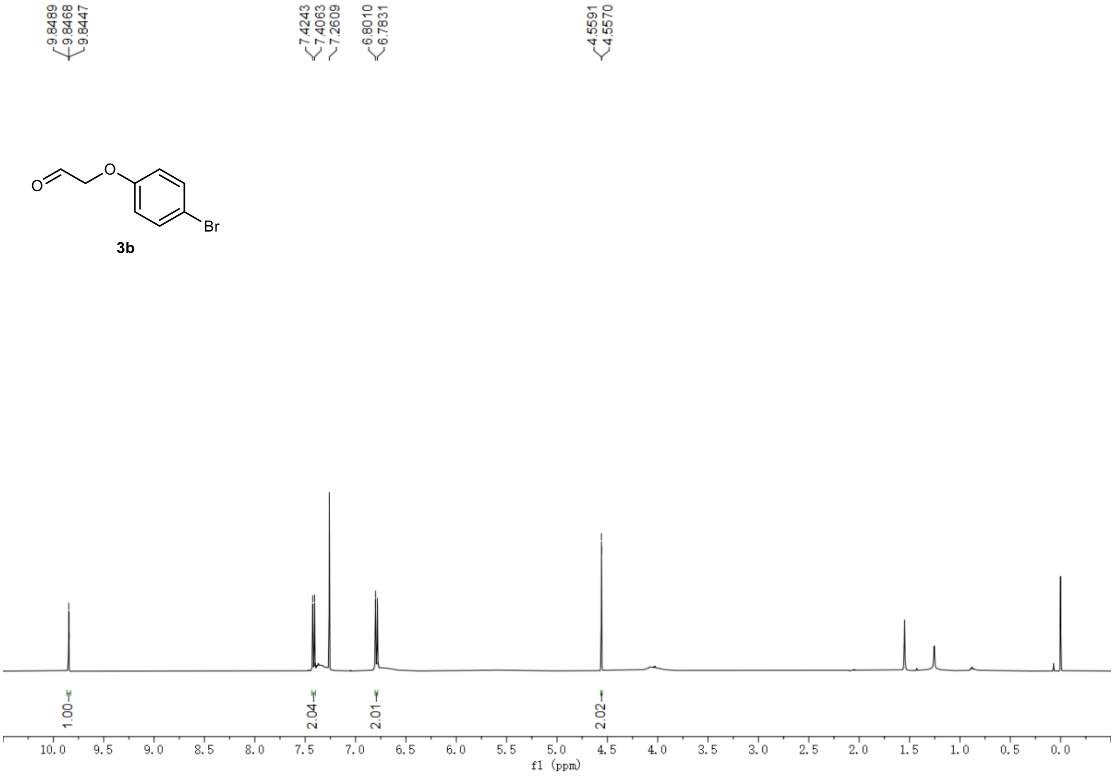

^{13}C NMR (126 MHz, $CDCl_3$)

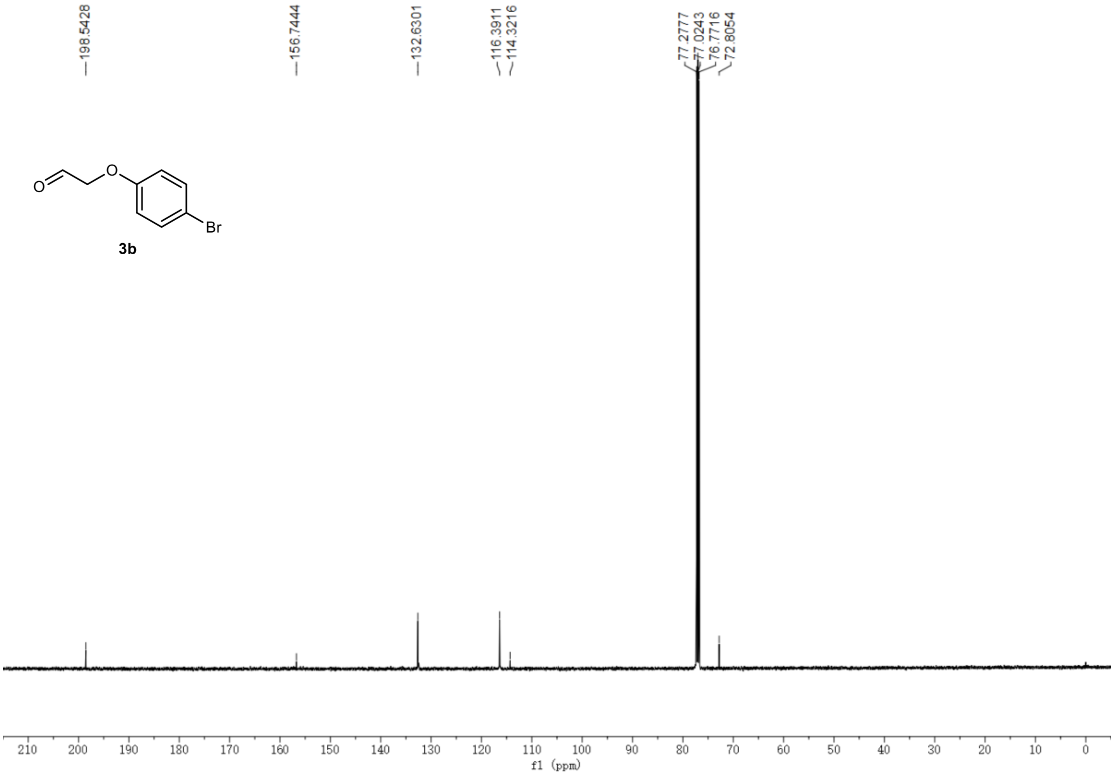

^{1}H NMR (500 MHz, $CDCl_3$)

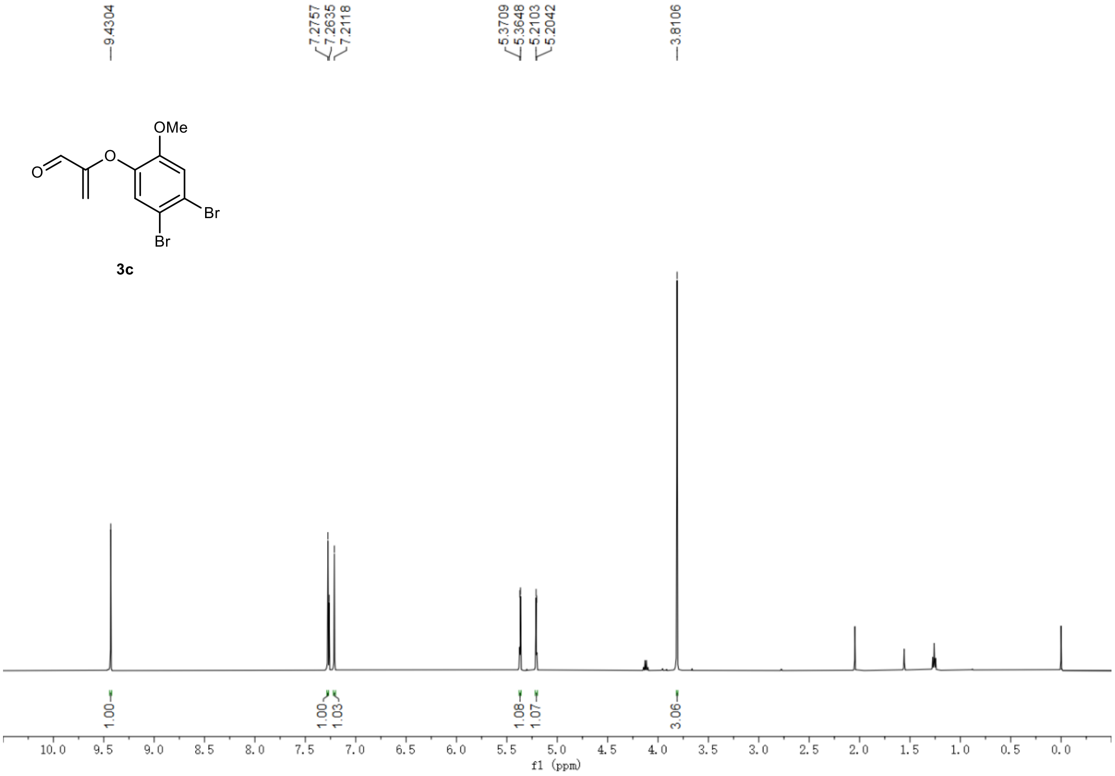

^{13}C NMR (126 MHz, $CDCl_3$)

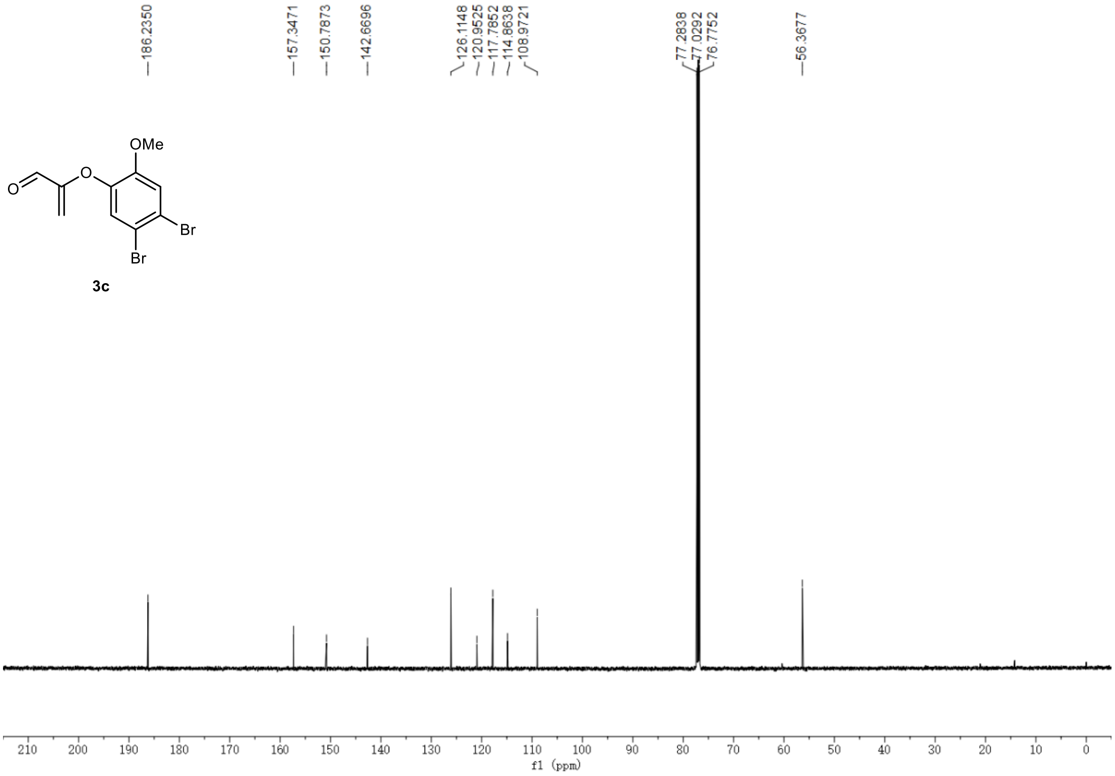

^{1}H NMR (500 MHz, $CDCl_3$)

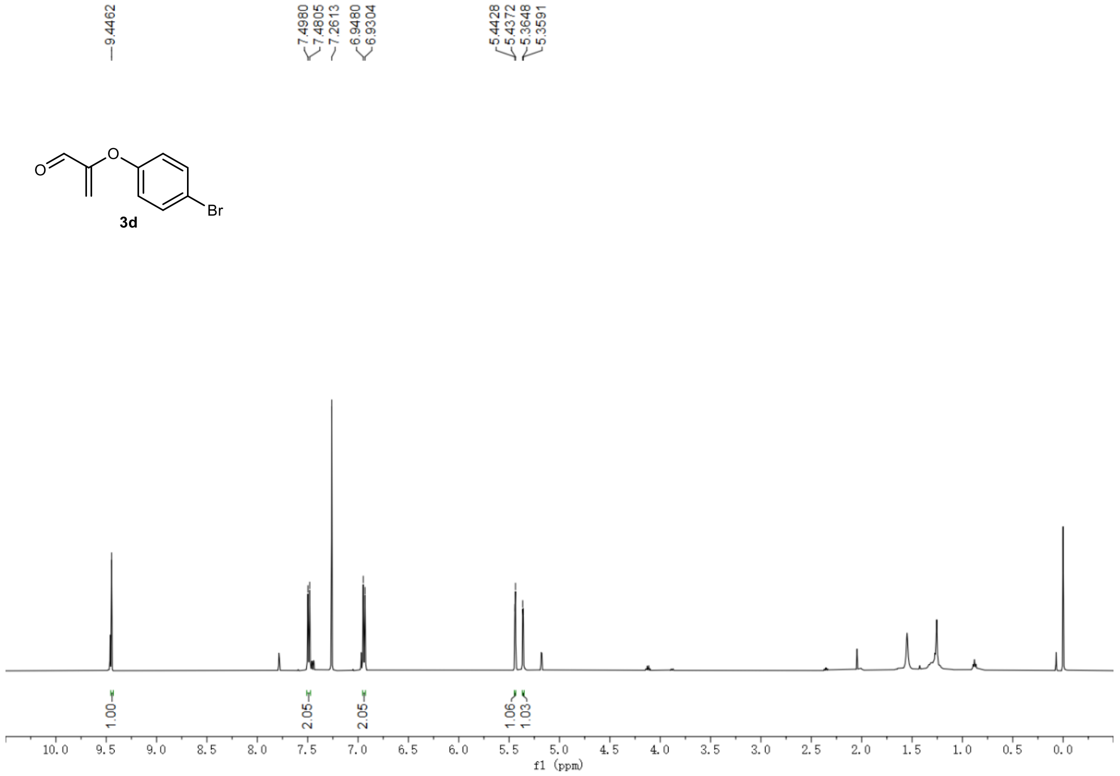

^{13}C NMR (126 MHz, $CDCl_3$)

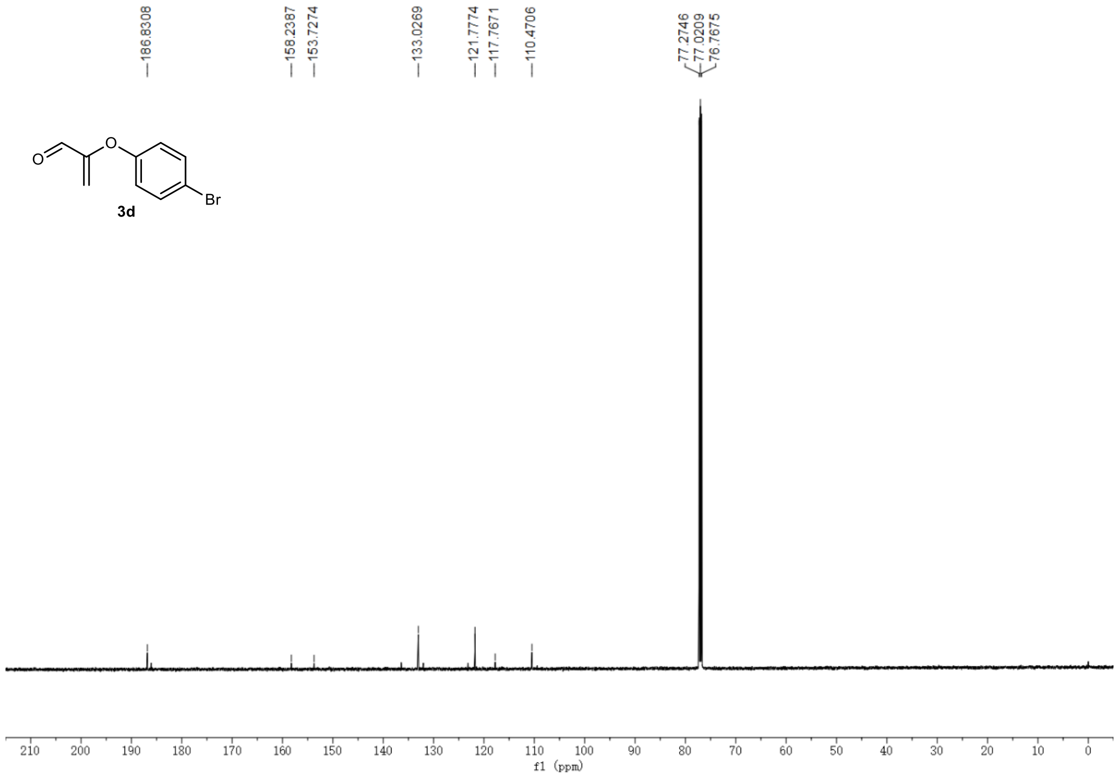

^{1}H NMR (500 MHz, $CDCl_3$)

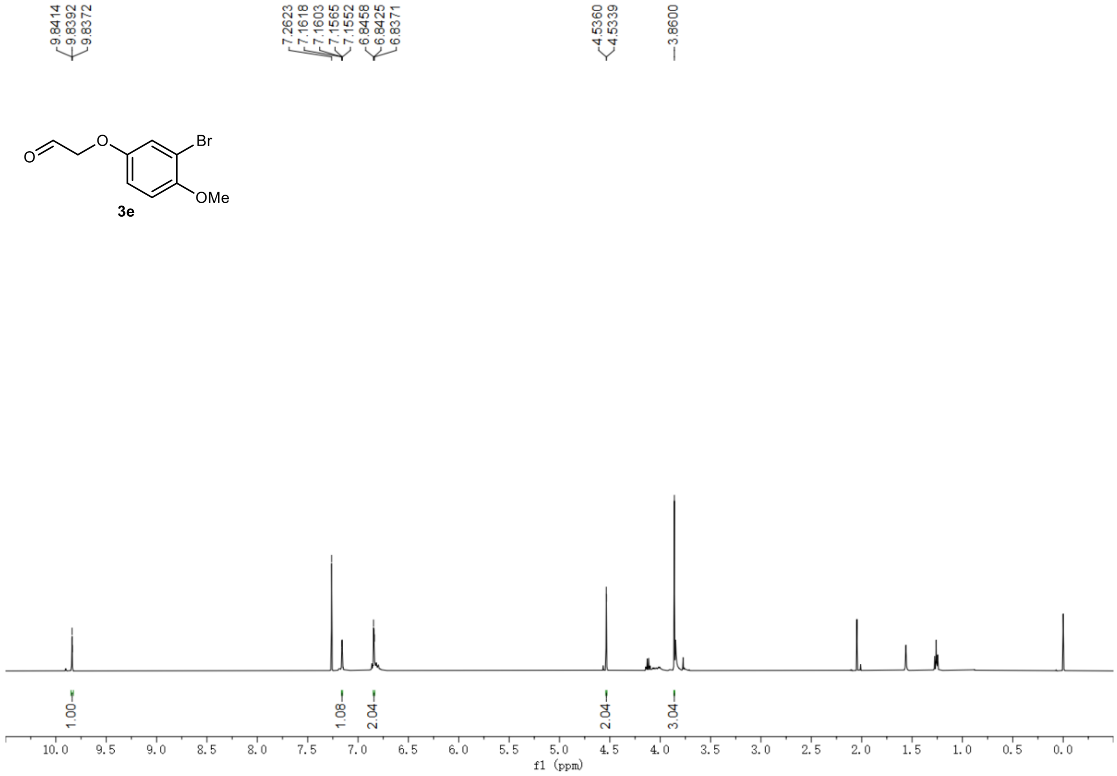

^{13}C NMR (126 MHz, $CDCl_3$)

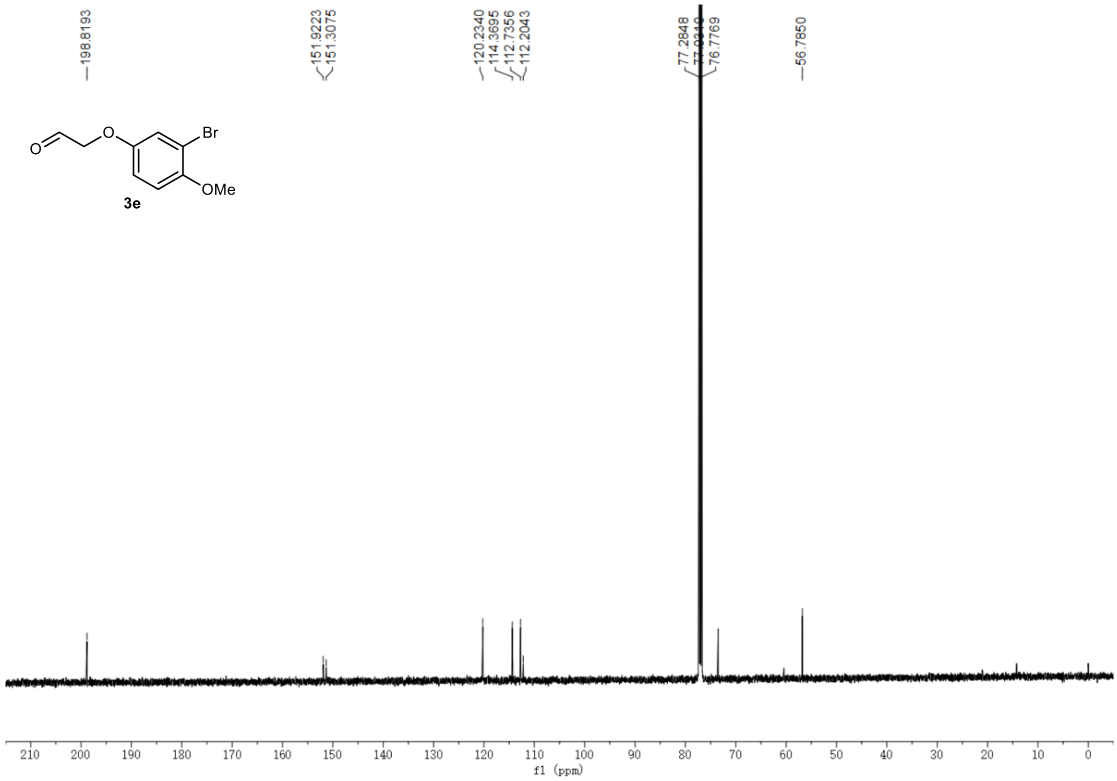

^{1}H NMR (500 MHz, $CDCl_3$)

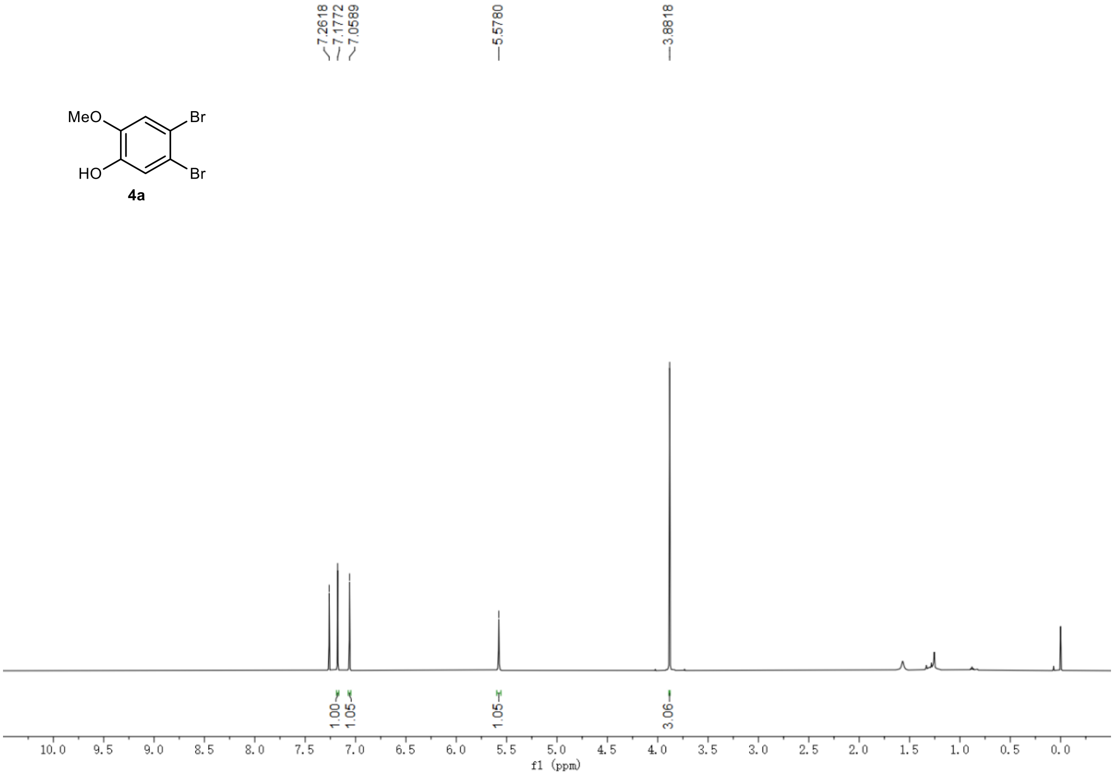

^{13}C NMR (126 MHz, $CDCl_3$)

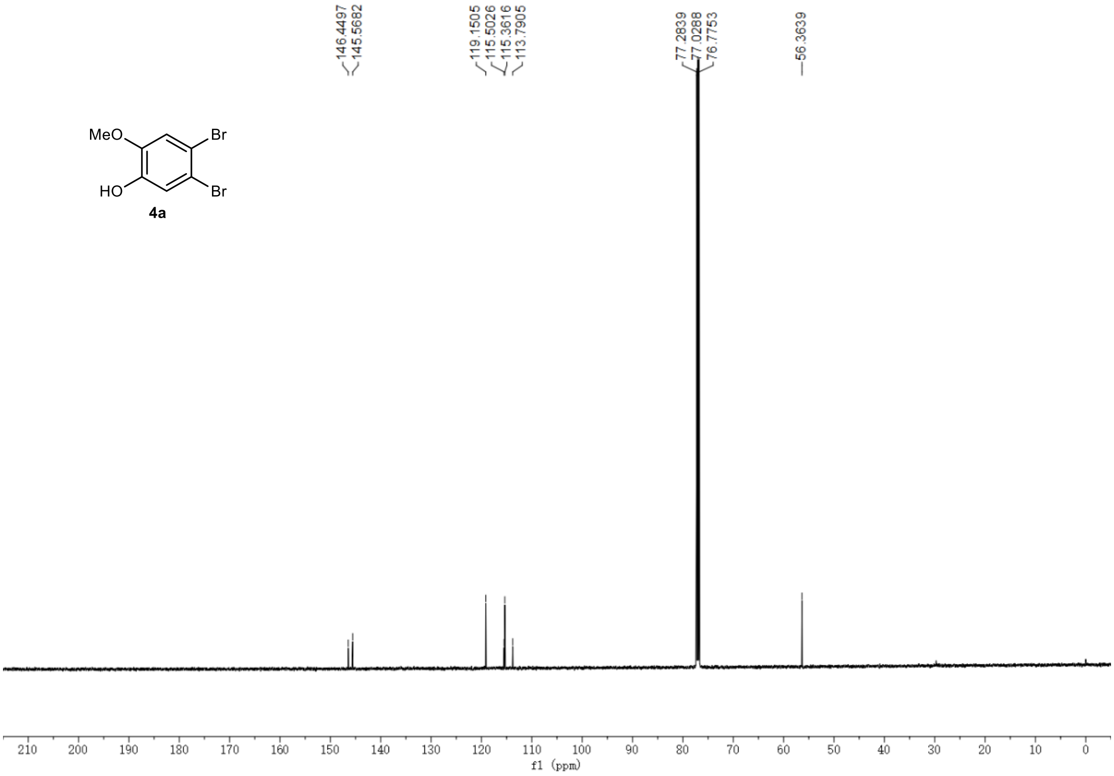

^{1}H NMR (500 MHz, $CDCl_3$)

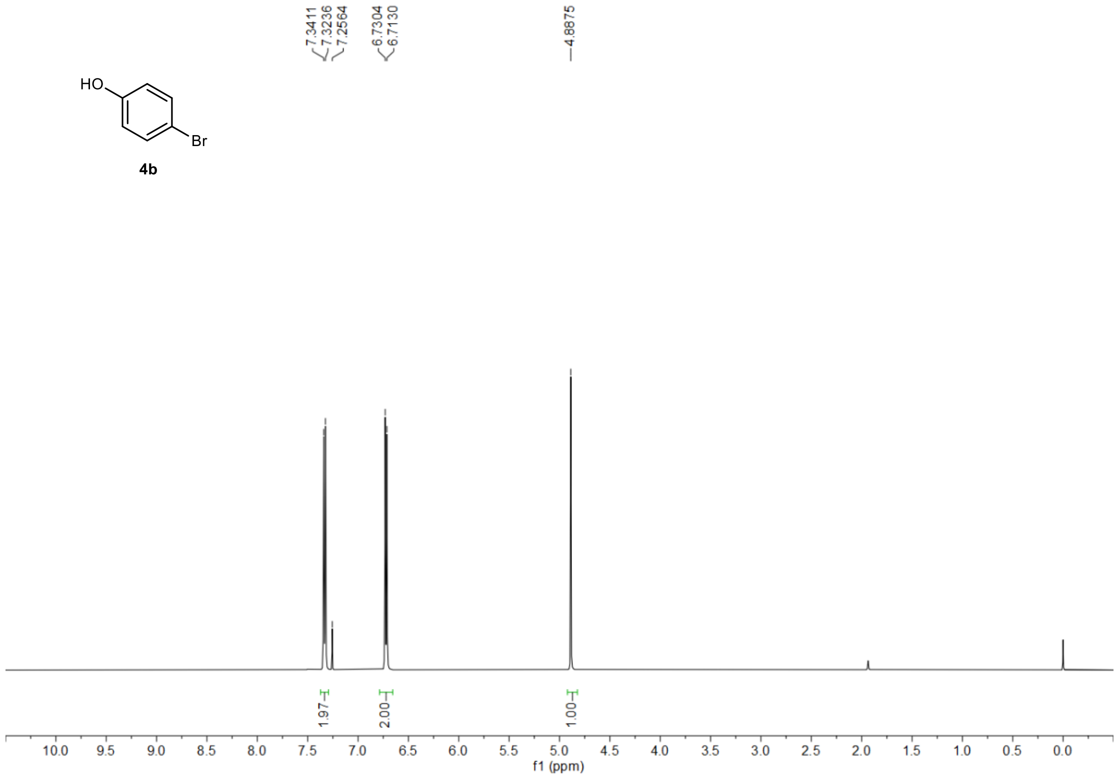

^{13}C NMR (126 MHz, $CDCl_3$)

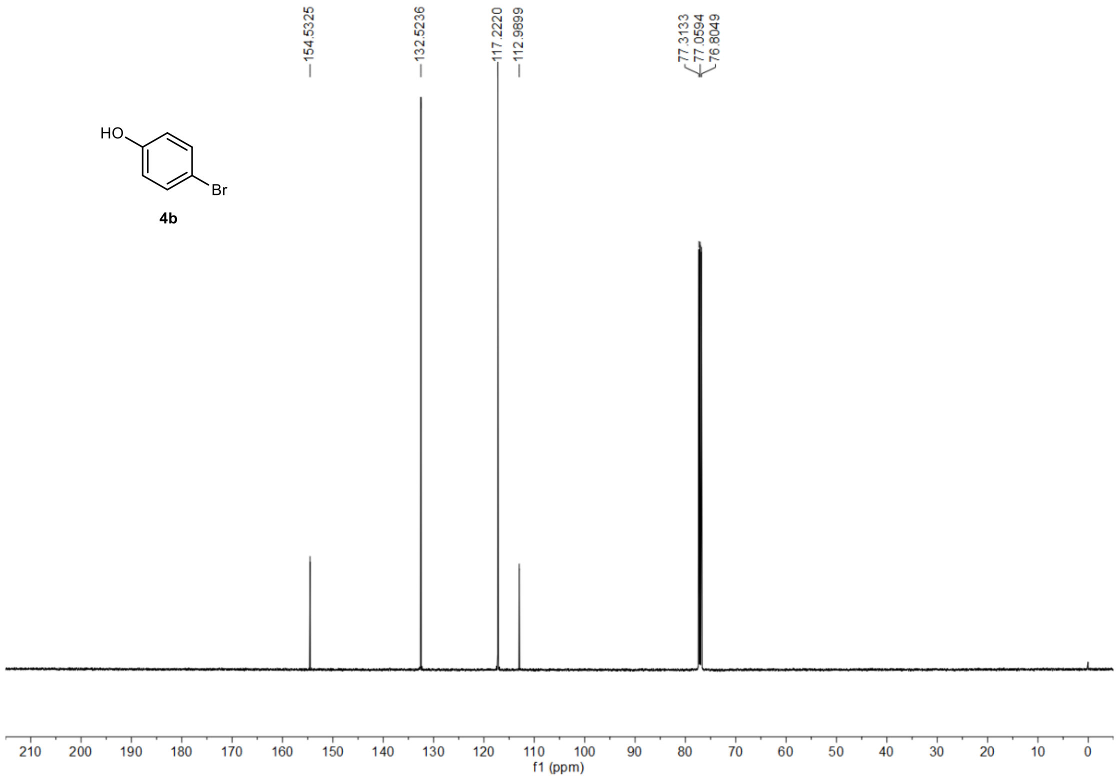

^{1}H NMR (500 MHz, $CDCl_3$)

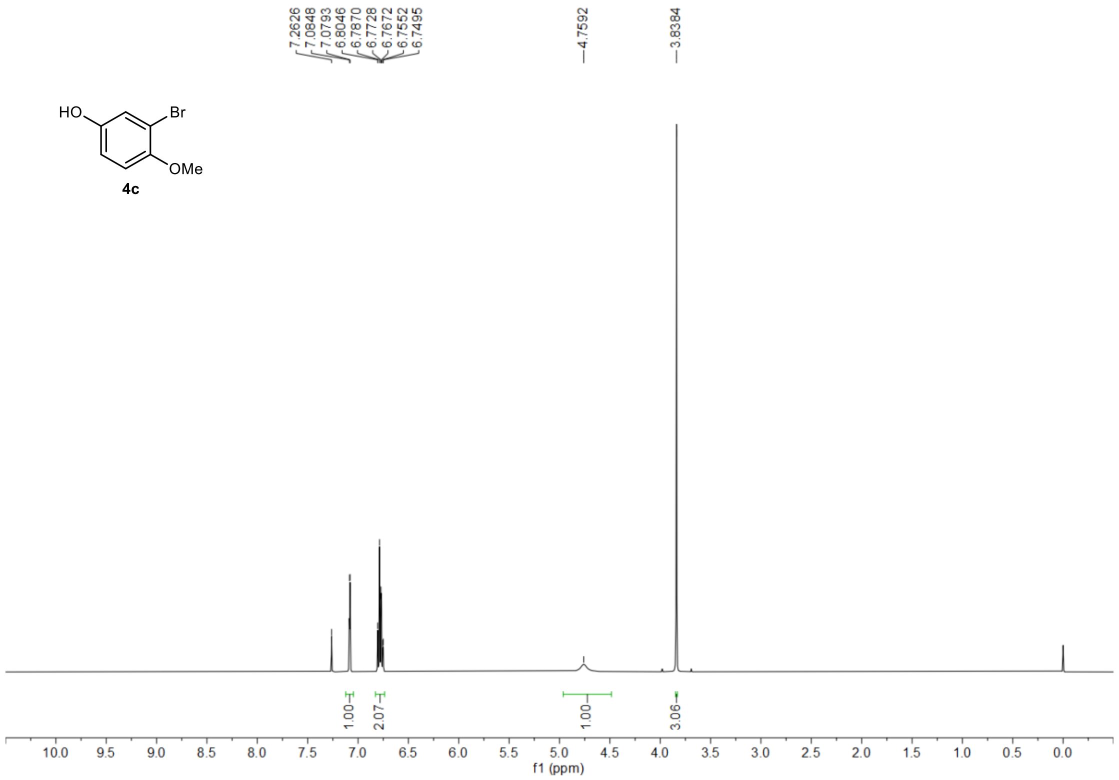

^{13}C NMR (126 MHz, $CDCl_3$)

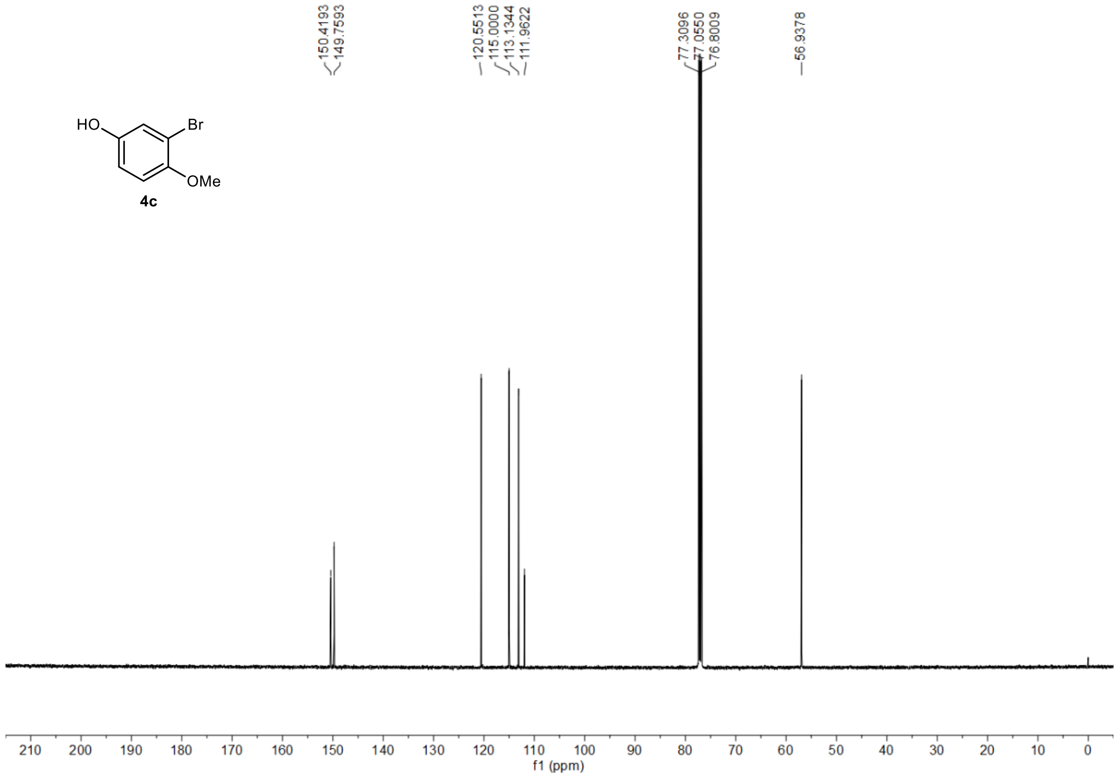

^{1}H NMR (500 MHz, $CDCl_3$)

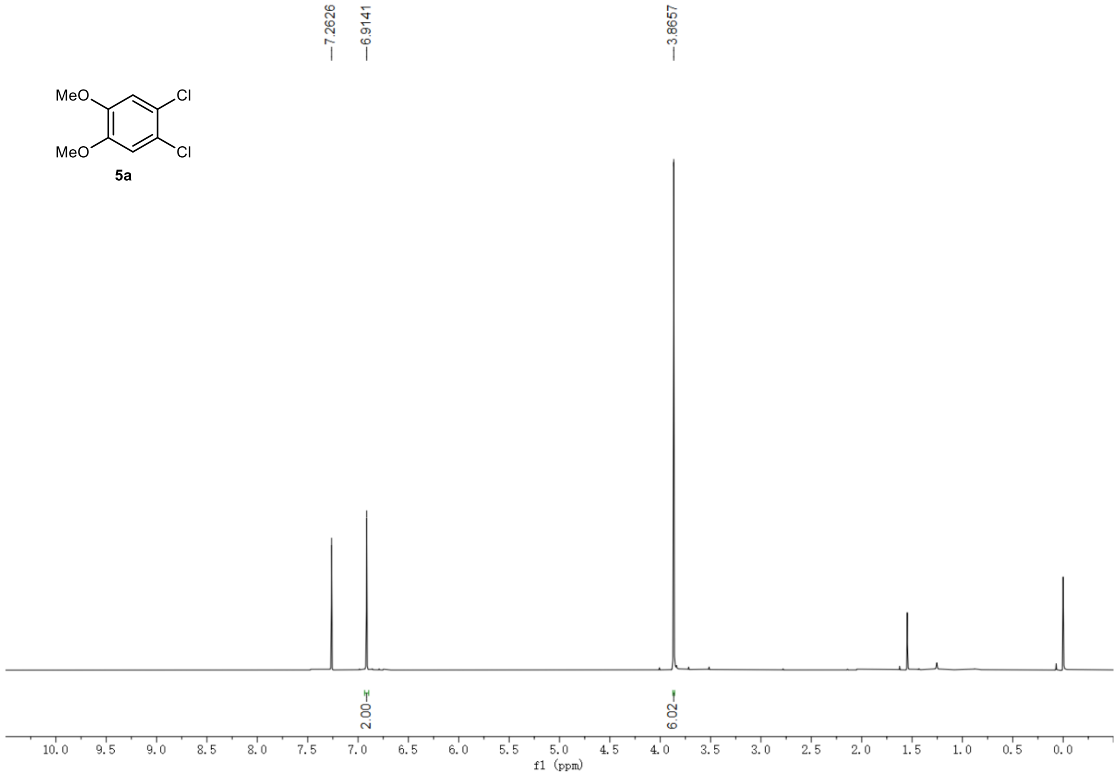

^{13}C NMR (126 MHz, $CDCl_3$)

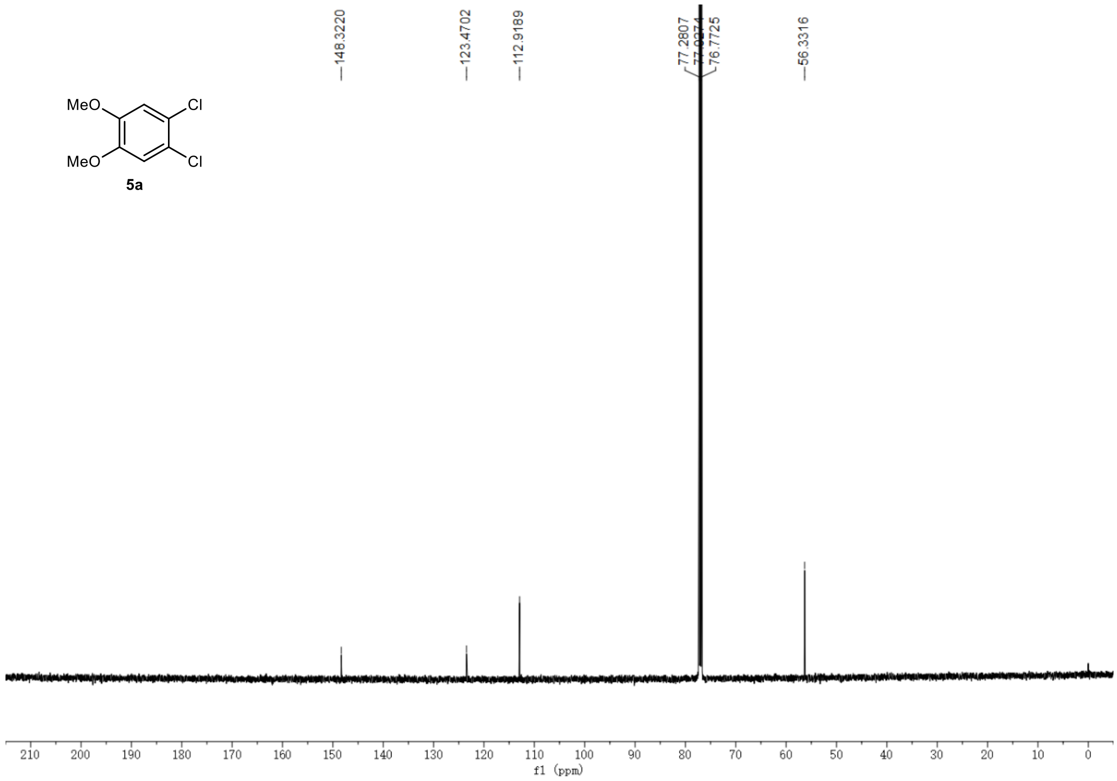

^{1}H NMR (500 MHz, $CDCl_3$)

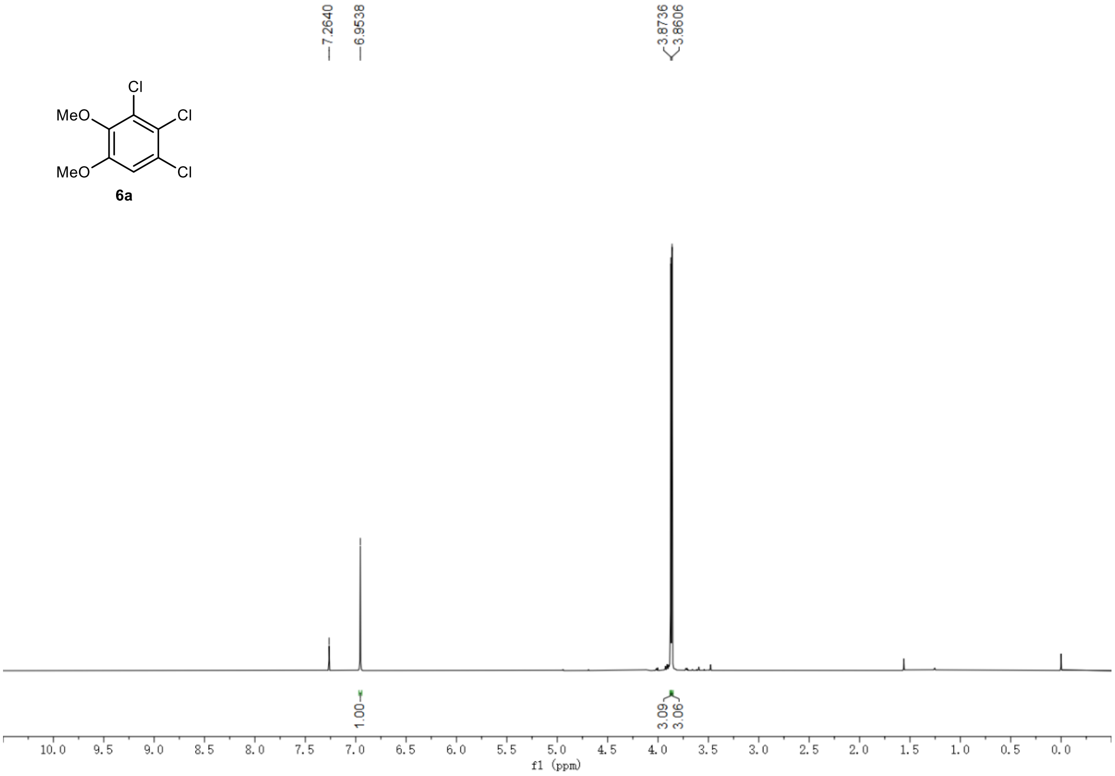

^{13}C NMR (126 MHz, $CDCl_3$)

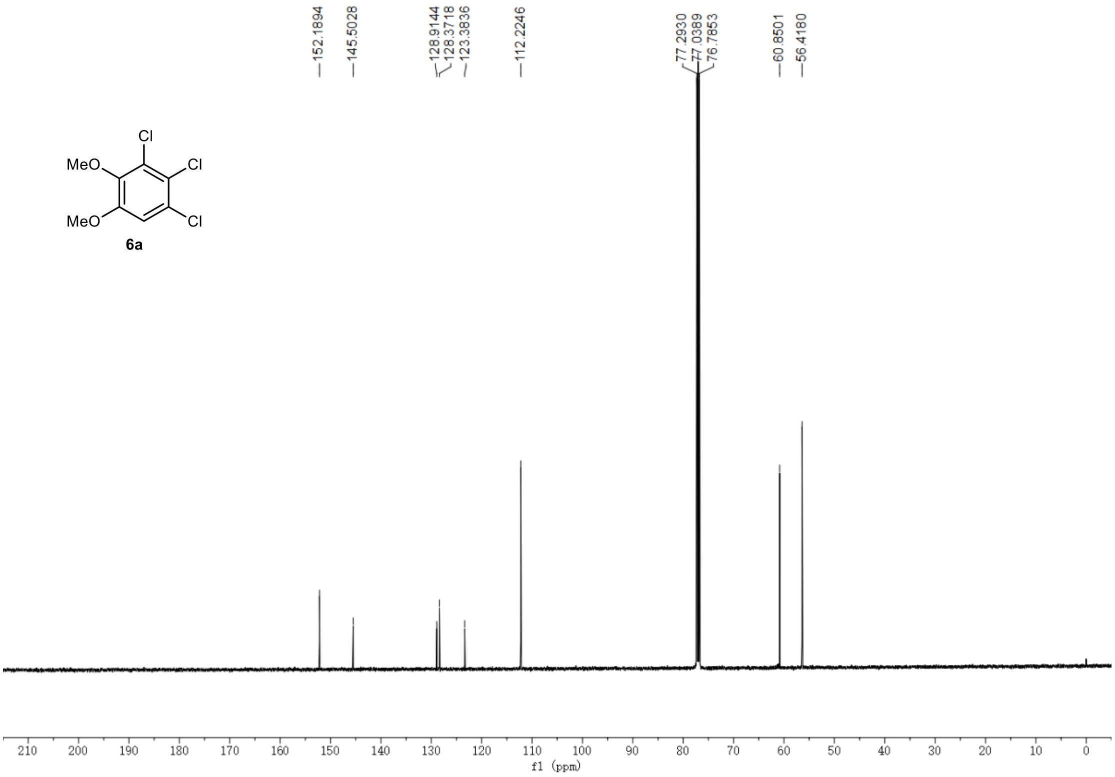

^{1}H NMR (500 MHz, $CDCl_3$)

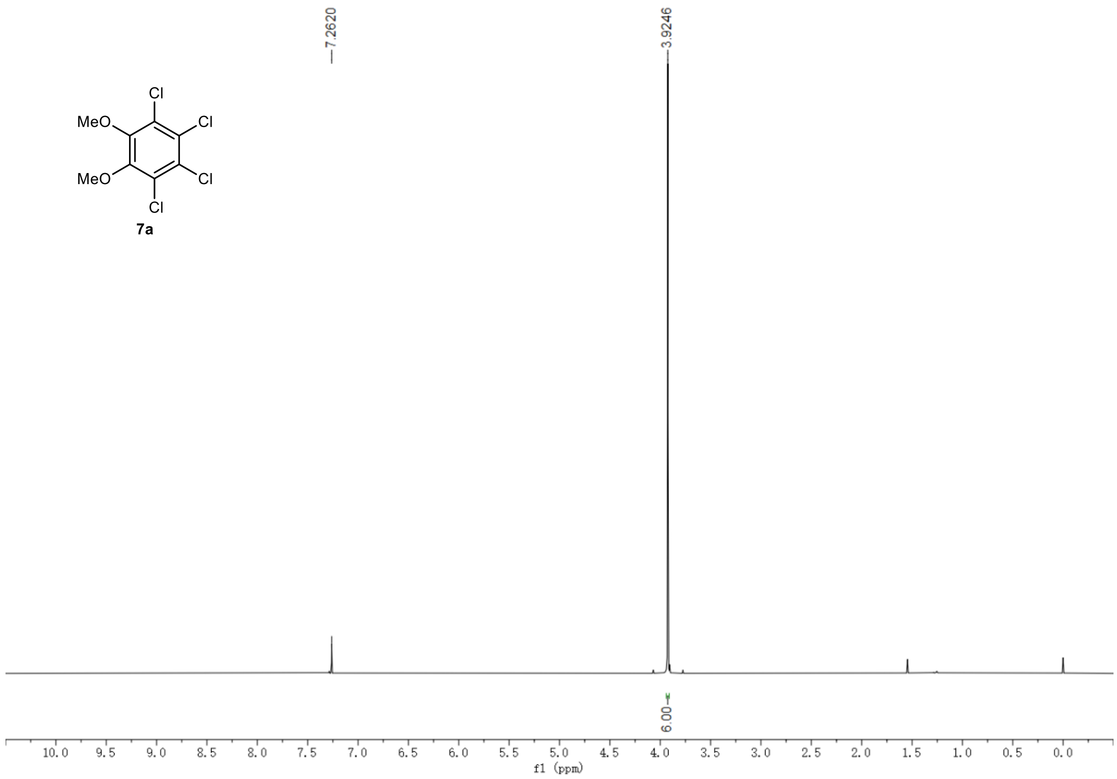

^{13}C NMR (126 MHz, $CDCl_3$)

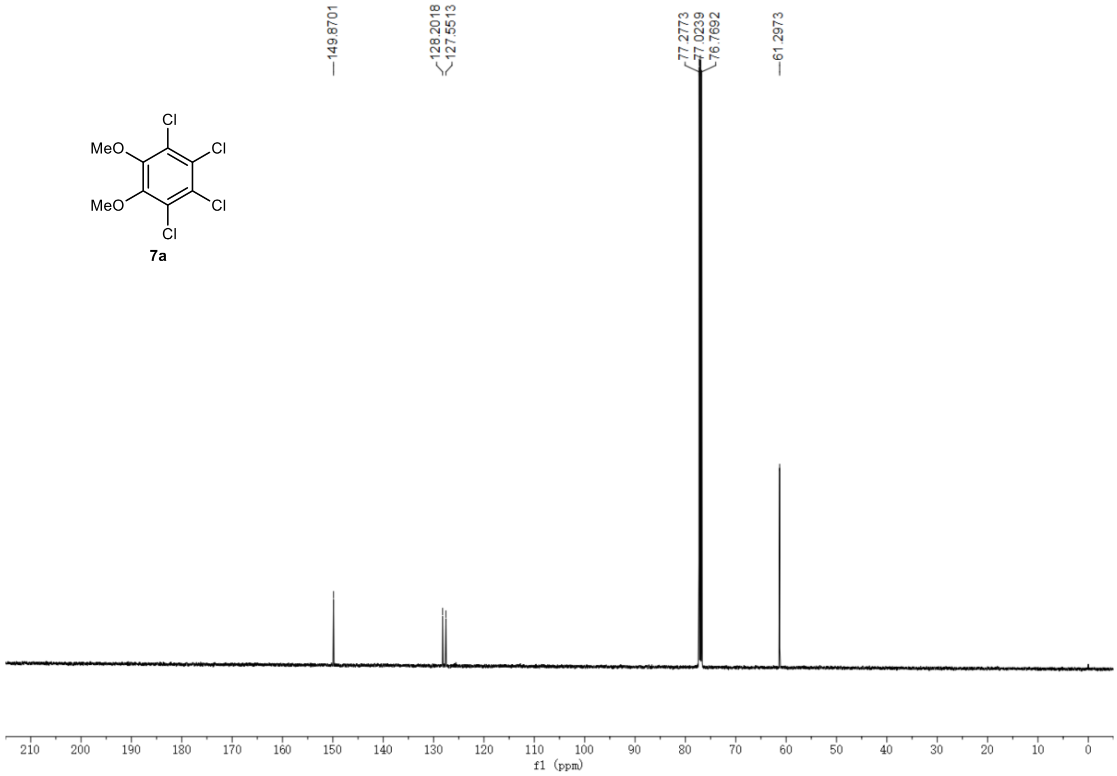

^{1}H NMR (500 MHz, $CDCl_3$)

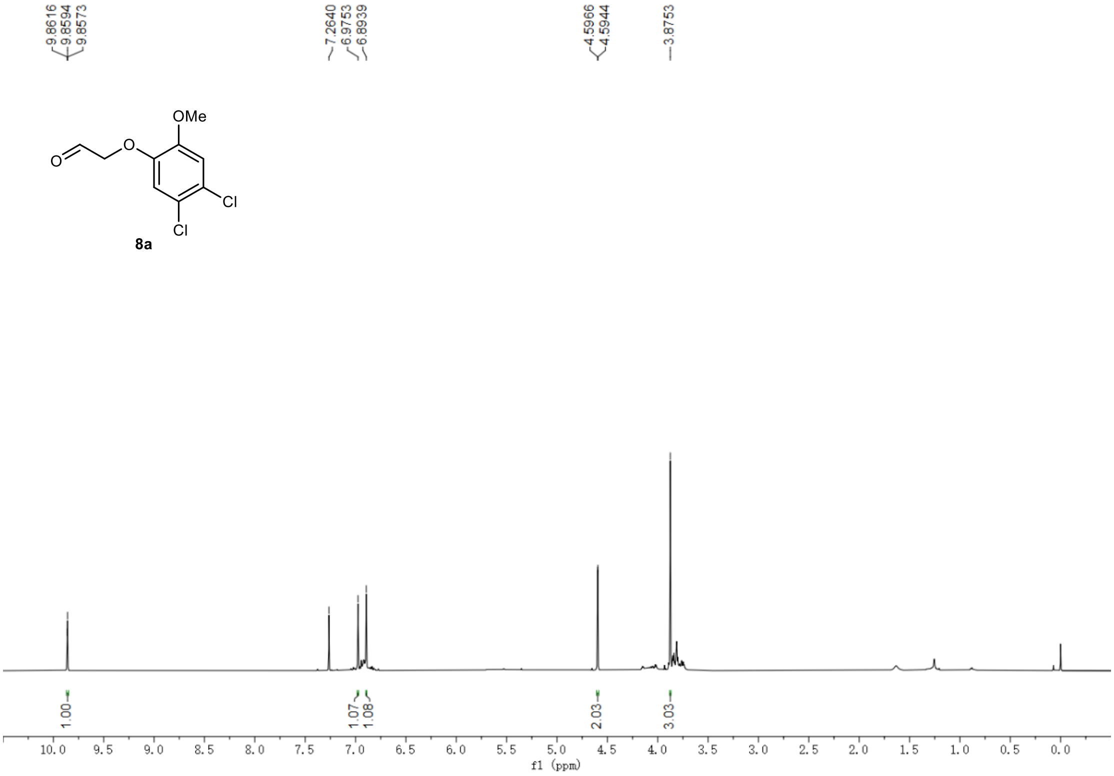

^{13}C NMR (126 MHz, $CDCl_3$)

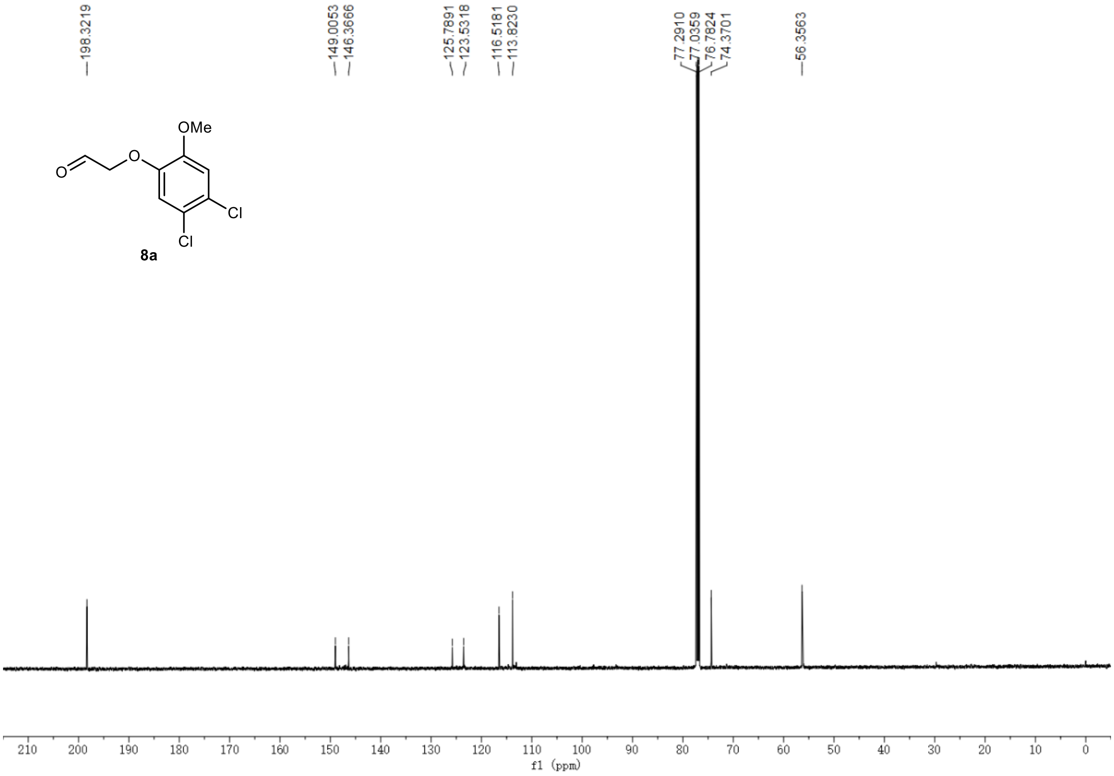

^{1}H NMR (500 MHz, $CDCl_3$)

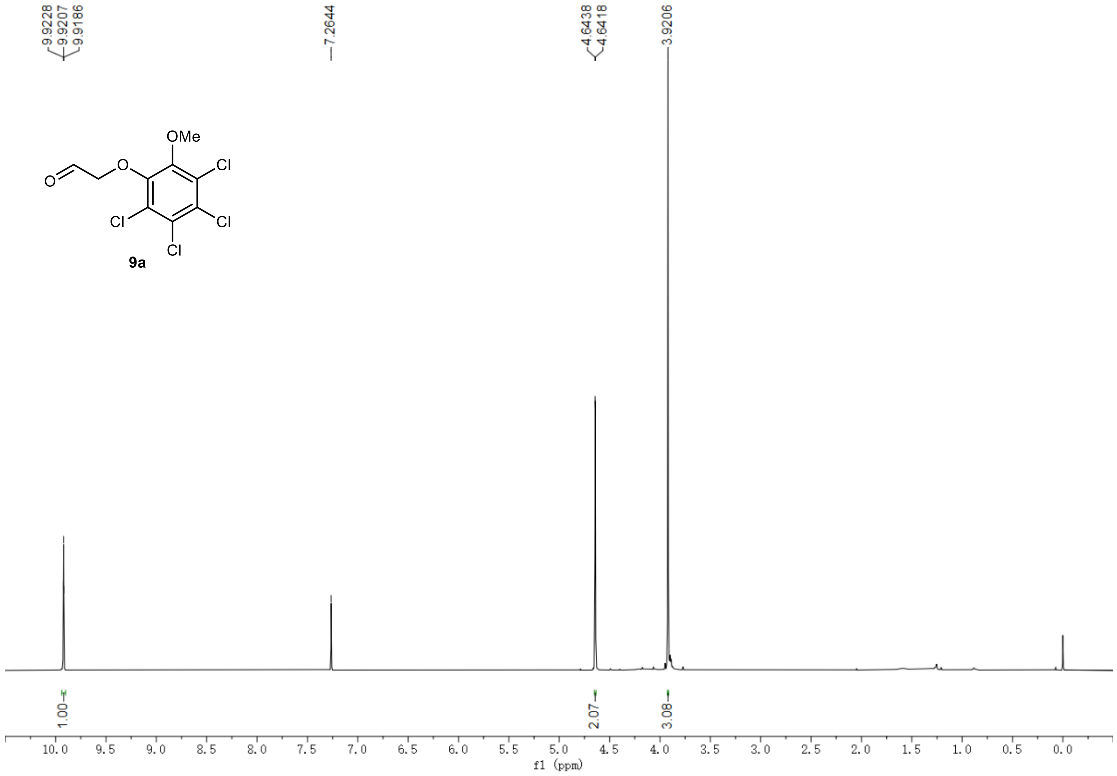

^{13}C NMR (126 MHz, $CDCl_3$)

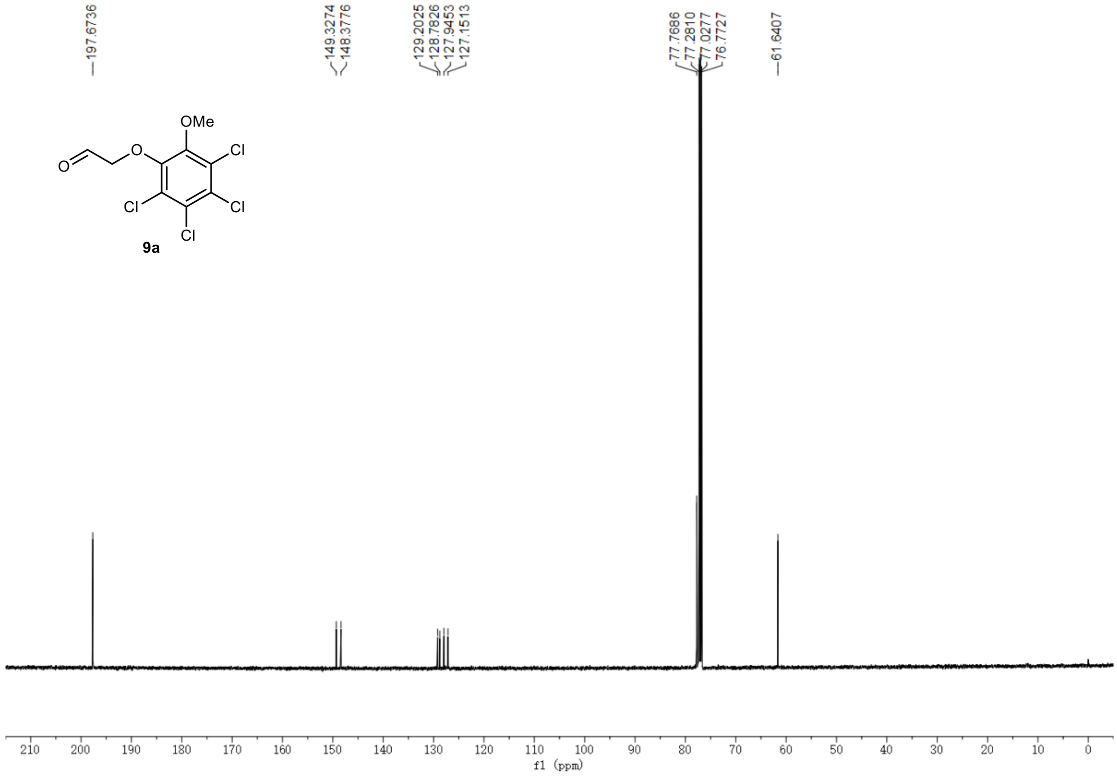

^{1}**H NMR** (500 MHz, $CDCl_3$)

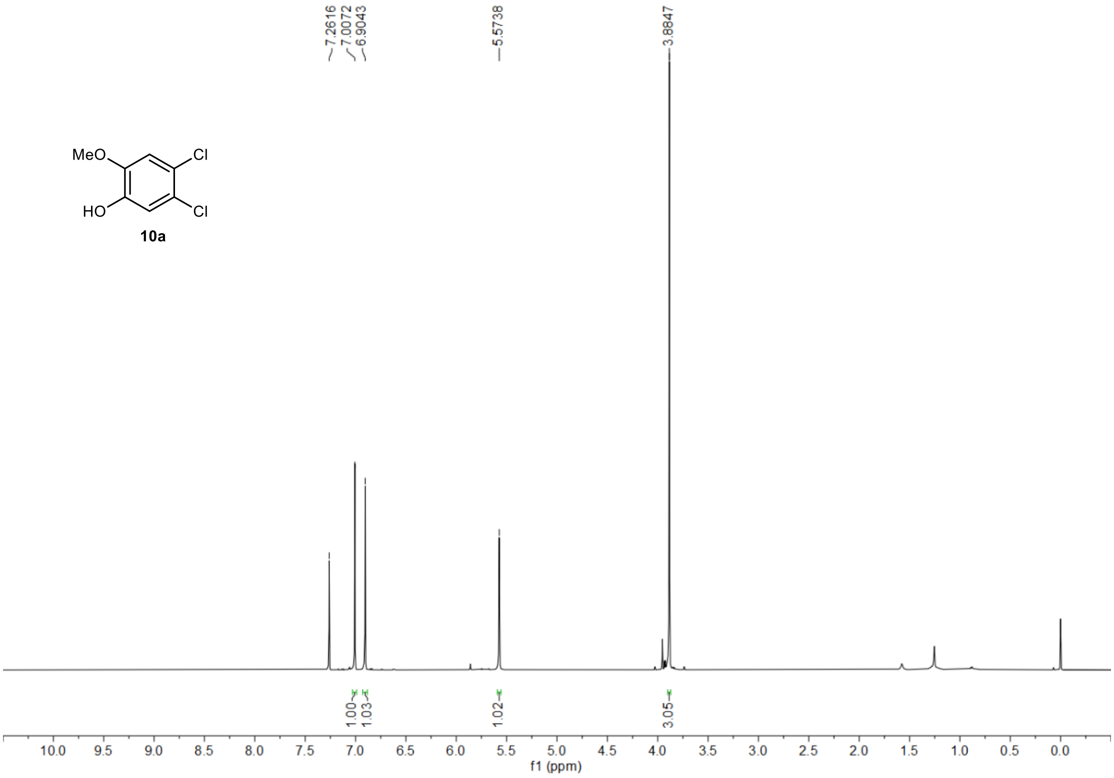

^{13}**C NMR** (126 MHz, $CDCl_3$)

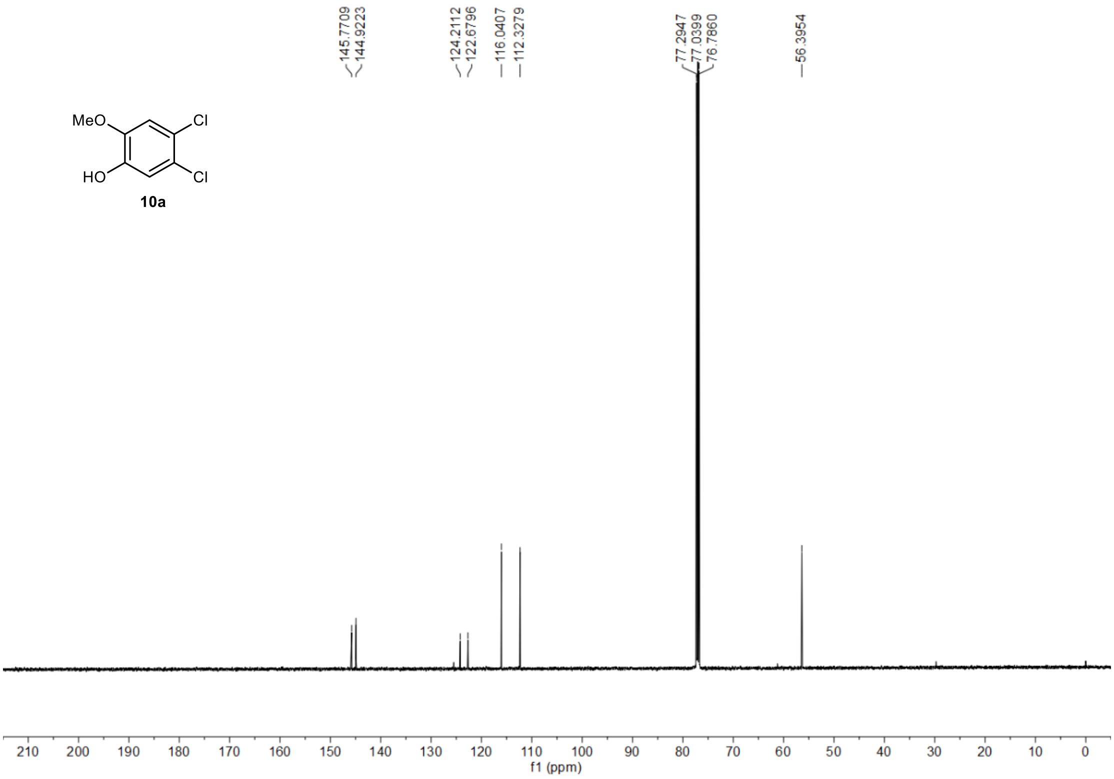

^{1}H NMR (500 MHz, $CDCl_3$)

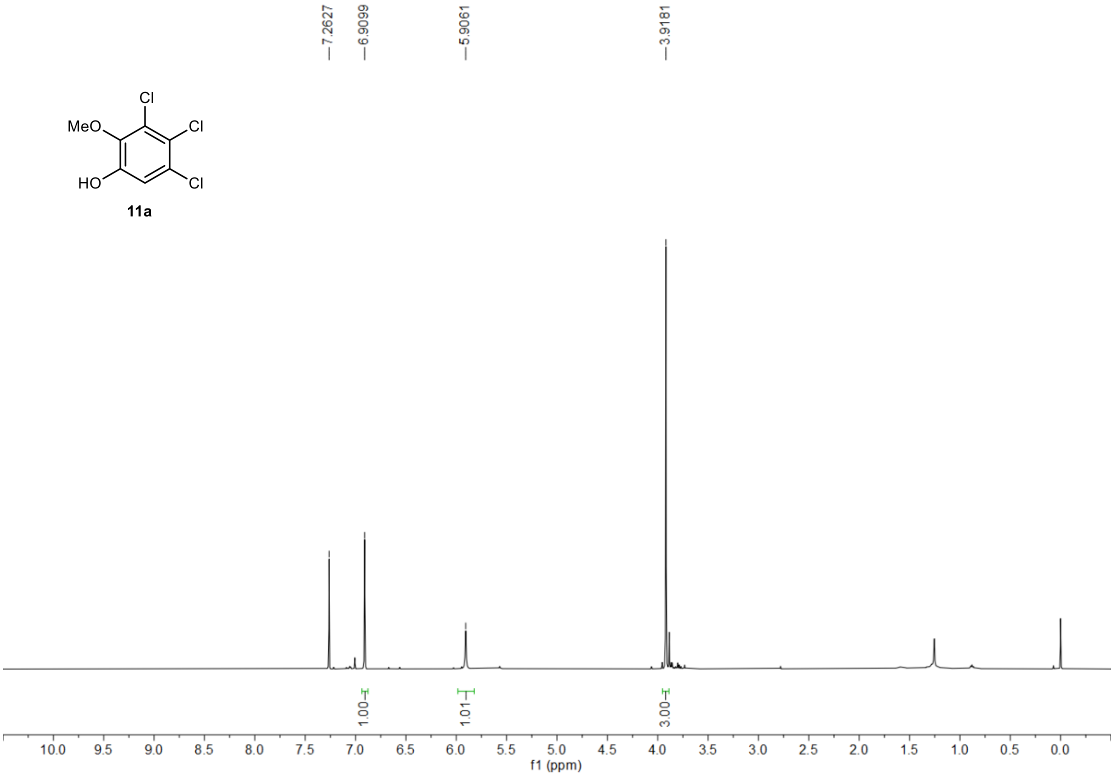

^{13}C NMR (126 MHz, $CDCl_3$)

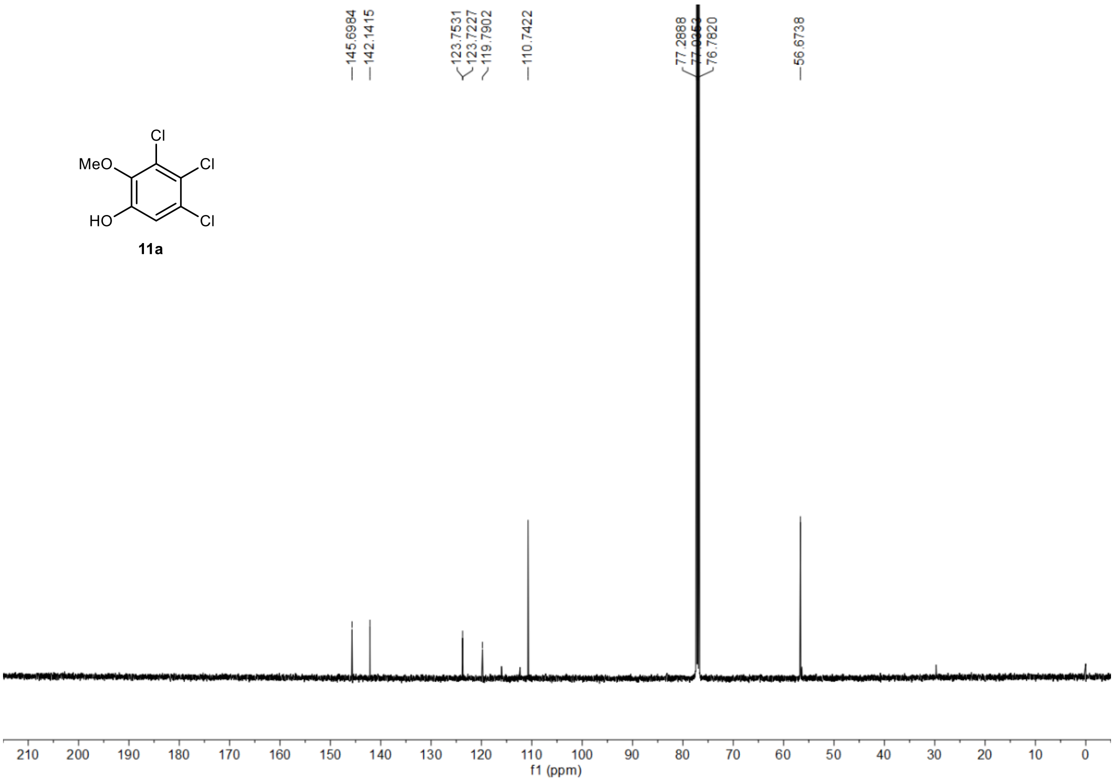

^{1}H NMR (500 MHz, $CDCl_3$)

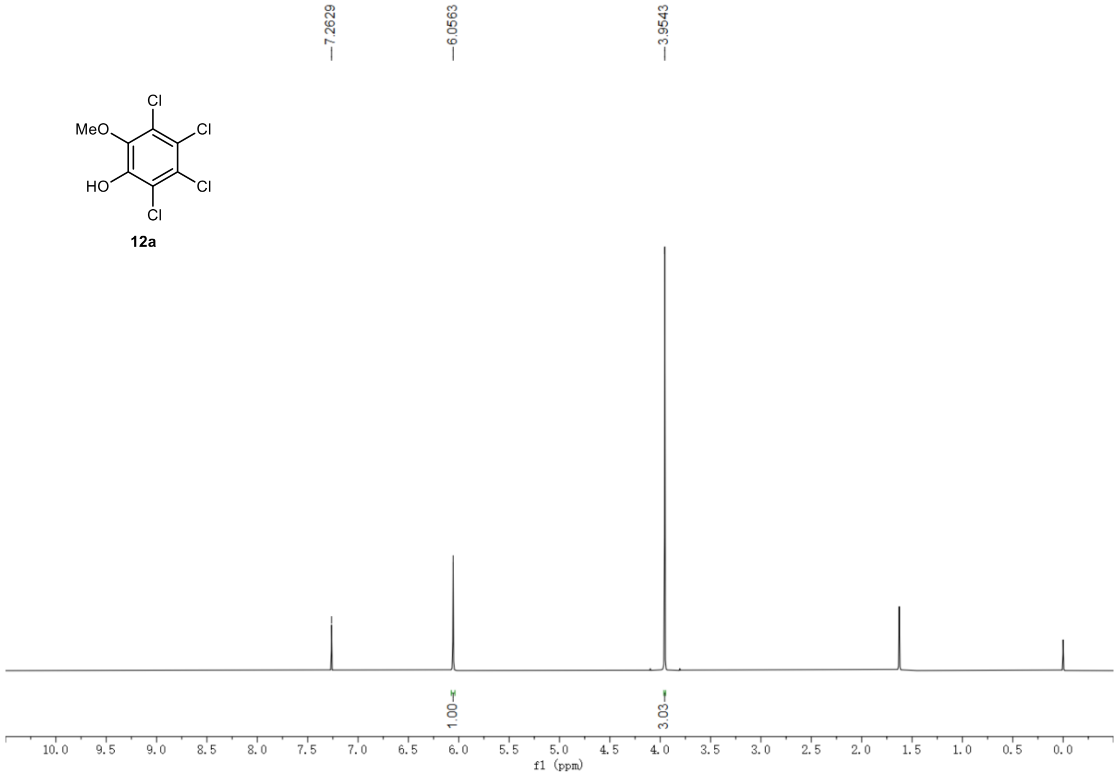

^{13}C NMR (126 MHz, $CDCl_3$)

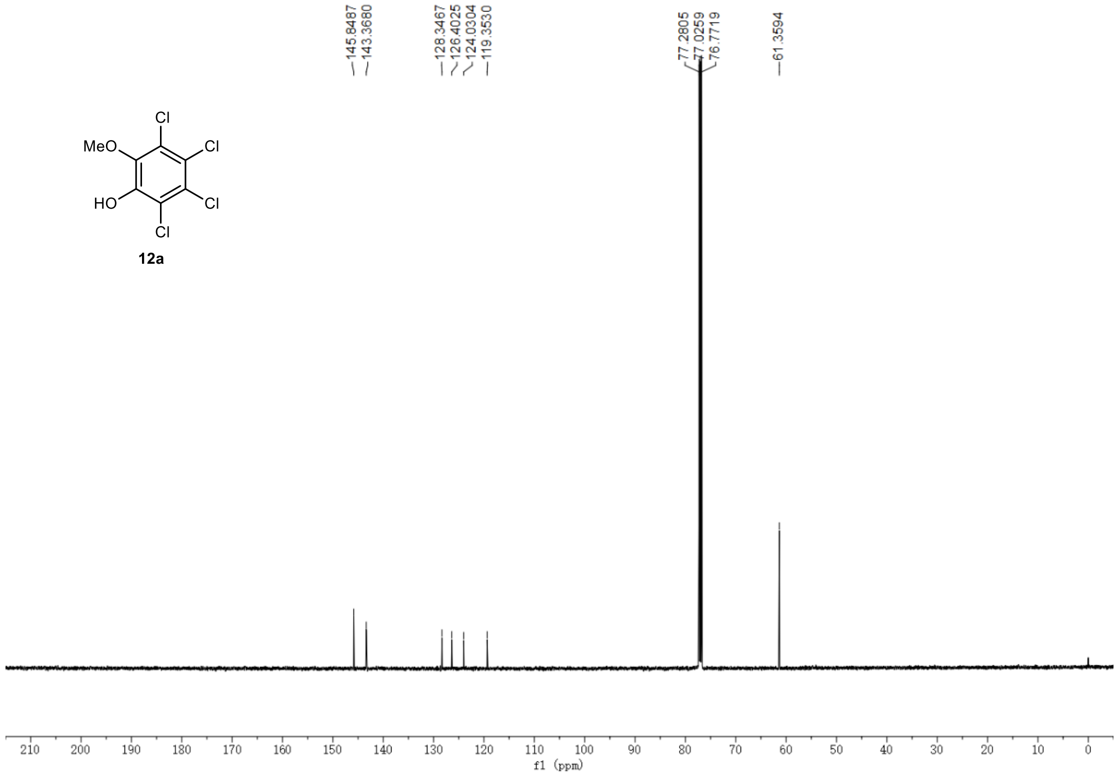

^{1}H NMR (500 MHz, $CDCl_3$)

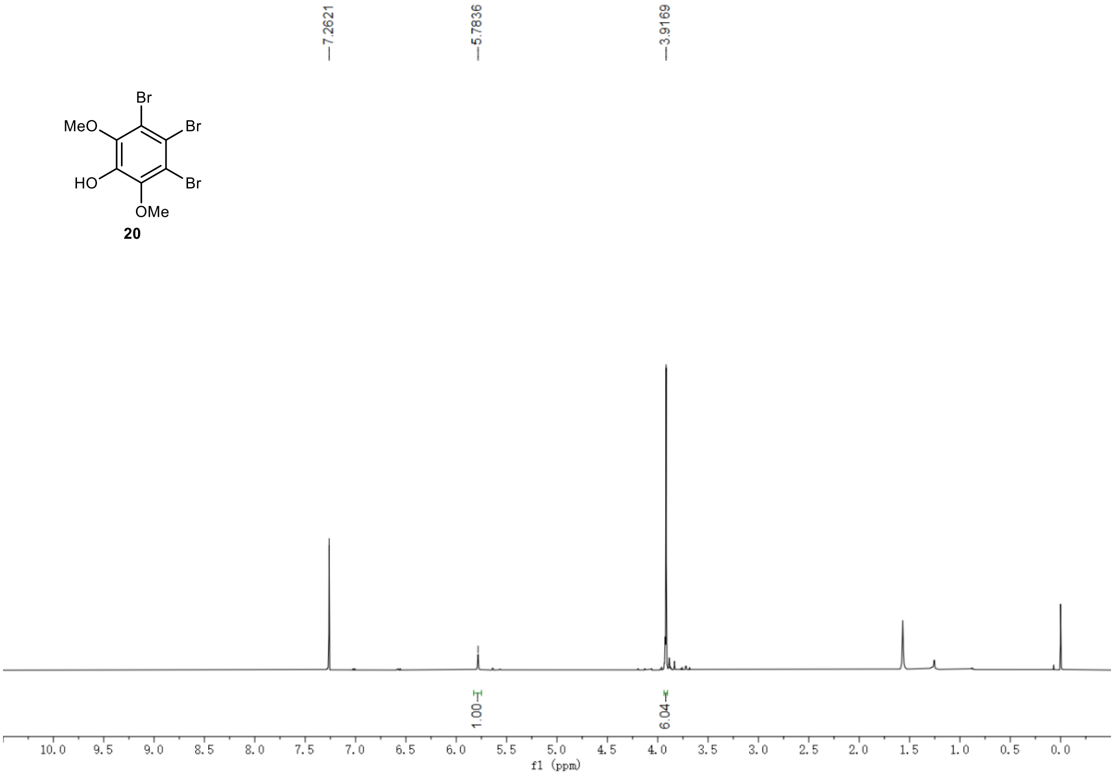

^{13}C NMR (126 MHz, $CDCl_3$)

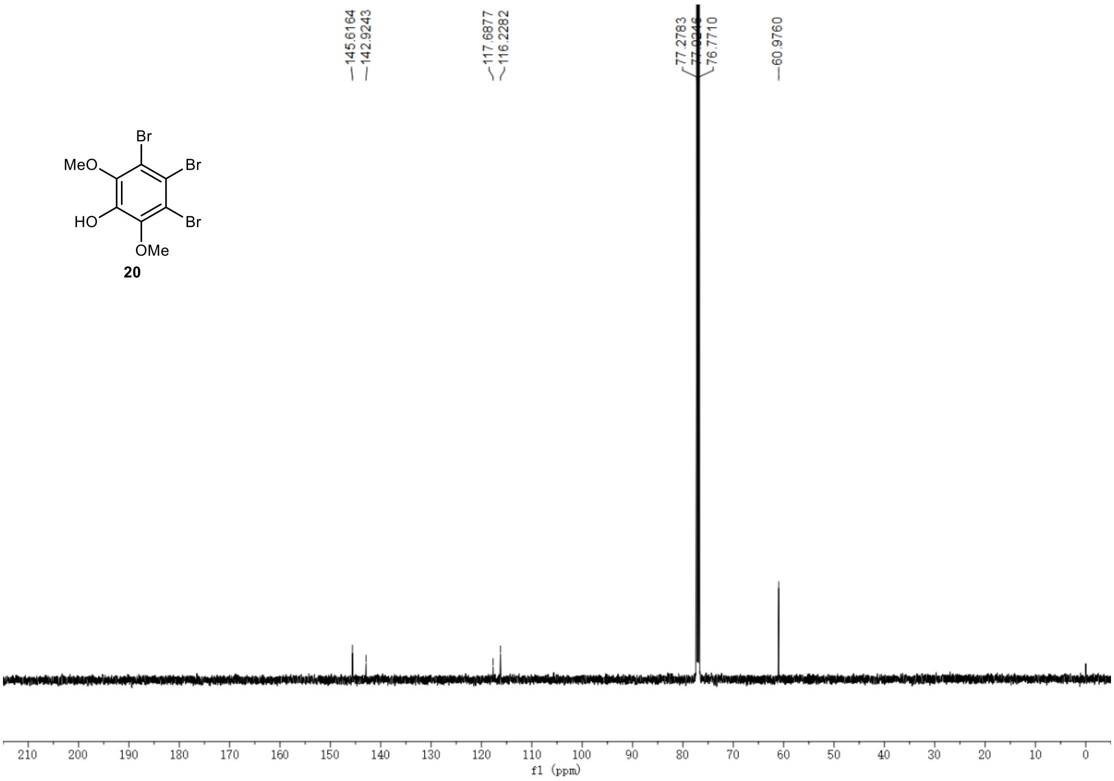

^{1}H NMR (500 MHz, $CDCl_3$)

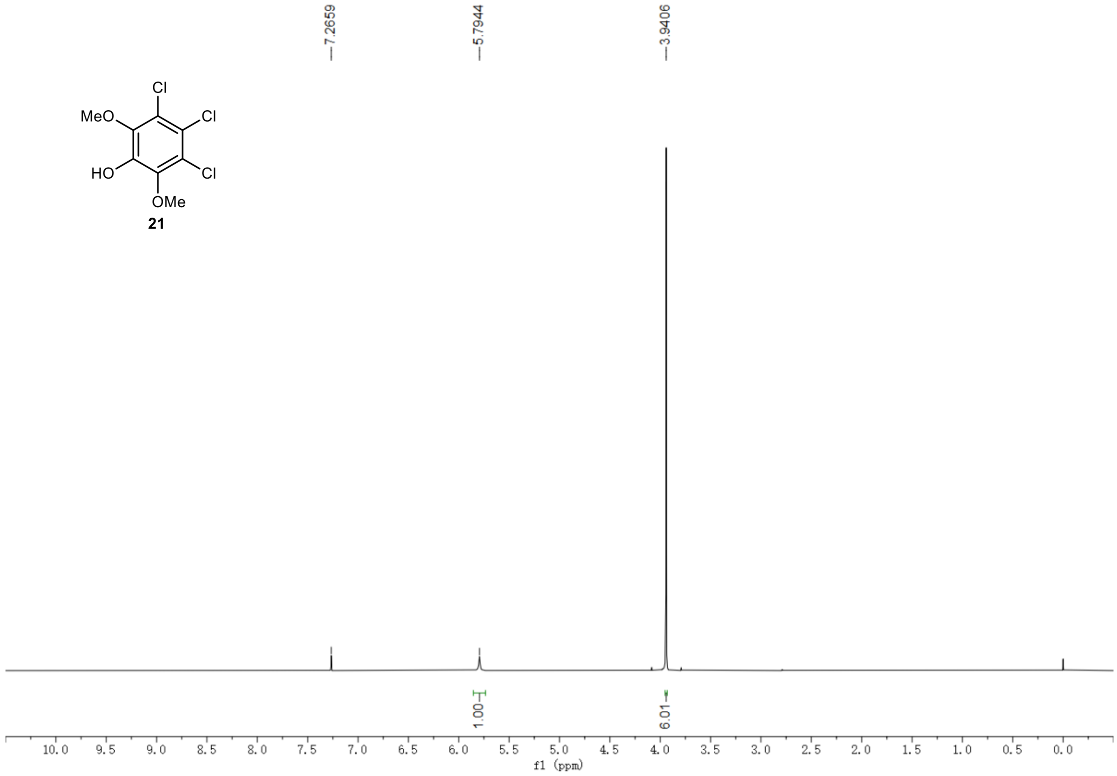

^{13}C NMR (126 MHz, $CDCl_3$)

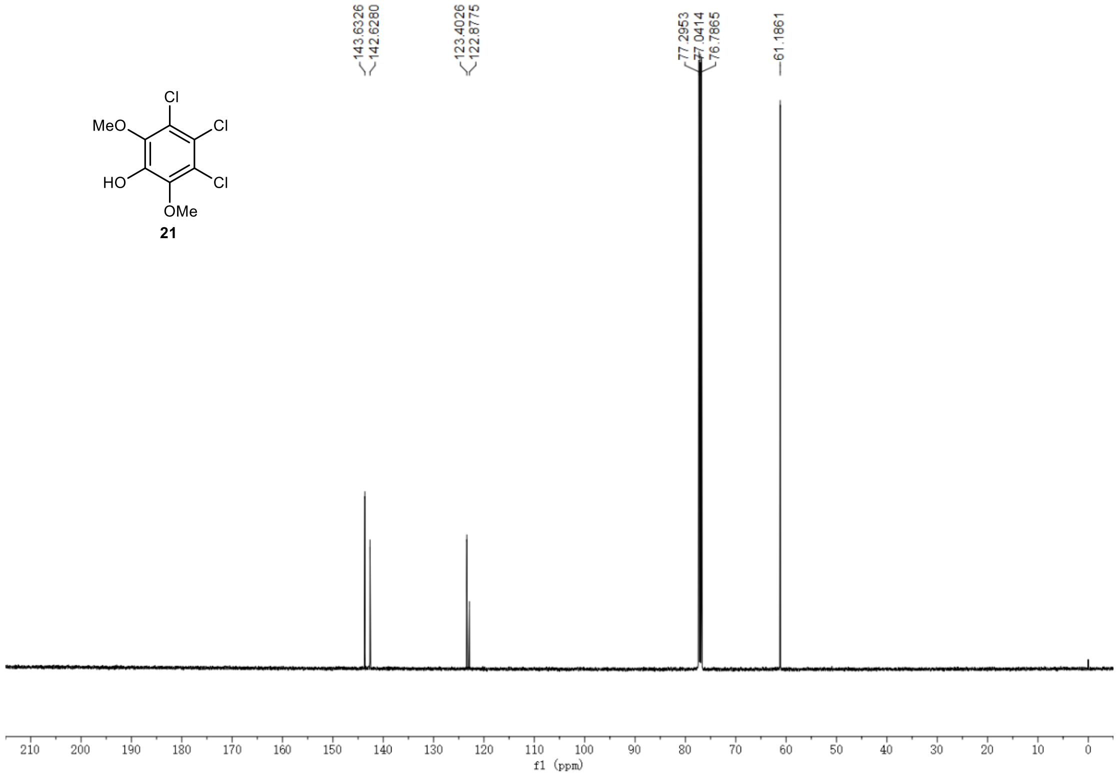

^{1}H NMR (500 MHz, $CDCl_3$)

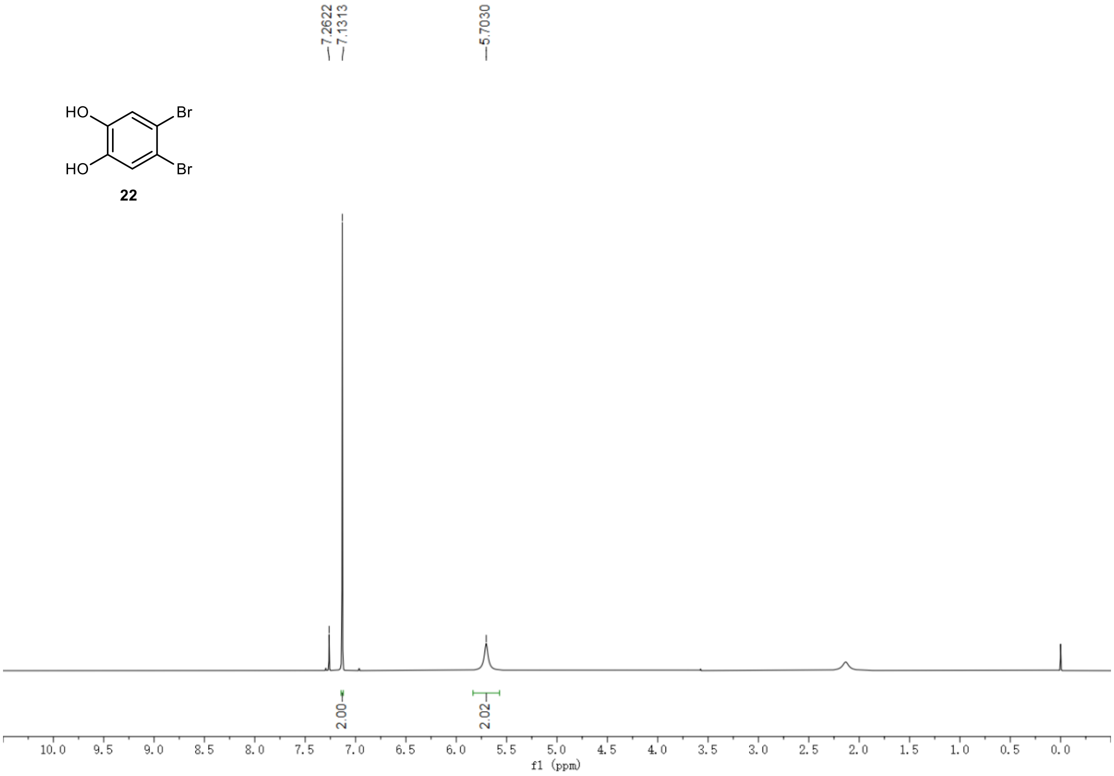

^{13}C NMR (126 MHz, $CDCl_3$)

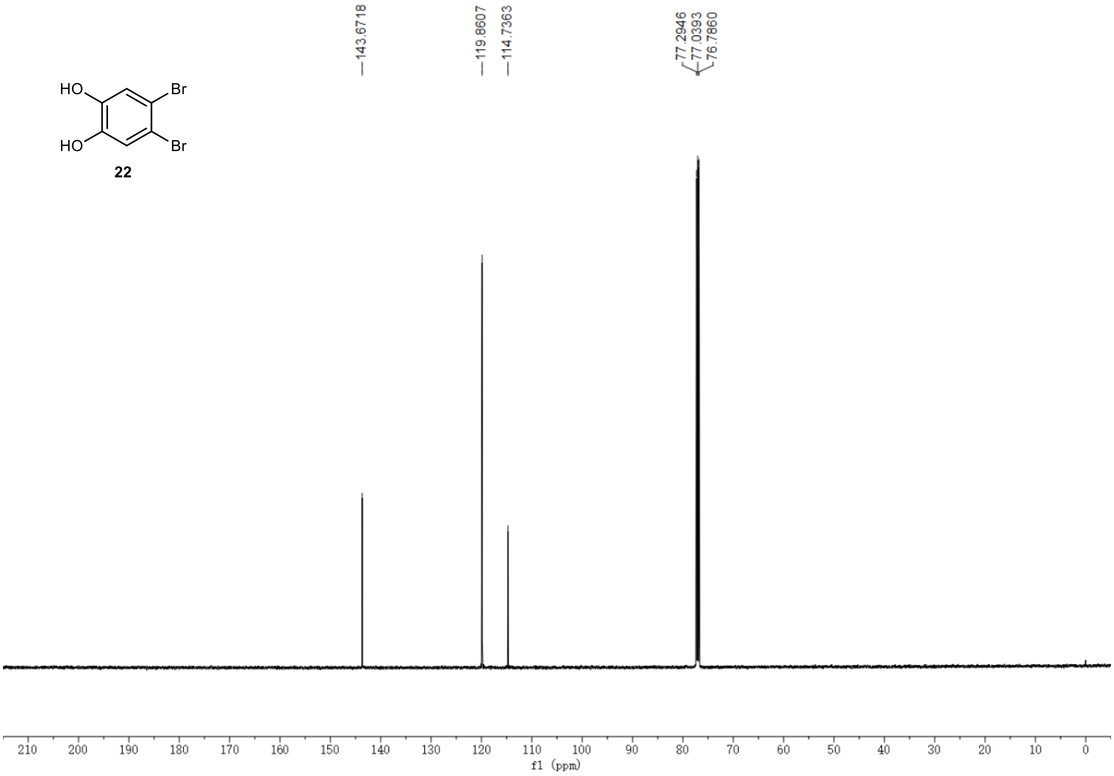

^{1}H NMR (500 MHz, $CDCl_3$)

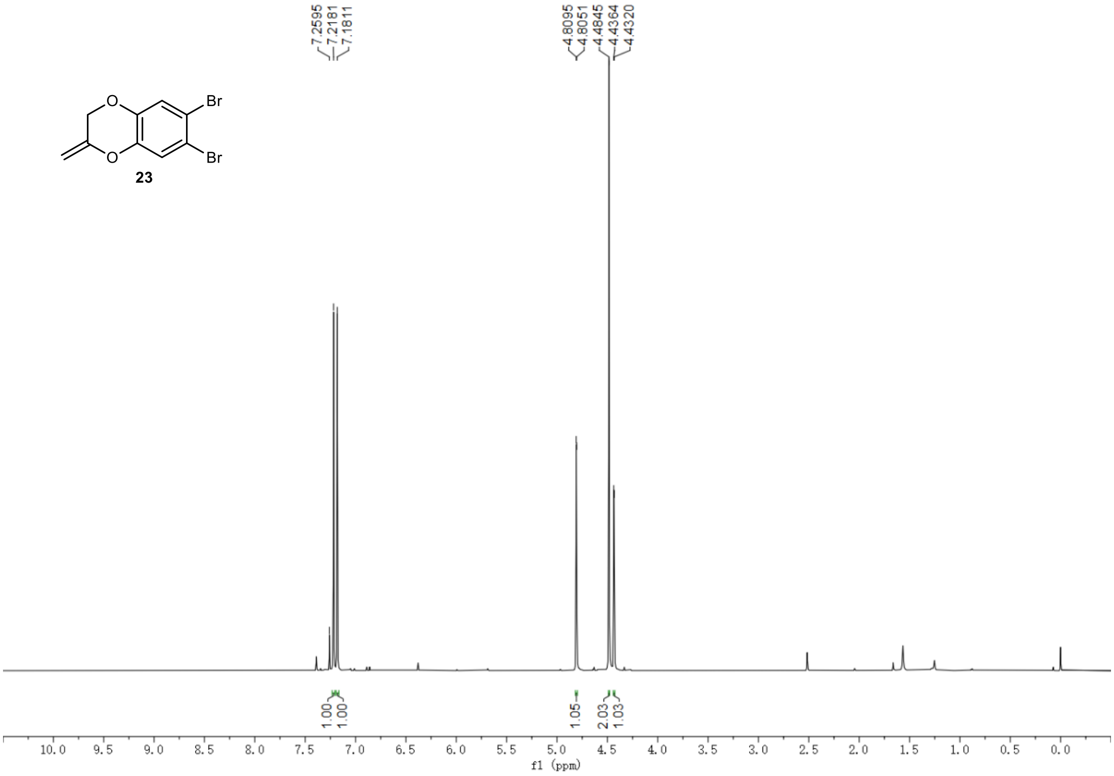

^{13}C NMR (126 MHz, $CDCl_3$)

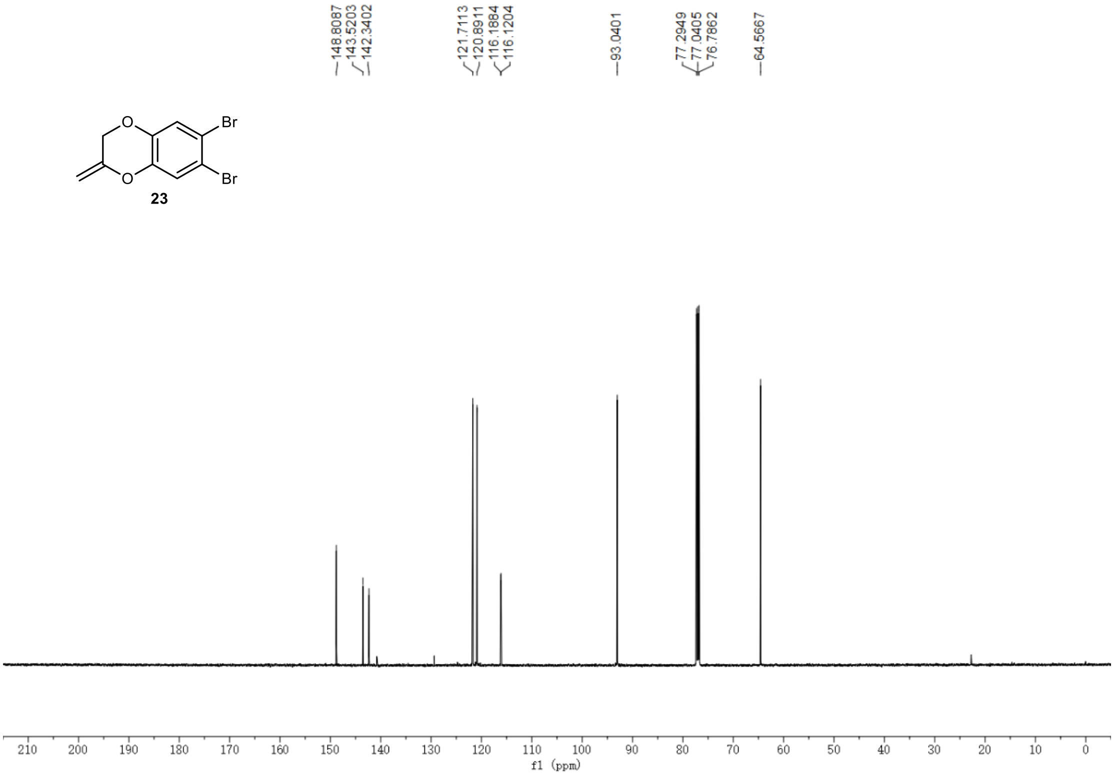

^{1}H NMR (500 MHz, $CDCl_3$)

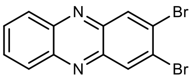

24

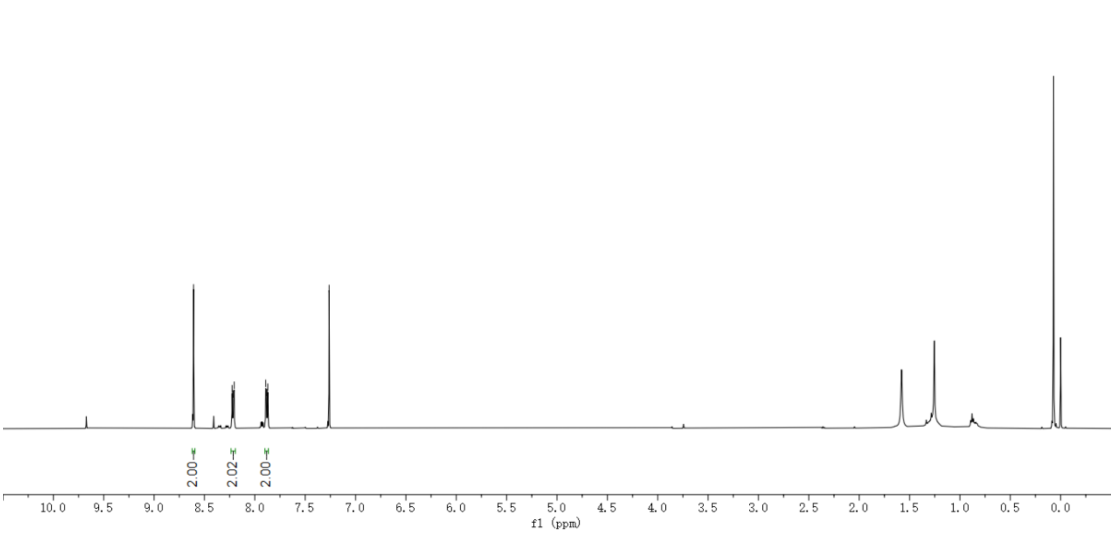

^{13}C NMR (126 MHz, $CDCl_3$)

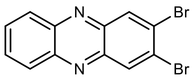

24

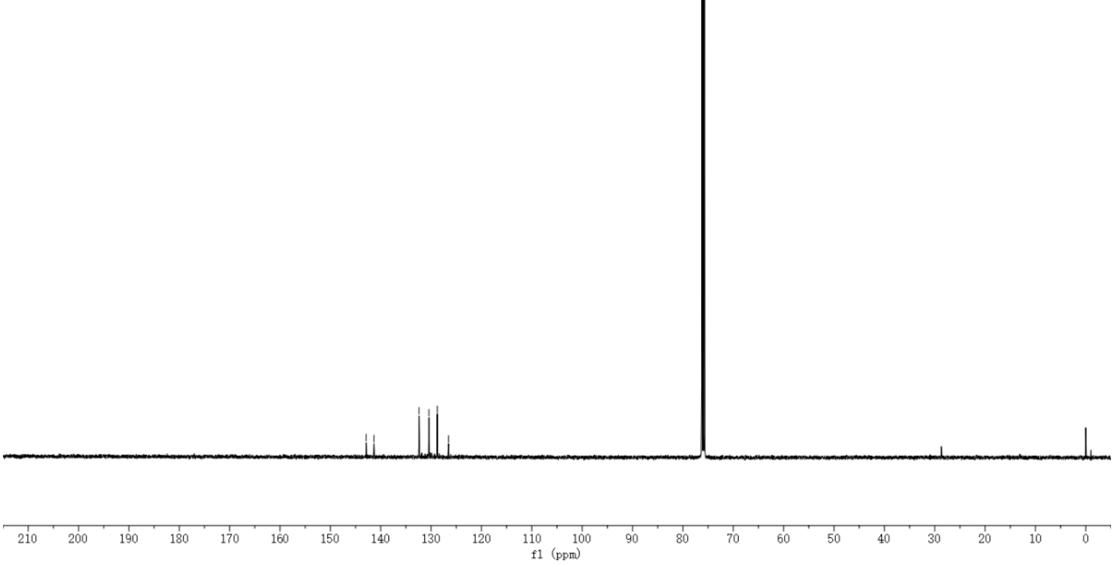

^{1}H NMR (500 MHz, $CDCl_3$)

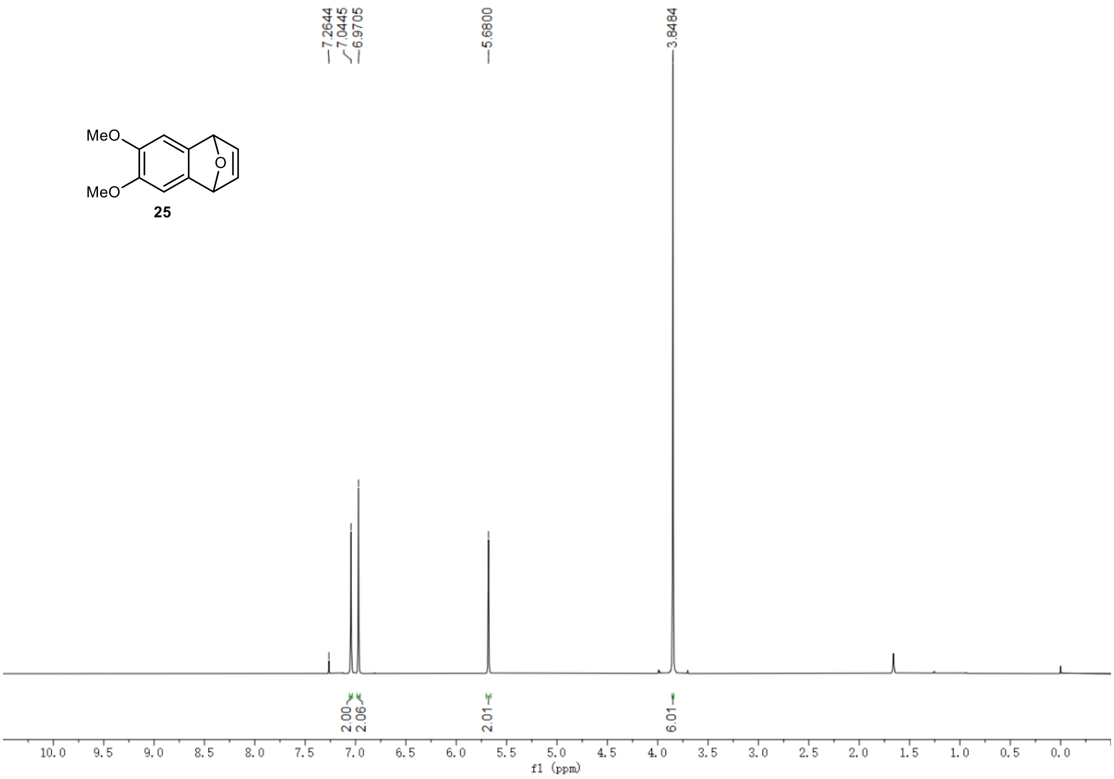

^{13}C NMR (126 MHz, $CDCl_3$)

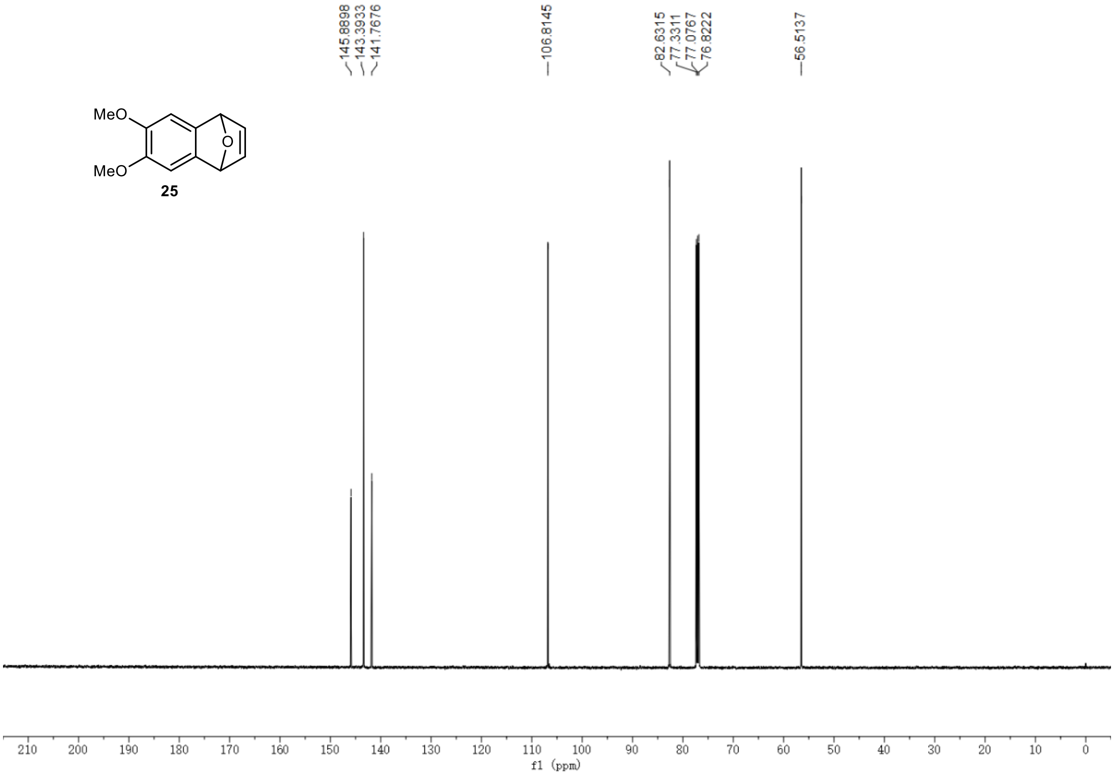

^{1}H NMR (500 MHz, $CDCl_3$)

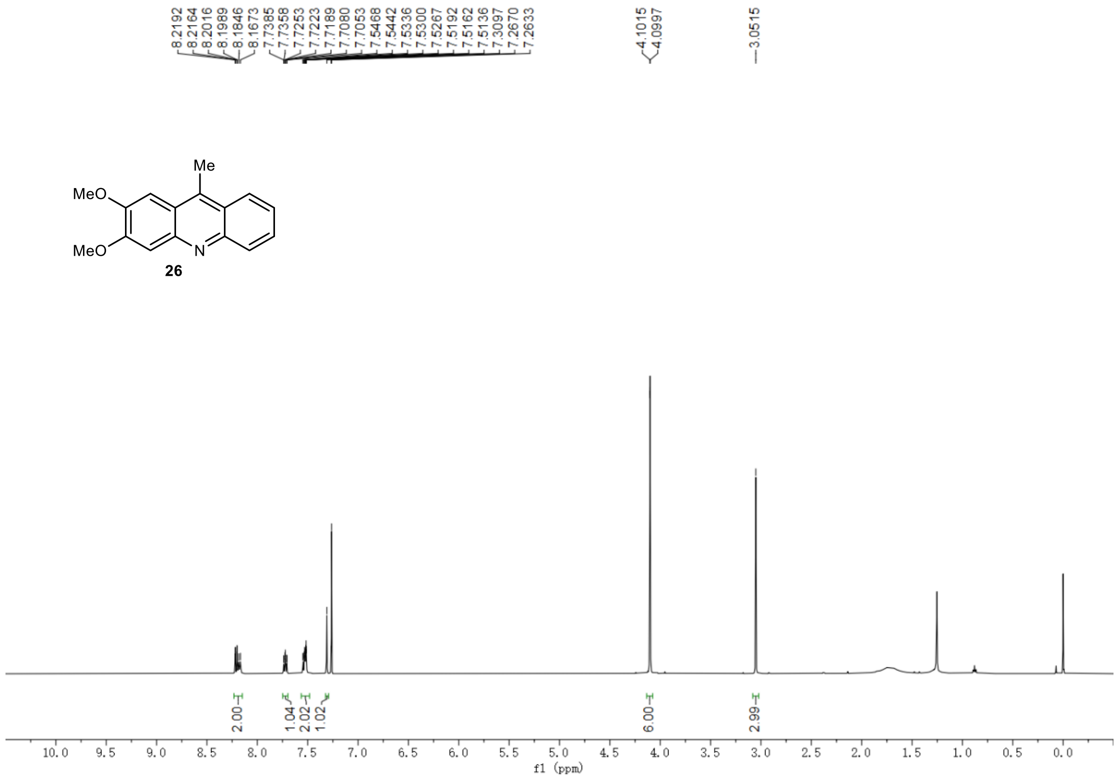

^{13}C NMR (126 MHz, $CDCl_3$)

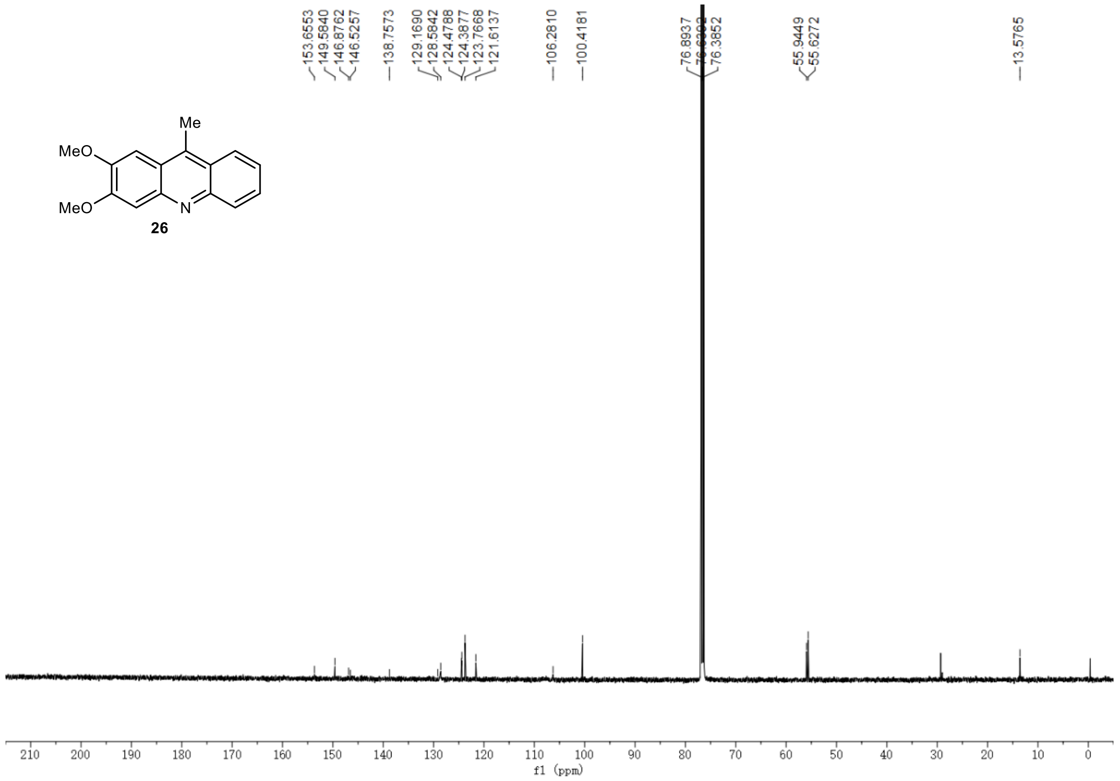

^{1}H NMR (500 MHz, $CDCl_3$)

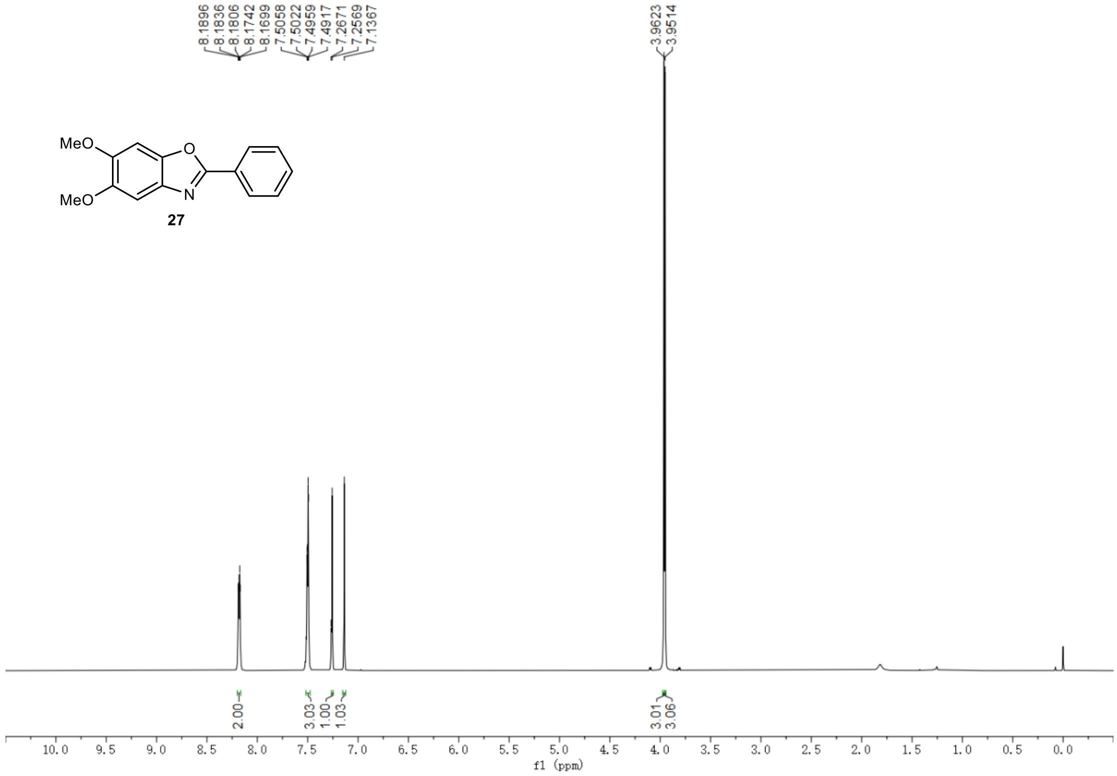

^{13}C NMR (126 MHz, $CDCl_3$)

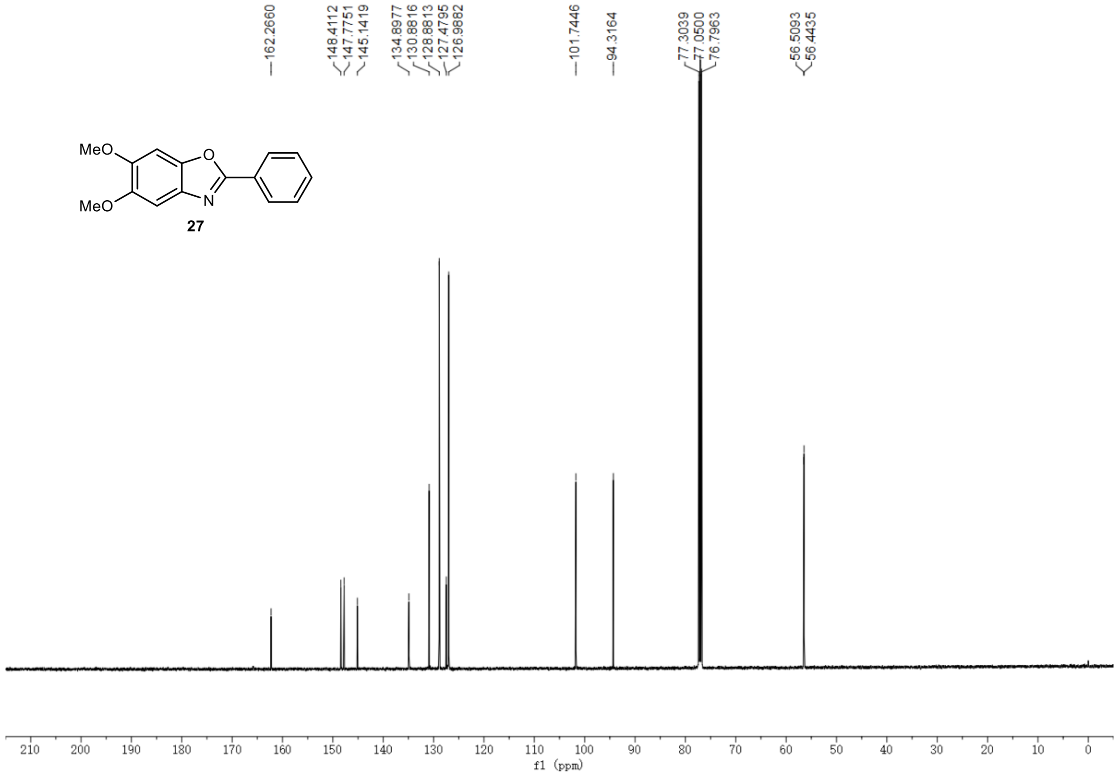

^{1}H NMR (500 MHz, $CDCl_3$)

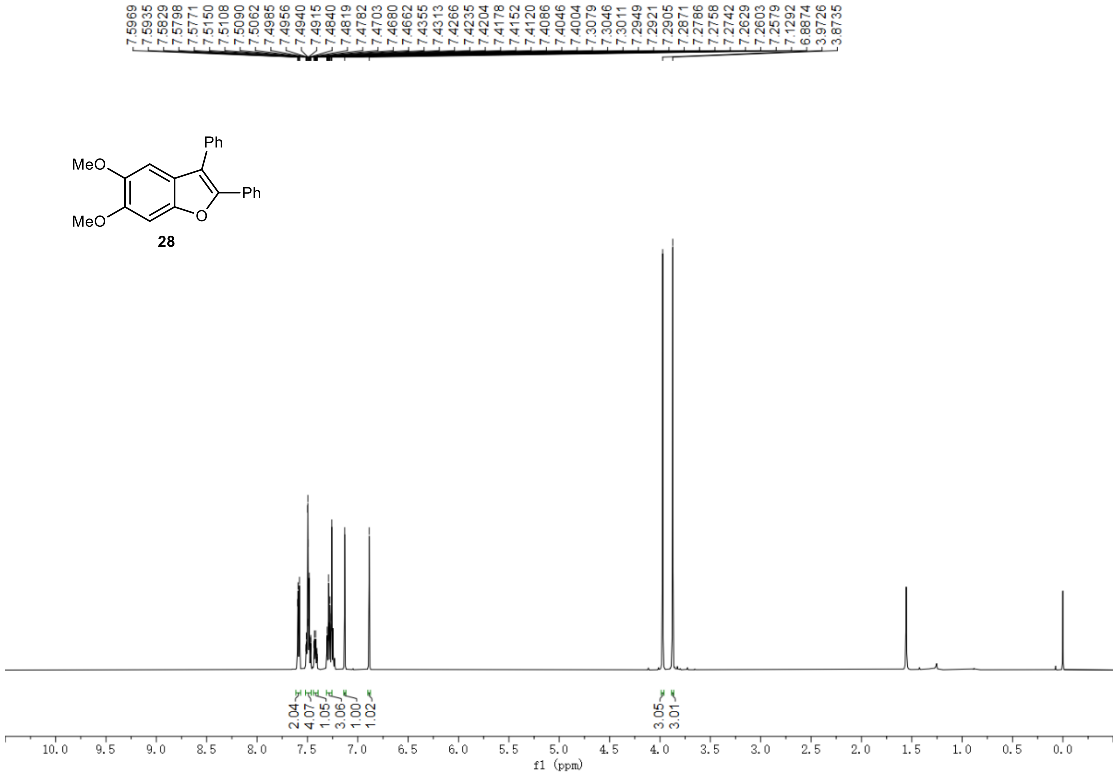

^{13}C NMR (126 MHz, $CDCl_3$)

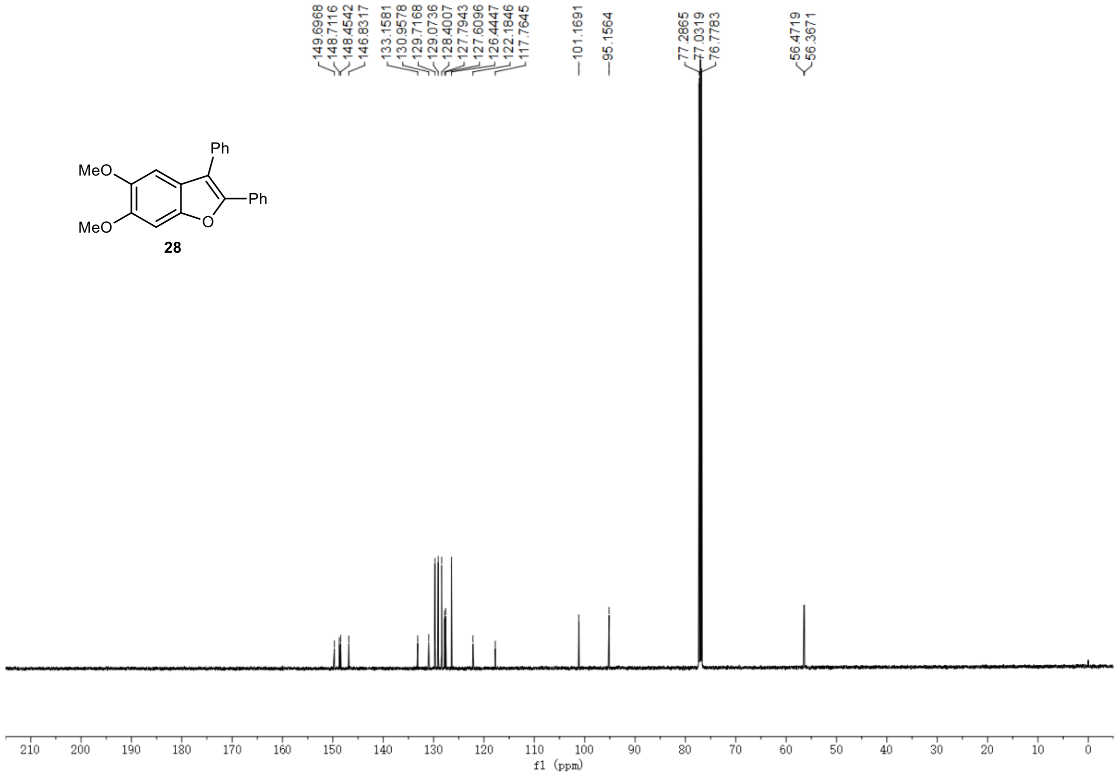

^{1}H NMR (500 MHz, $CDCl_3$)

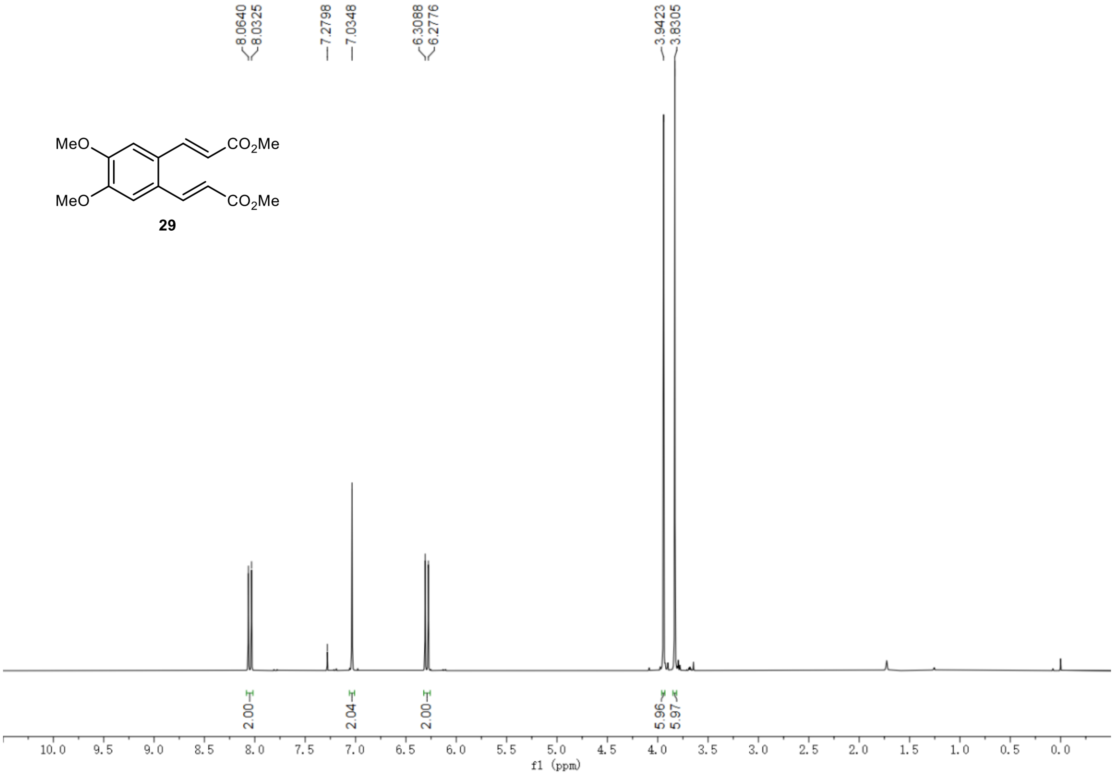

^{13}C NMR (126 MHz, $CDCl_3$)

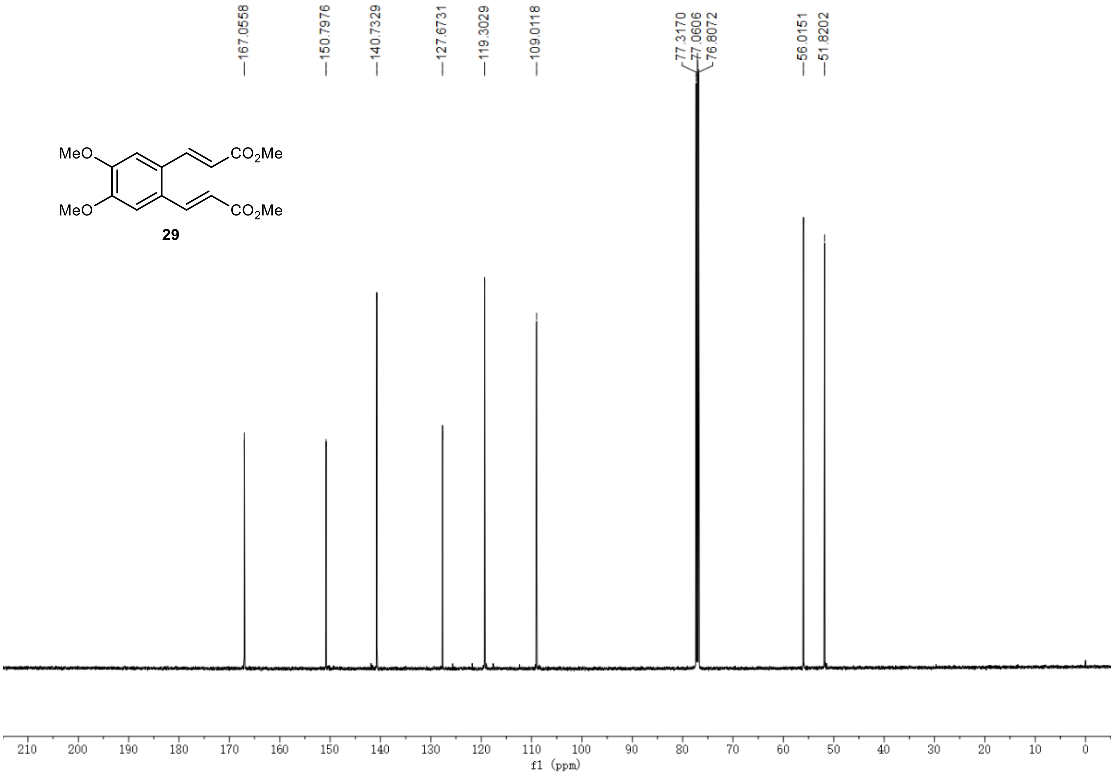

^{1}H NMR (500 MHz, $CDCl_3$)

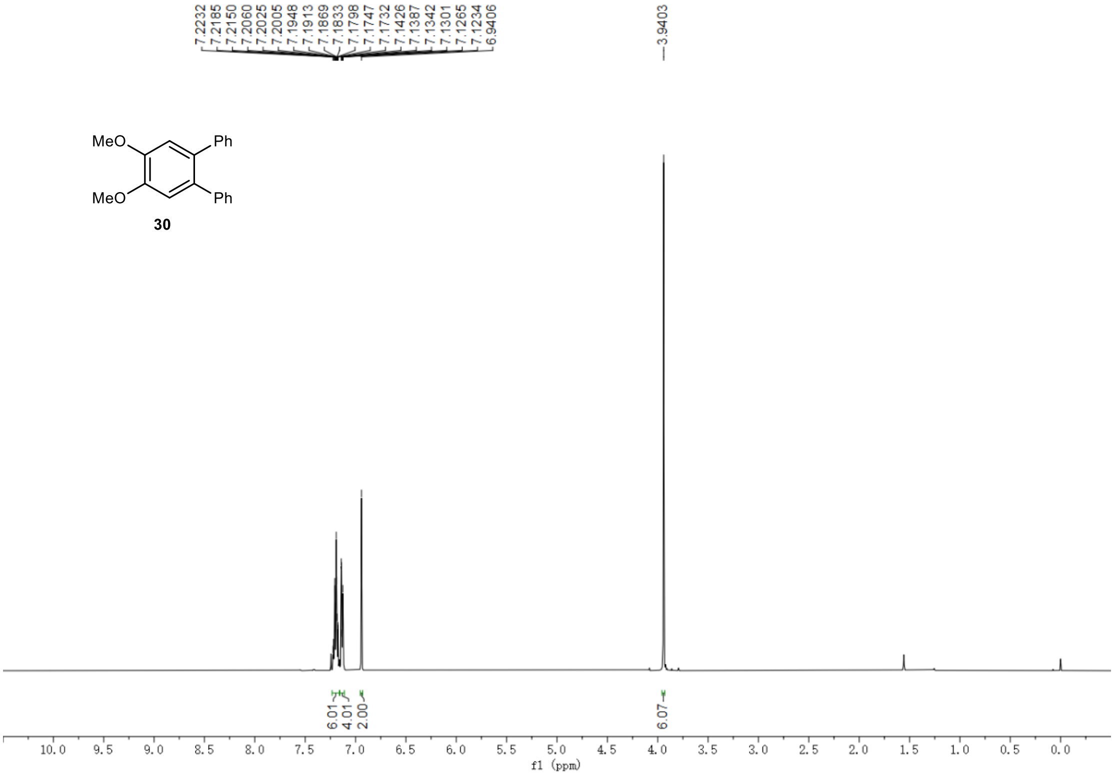

^{13}C NMR (126 MHz, $CDCl_3$)

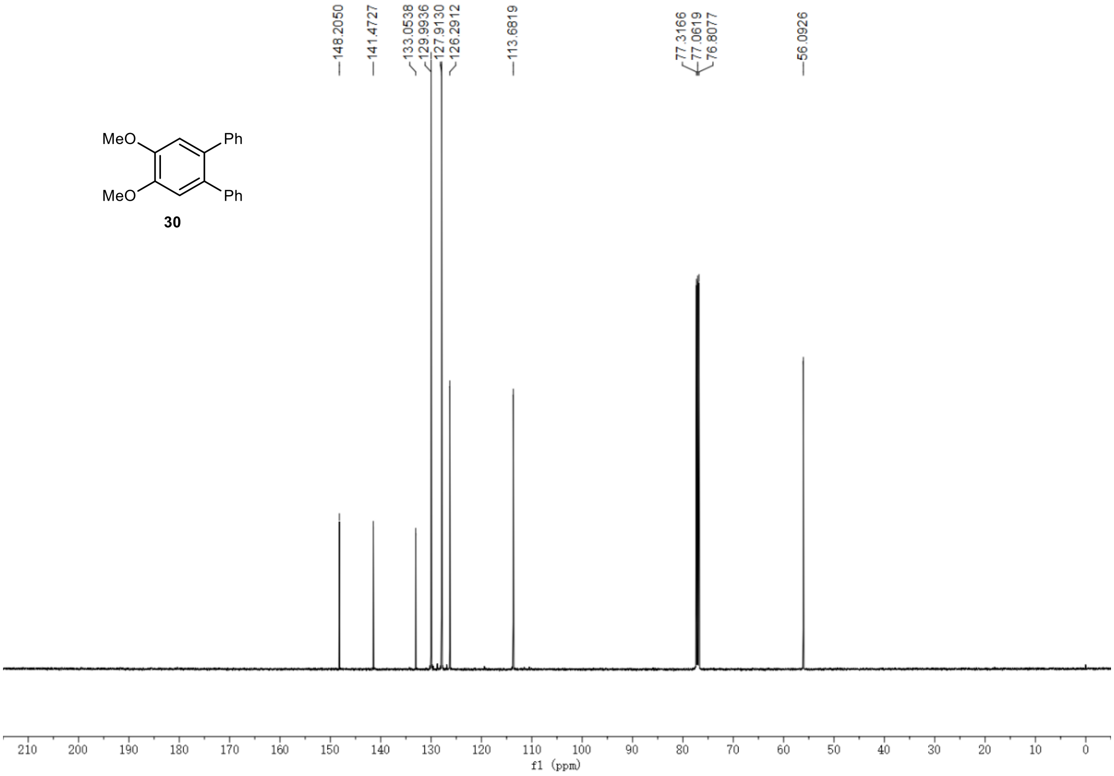

^{1}H NMR (500 MHz, $CDCl_3$)

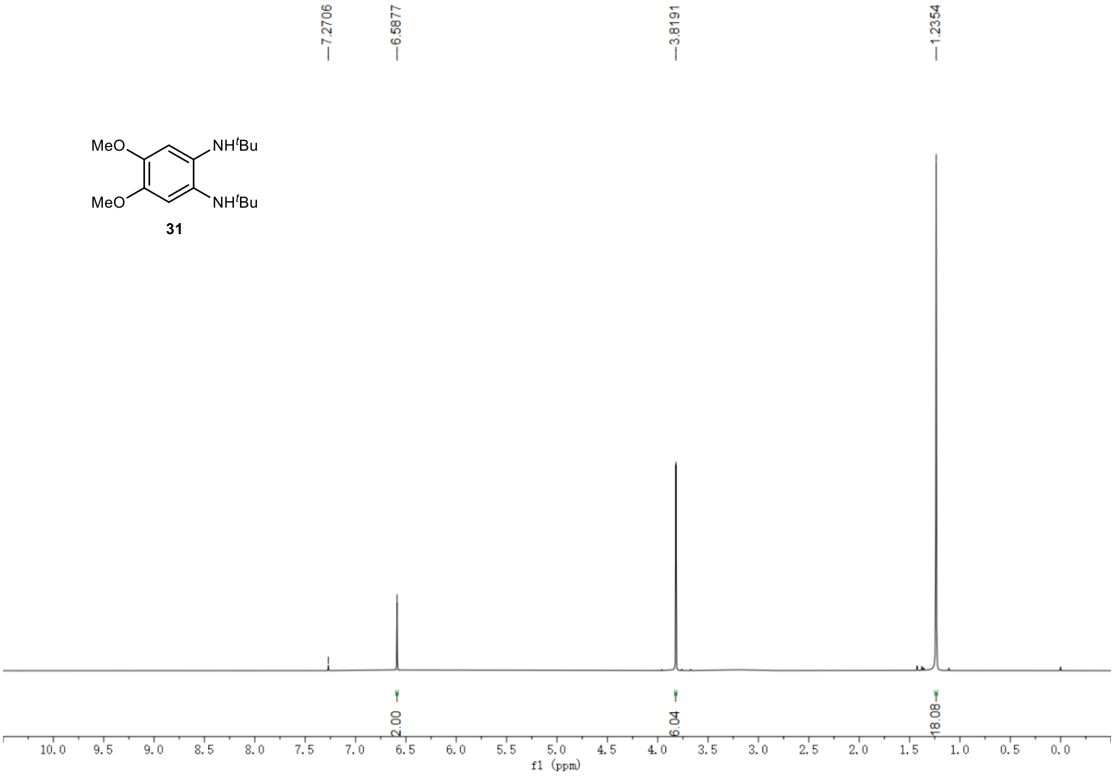

^{13}C NMR (126 MHz, $CDCl_3$)

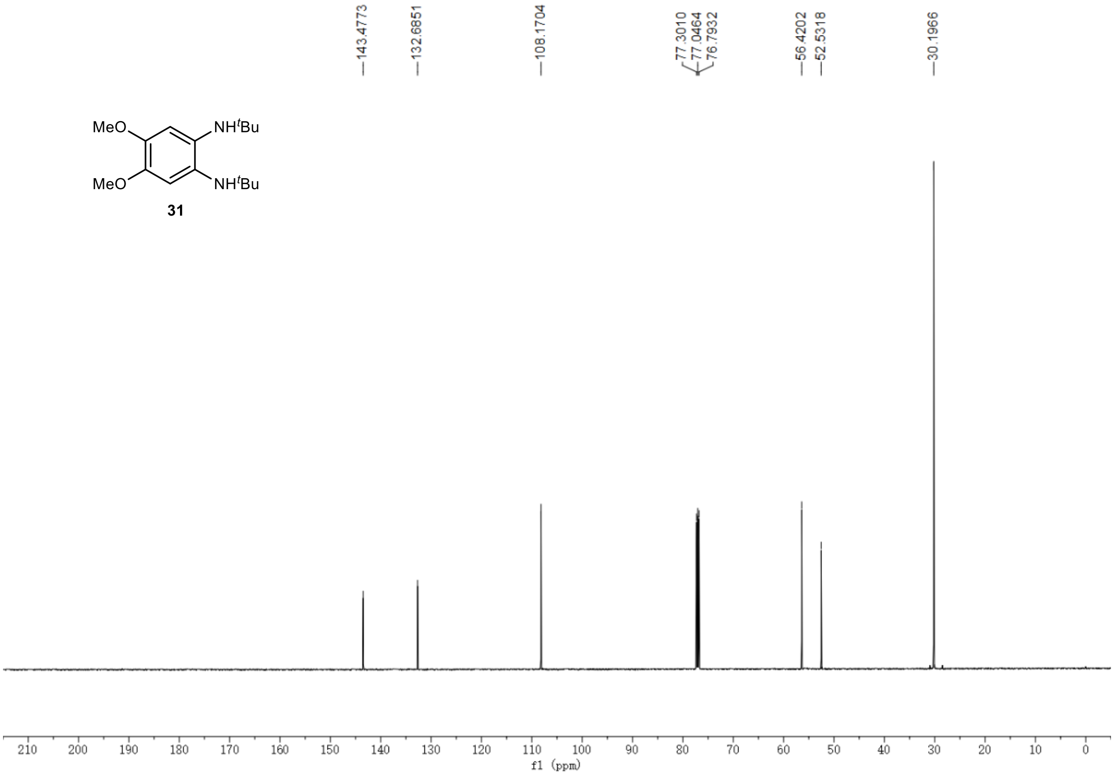

^{1}H NMR (500 MHz, $CDCl_3$)

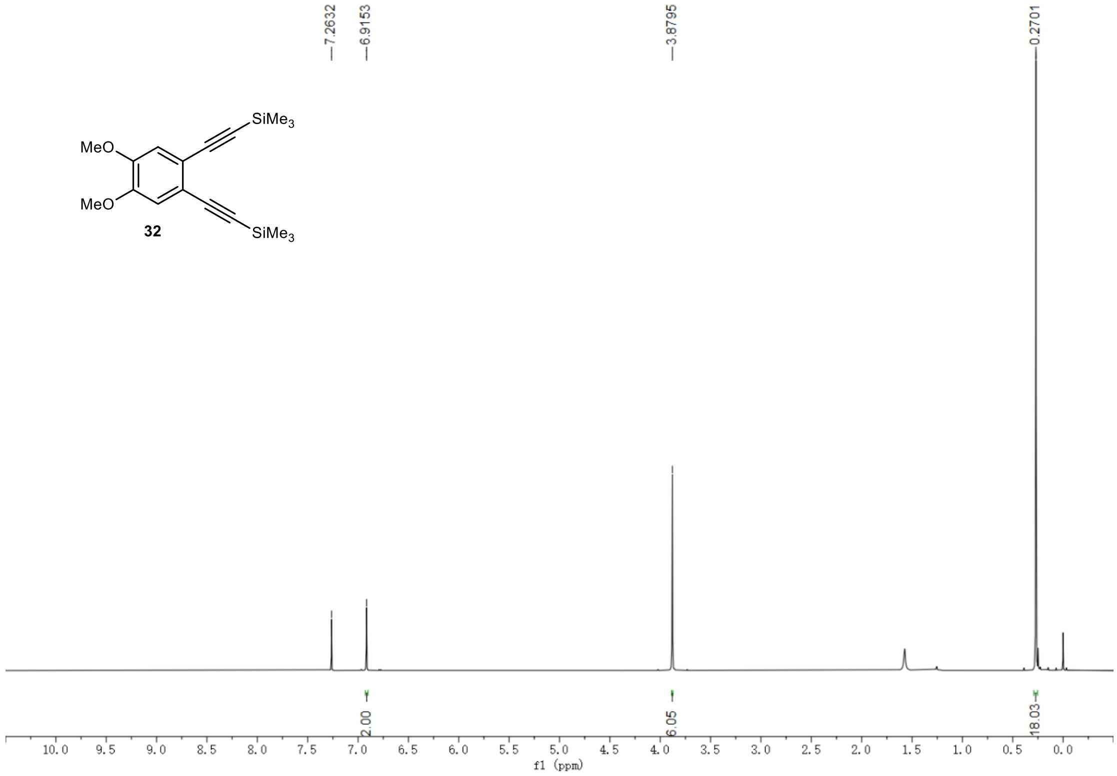

^{13}C NMR (126 MHz, $CDCl_3$)

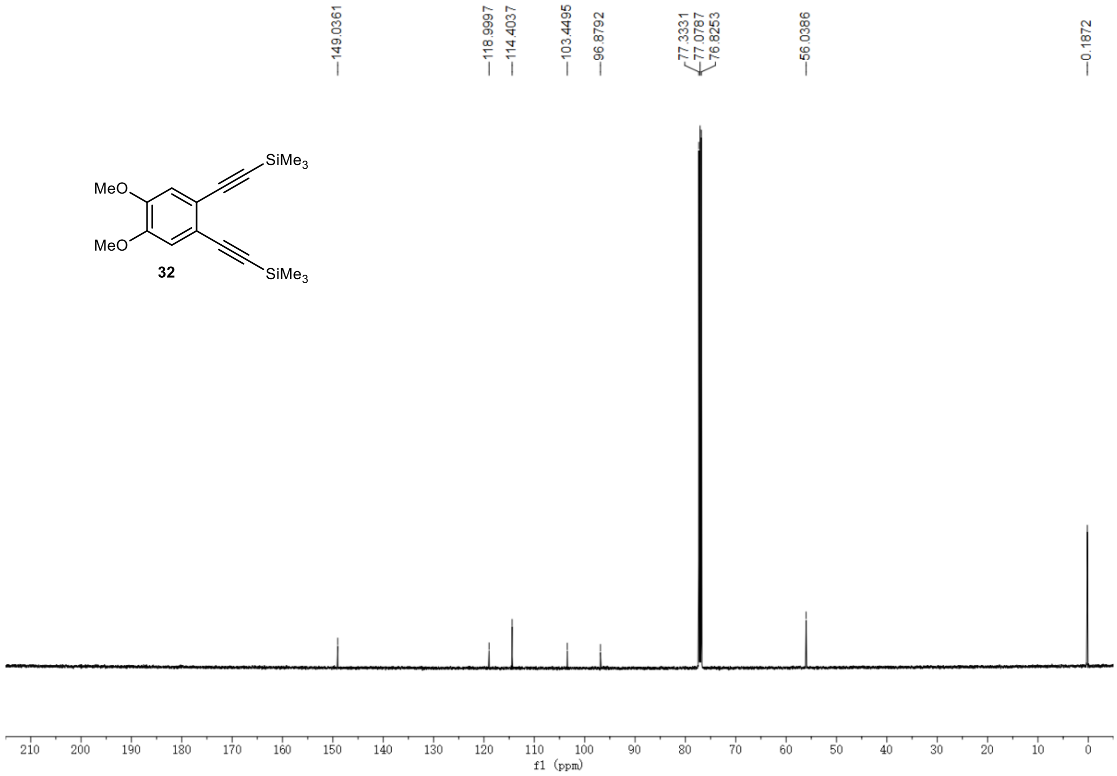

^{1}H NMR (500 MHz, $CDCl_3$)

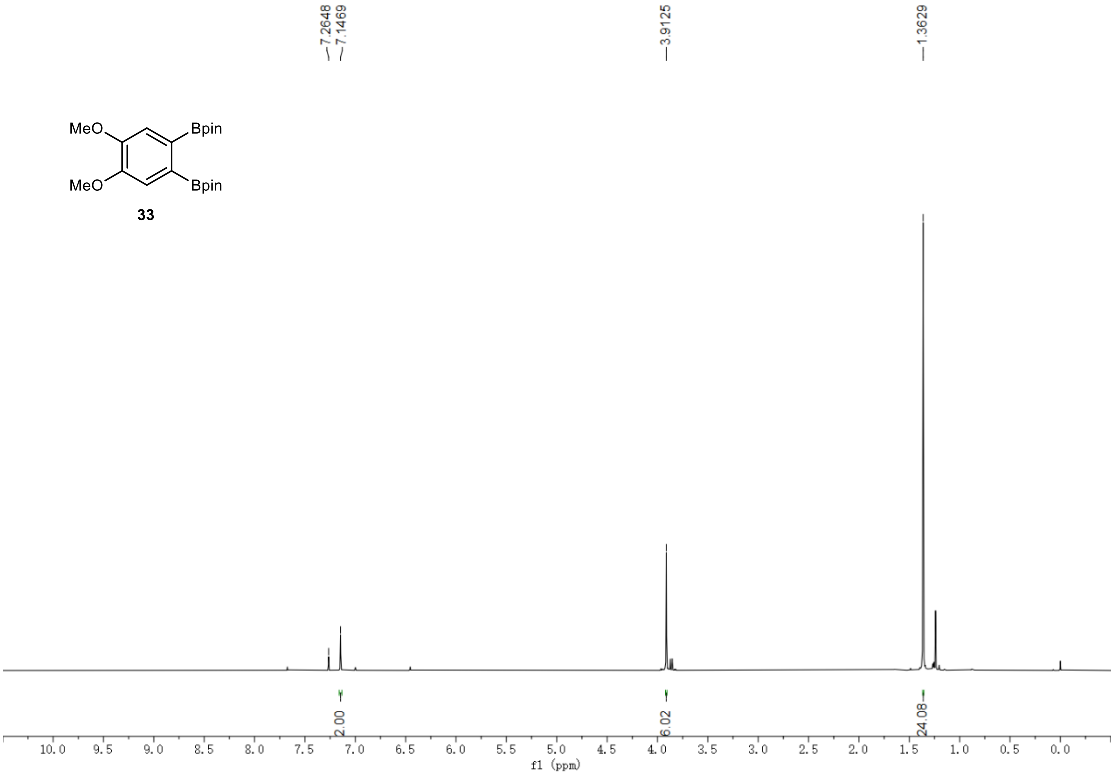

^{13}C NMR (126 MHz, $CDCl_3$)

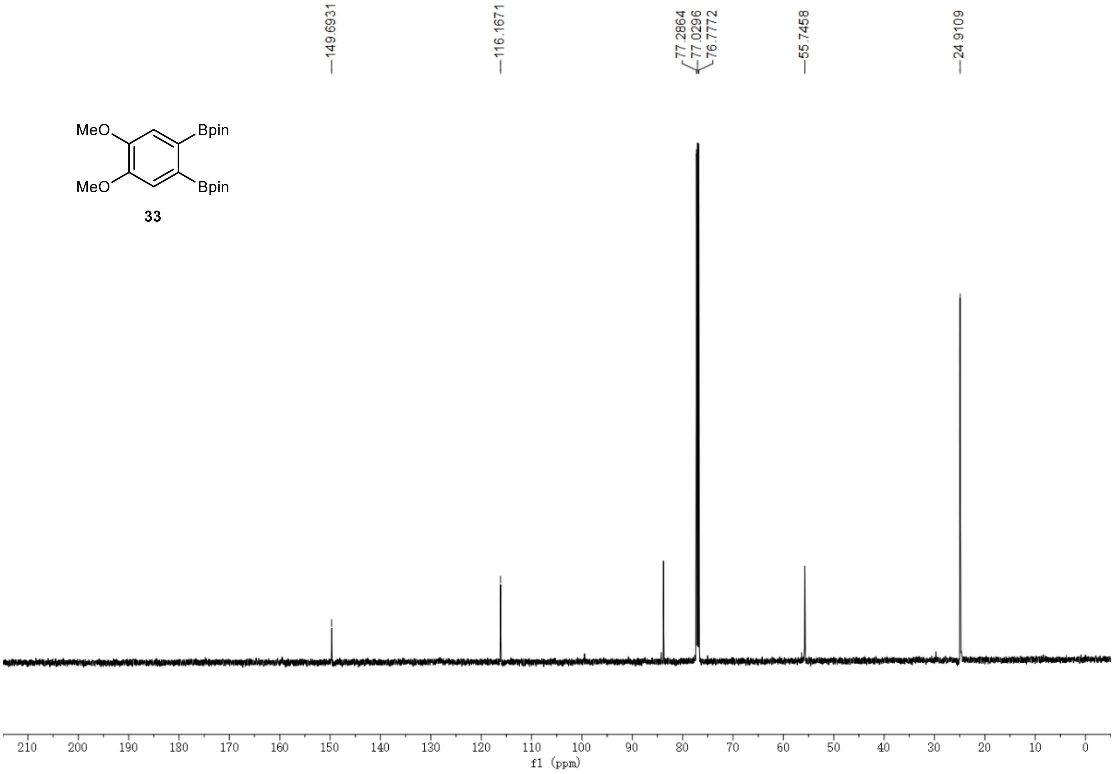

^{1}H NMR (500 MHz, $CDCl_3$)

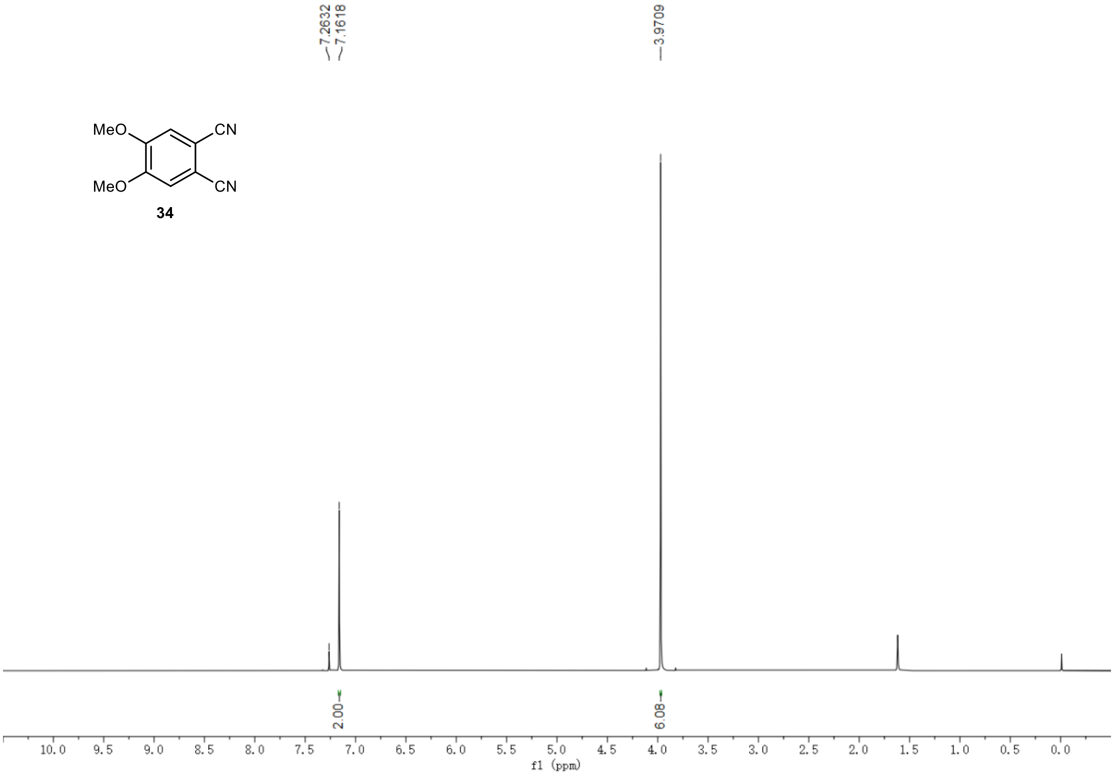

^{13}C NMR (126 MHz, $CDCl_3$)

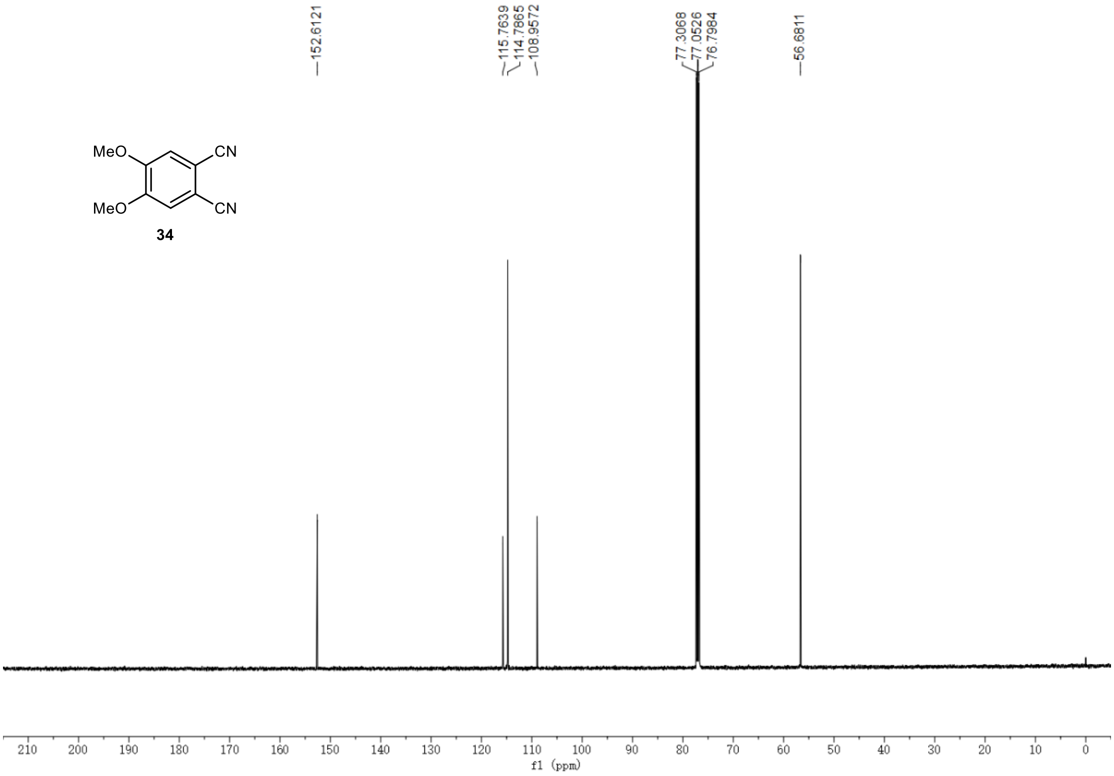

^{1}H NMR (500 MHz, $CDCl_3$)

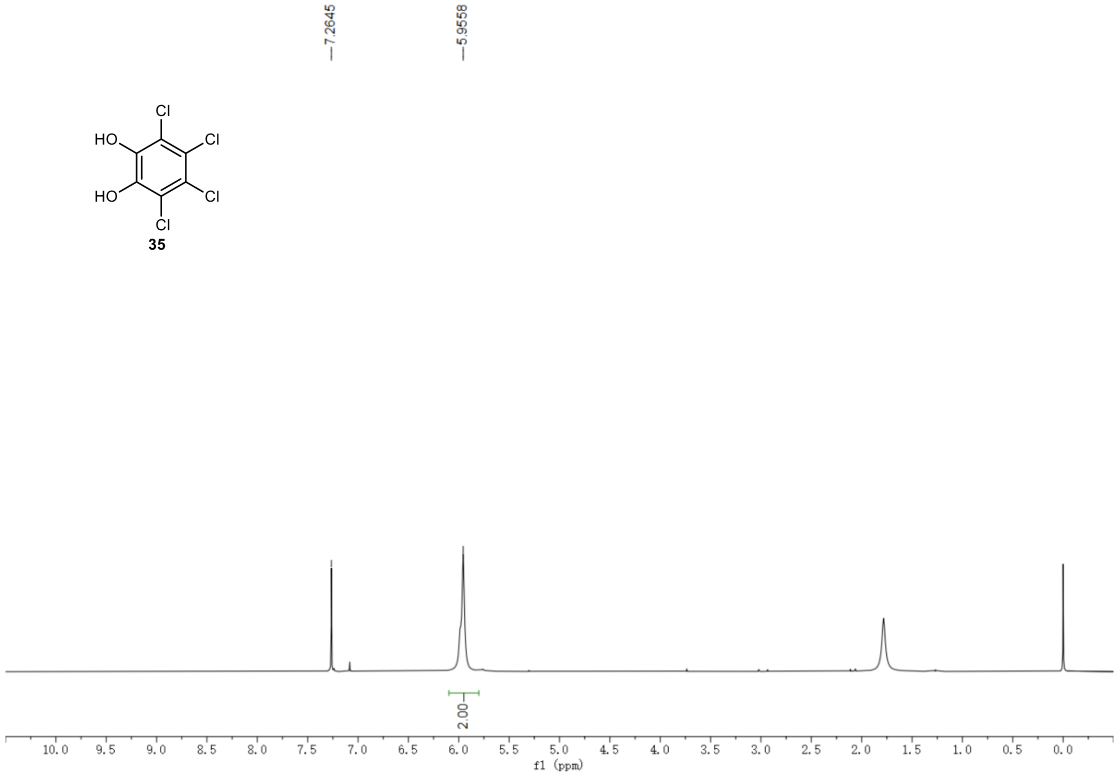

^{13}C NMR (126 MHz, $CDCl_3$)

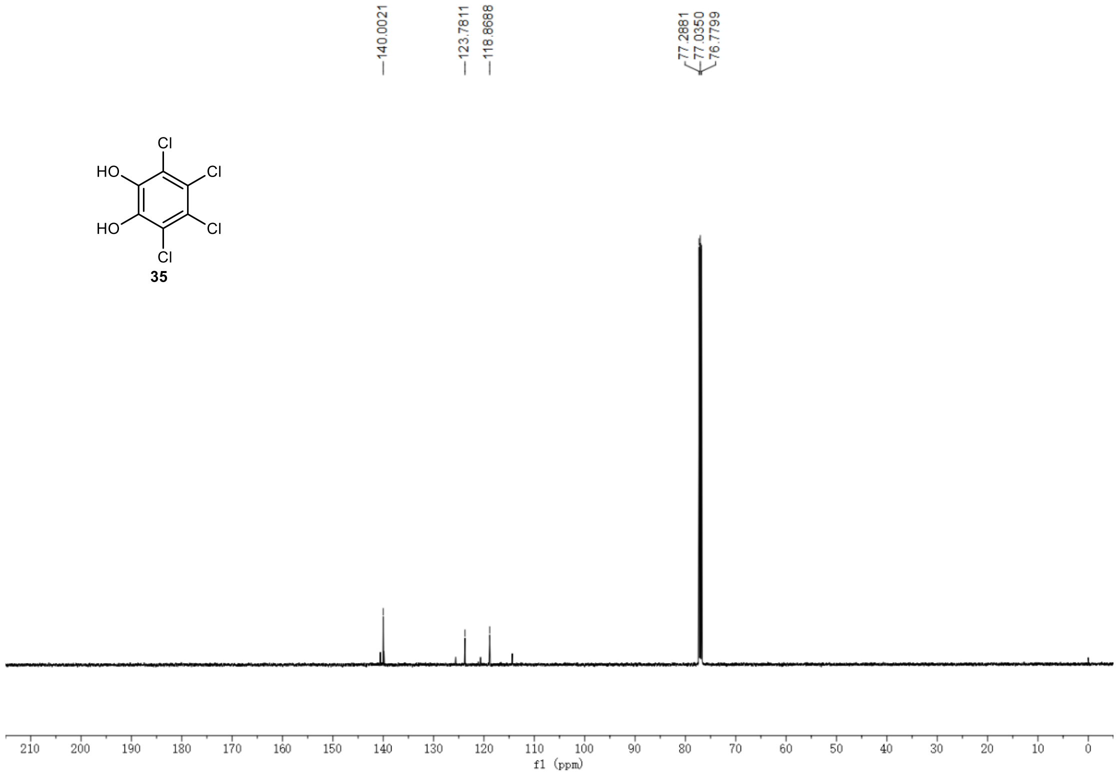

^{13}C NMR (126 MHz, $CDCl_3$)

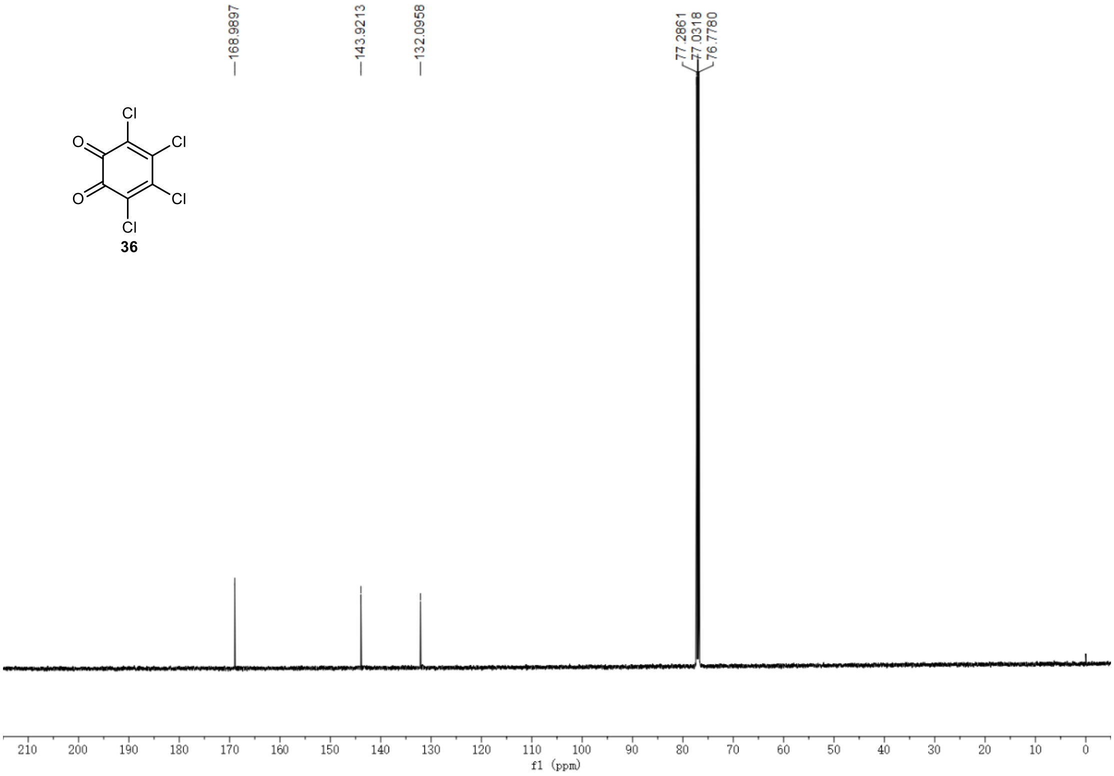

^{1}H NMR (500 MHz, $CDCl_3$)

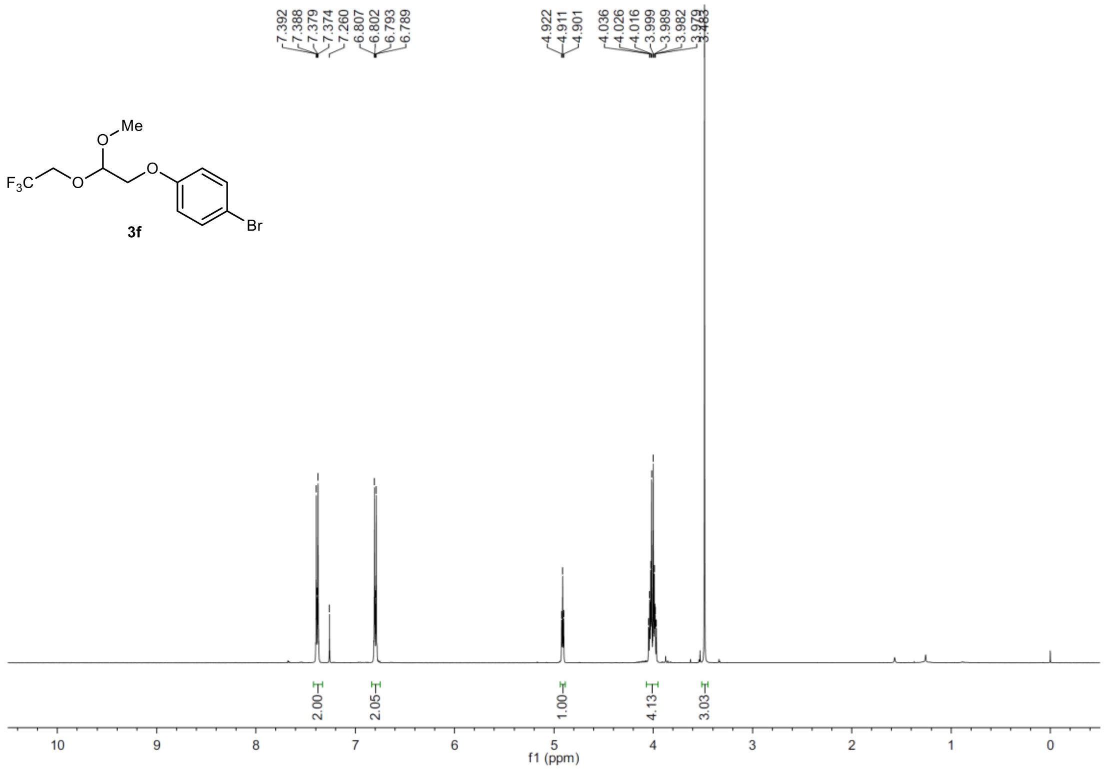

^{13}C NMR (126 MHz, $CDCl_3$)

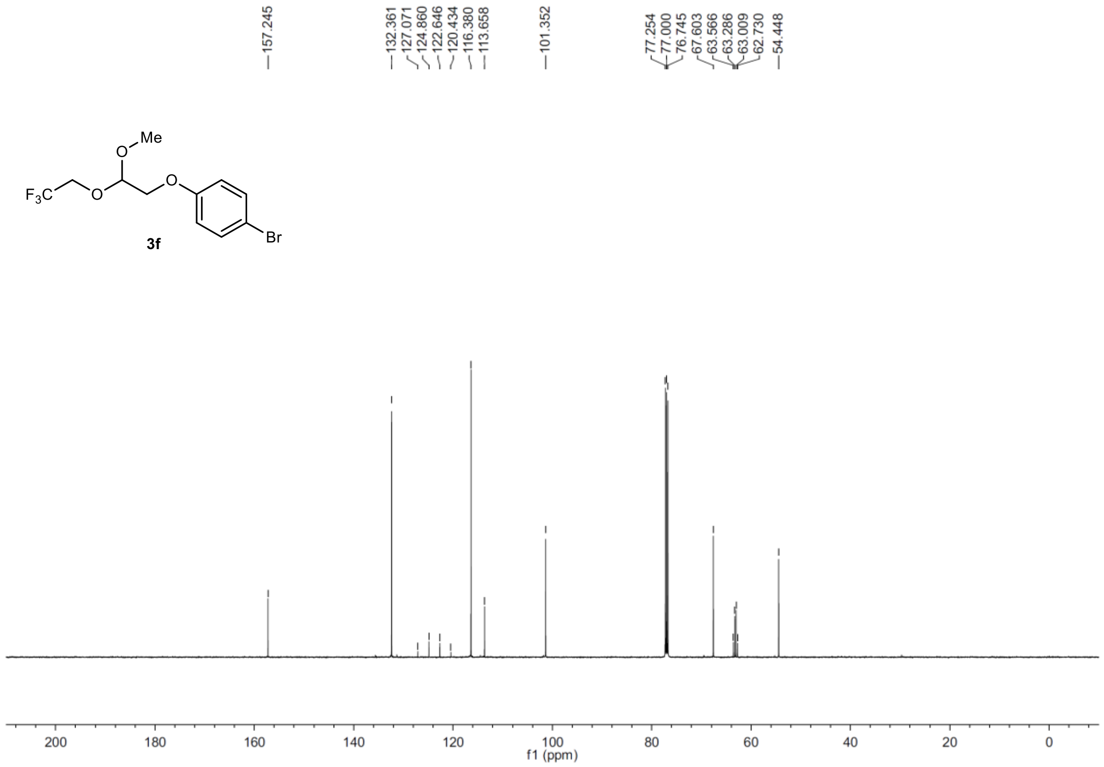

^{1}H NMR (500 MHz, $CDCl_3$)

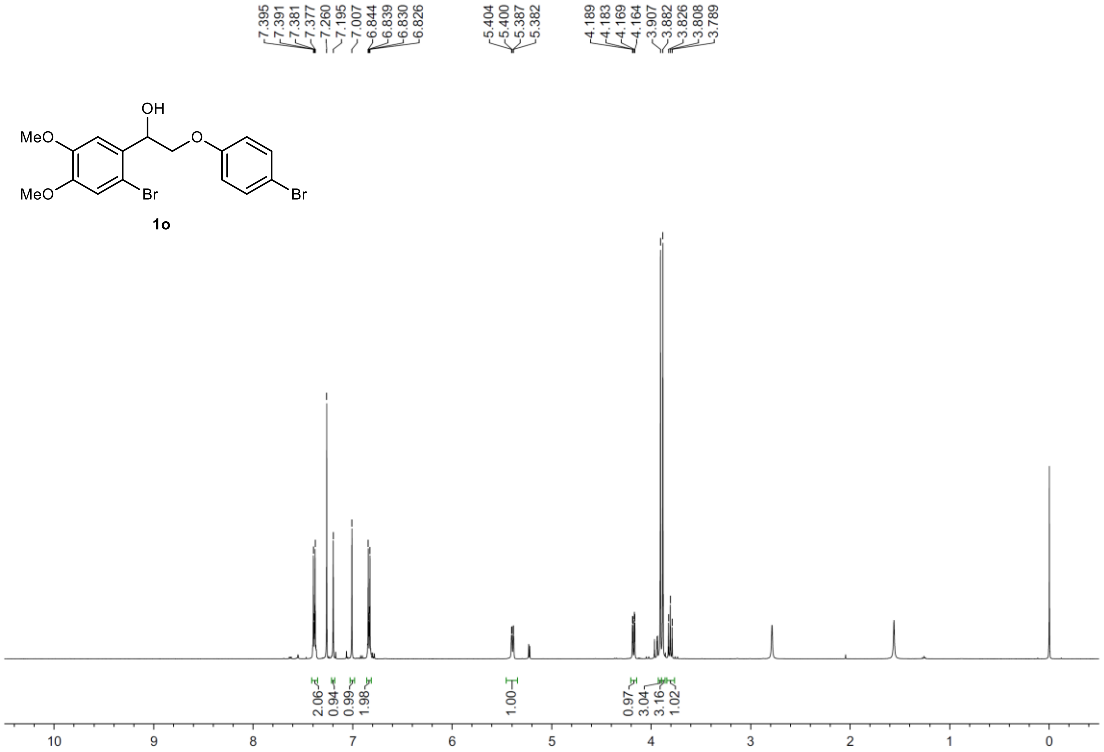

^{13}C NMR (126 MHz, $CDCl_3$)

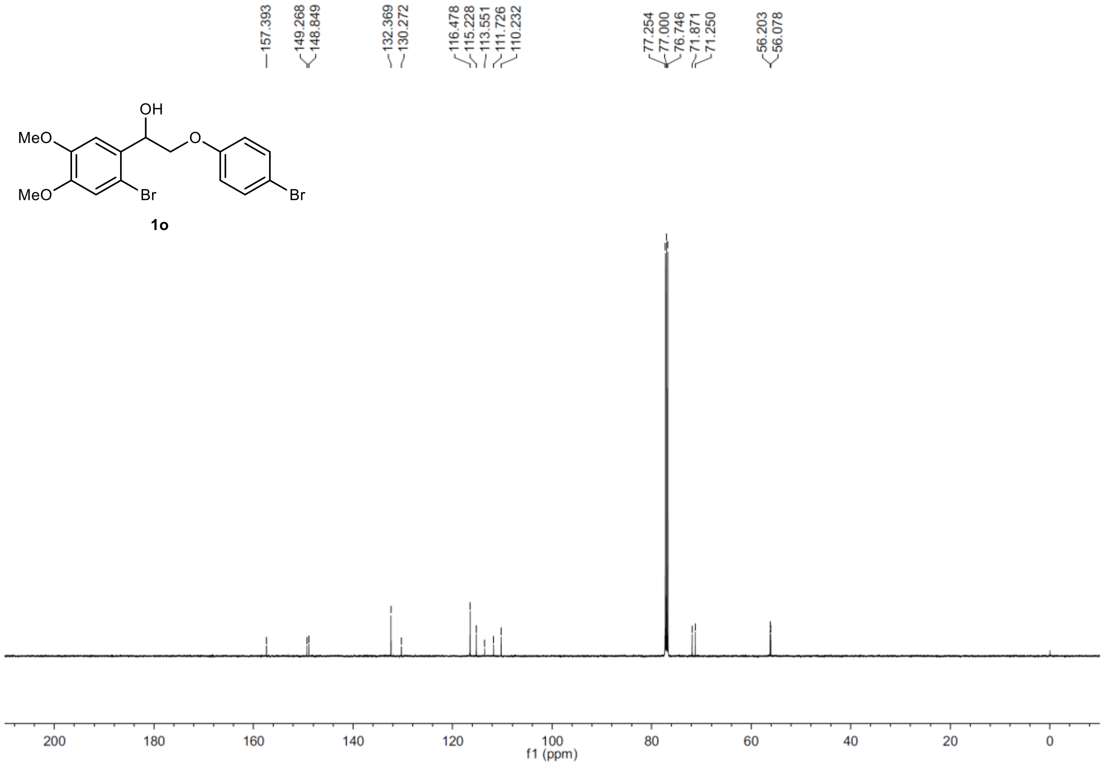

Supplementary References

1. Li, Y. et al. Photocatalytic conversion of lignin models into functionalized aromatic molecules initiated by the proton-coupled electron transfer process. *Org. Lett.* **26**, 1218–1223 (2024).
2. Fleming, M. J. et al. Concise enantioselective total syntheses of (+)-homochelidonine, (+)-chelamidine, (+)-chelidonine, (+)-chelamine and (+)-norchelidonine by a Pd^{II}-catalyzed ring-opening strategy. *Chem. Eur. J.* **14**, 2112–2124 (2008).
3. Yang, S., Chu, M. & Miao, Q. Connecting two phenazines with a four-membered ring: the synthesis, properties and applications of cyclobuta[1,2-b:3,4-b']diphenazines. *J. Mater. Chem. C* **6**, 3651–3657 (2018).
4. Zhou, Y., Akkarasereenon, K., Liu, L., Lin, R., Song, L. & Tong, R. Ecofriendly protocol for ipso-bromination of arylboronic acids. *Org. Lett.* **26**, 5151–5156 (2024).
5. Luo, R.-S., Liao, J.-H., Xie, L., Tang, W.-J. & Chan, A. S. C. Asymmetric ring-opening of oxabenzonorbornadiene with amines promoted by a chiral iridium-monophosphine catalyst. *Chem. Commun.* **49**, 9959–9961 (2013).
6. Wu, H., Zhang, Z., Ma, N., Liu, Q., Liu, T. & Zhang, G. Synthesis of acridines from o-aminoaryl ketones and arylboronic acids by copper trifluoroacetate-mediated relay reactions. *J. Org. Chem.* **83**, 12880–12886 (2018).
7. Henry, M. C., Abbinante, V. & Sutherland, A. Iron-catalyzed regioselective synthesis of 2-arylbenzoxazoles and 2-arylbenzothiazoles via alternative reaction pathways. *Eur. J. Org. Chem.* **2020**, 2819–2826 (2020).
8. Wang, H.-S., Chan, C.-K. & Chang, M.-Y. $Ga(OTf)_3$-mediated synthesis of substituted benzofurans. *Tetrahedron* **72**, 5132–5141 (2016).
9. Wang, J.-X. et al. Scocycamides, a pair of macrocyclic dicaffeoylspermidines with butyrylcholinesterase inhibition and antioxidation activity from the roots of scopolia tangutica. *Org. Lett.* **22**, 8240–8244 (2020).
10. Niphakis, M. J. & Georg, G. I. Synthesis of tylocrebrine and related phenanthroindolizidines by VOF_3-mediated oxidative aryl-alkene coupling. *Org. Lett.* **13**, 196–199 (2011).
11. Reed, J. H., Donets, P. A., Miaskiewicz, S. & Cramer, N. A. 1,3,2-diazaphospholene-catalyzed reductive claisen rearrangement. *Angew. Chem. Int. Ed.* **58**, 8893–8897 (2019).
12. Dumele, O., Wu, D., Trapp, N., Goroff, N. & Diederich, F. Halogen bonding of (iodoethynyl)benzene derivatives in solution. *Org. Lett.* **16**, 4722–4725 (2014).
13. Alfuth, J., Jeannin, O. & Fourmigué, M. Topochemical, single-crystal-to-single-crystal [2+2] photocycloadditions driven by chalcogen-bonding interactions. *Angew. Chem. Int. Ed.* **61**, e202206249 (2022).
14. Alkorbi, F. et al. Complementary syntheses giving access to a full suite of differentially substituted phthalocyanine-porphyrin hybrids. *Angew. Chem. Int. Ed.* **60**, 7632–7636 (2021).

15. Baschieri, A., Amorati, R., Valgimigli, L. & Sambri, L. *J. Org. Chem.* **84**, 13655–13664 (2019).
16. Qarah, A., Gasonoo, M., Do, D. & Klumpp, D. A. Superacid-promoted synthesis of polychlorinated dibenzofurans. *Tetrahedron Lett.* **57**, 3711–3714 (2016).
17. Polidano, K., Reed-Berendt, B. G., Basset, A., Watson, A. J. A., Williams, J. M. J. & Morrill, L. C. Exploring tandem ruthenium-catalyzed hydrogen transfer and SNAr chemistry. *Org. Lett.* **19**, 6716–6719 (2017).
18. Nakazaki, A. et al. Structural study on a naturally occurring terphenyl quinone. *Biosci. Biotechnol. Biochem.* **77**, 1529–1532 (2013).
19. Ma, X. et al. Mild and regioselective bromination of phenols with TMSBr. *Eur. J. Org. Chem.* **28**, 4593–4596 (2019).
20. Wang, T. et al. Hydroxylamine-mediated C-C amination via an aza-hock rearrangement. *Nat. Commun.***12**, 7029 (2021).
21. Moreno-Sanz, G. et al. Synthesis and structure-activity relationship studies of *O*-Biphenyl-3-yl carbamates as peripherally restricted fatty acid amide hydrolase inhibitors. *J. Med. Chem.* **56**, 5917–5930 (2013).
22. Wang, J.-L. et al. Stiochiometric self-assembly of shape-persistent 2D complexes: A facile route to symmetric, supramacromolecular spoked wheels. *J. Am. Chem. Soc.* **133**, 11450–11453 (2011).
23. Wang, T. et al. Halogenated volatiles from the fungus geniculosporium and the actinomycete streptomyces chartreusis. *Beilstein J. Org. Chem.* **9**, 2767–2777 (2013).
24. Pu, X.-Q. et al. *N*-chloro-*N*-methoxybenzenesulfonamide: A chlorinating reagent. *Eur. J. Org. Chem.* **2016**, 5937–5940 (2016).
25. Daferner, M., Anke, T., Hellwig, V., Steglich, W. & Sterner, O. Strobilurin M, tetrachloropyrocatechol and tetrachloropyrocatechol methyl ether: new antibiotics from a Mycena species. *J. Antibiot.* **51**, 816–822 (1998).
26. Zhang, Z. et al. Multiple-stimuli fluorescent responsive metallo-organic helicated cage arising from monomer and excimer emission. *Nat. Commun.* **15**, 7261 (2024).
